# Sleep and Late-Onset Alzheimer’s Disease: Shared Genetic Risk Factors, Drug Targets, Molecular Mechanisms, and Causal Effects

**DOI:** 10.3389/fgene.2022.794202

**Published:** 2022-05-17

**Authors:** Dongze Chen, Xinpei Wang, Tao Huang, Jinzhu Jia

**Affiliations:** ^1^ Department of Biostatistics, School of Public Health, Peking University, Beijing, China; ^2^ Department of Epidemiology and Biostatistics, School of Public Health, Peking University, Beijing, China; ^3^ Key Laboratory of Molecular Cardiovascular Sciences (Peking University), Ministry of Education, Beijing, China; ^4^ Center for Intelligent Public Health, Institute for Artificial Intelligence, Peking University, Beijing, China; ^5^ Center for Statistical Science, Peking University, Beijing, China

**Keywords:** sleep, late-onset Alzheimer’s disease, common genetic etiology, genetic correlation, Mendelian randomization, transcriptome-wide association study

## Abstract

Late-onset Alzheimer’s disease (AD) is associated with sleep-related phenotypes (SRPs). The fact that whether they share a common genetic etiology remains largely unknown. We explored the shared genetics and causality between AD and SRPs by using high-definition likelihood (HDL), cross-phenotype association study (CPASSOC), transcriptome-wide association study (TWAS), and bidirectional Mendelian randomization (MR) in summary-level data for AD (*N* = 455,258) and summary-level data for seven SRPs (sample size ranges from 359,916 to 1,331,010). AD shared a strong genetic basis with insomnia (*r*_g_ = 0.20; *p* = 9.70 × 10^–5^), snoring (*r*_g_ = 0.13; *p* = 2.45 × 10^–3^), and sleep duration (*r*_g_ = −0.11; *p* = 1.18 × 10^–3^). The CPASSOC identifies 31 independent loci shared between AD and SRPs, including four novel shared loci. Functional analysis and the TWAS showed shared genes were enriched in liver, brain, breast, and heart tissues and highlighted the regulatory roles of immunological disorders, very-low-density lipoprotein particle clearance, triglyceride-rich lipoprotein particle clearance, chylomicron remnant clearance, and positive regulation of T-cell–mediated cytotoxicity pathways. Protein–protein interaction analysis identified three potential drug target genes (*APOE*, *MARK4*, and *HLA-DRA*) that interacted with known FDA-approved drug target genes. The CPASSOC and TWAS demonstrated three regions 11p11.2, 6p22.3, and 16p11.2 may account for the shared basis between AD and sleep duration or snoring. MR showed insomnia had a causal effect on AD (OR_IVW_ = 1.02, *P*_IVW_ = 6.7 × 10^–6^), and multivariate MR suggested a potential role of sleep duration and major depression in this association. Our findings provide strong evidence of shared genetics and causation between AD and sleep abnormalities and advance our understanding of the genetic overlap between them. Identifying shared drug targets and molecular pathways can be beneficial for treating AD and sleep disorders more efficiently.

## Introduction

Alzheimer’s disease (AD) is a neurodegenerative disease characterized by progressive memory loss and overall cognitive decline (McKhann et al., 2011) and is highly heritable (heritability 58–79%) (Sims et al., 2020). Growing evidence indicates that AD patients frequently have sleep disorders, implying common causes of these complex phenotypes. Emerging epidemiological studies suggest that AD is associated with a significantly increased risk of sleep disorders and vice versa (Andrade et al., 2018; Brzecka et al., 2018; Shi et al., 2018; Sadeghmousavi et al., 2020). Furthermore, neuropathological studies have shown that extracellular levels of both Aβ and tau fluctuate during the normal sleep-wake cycle (Wang and Holtzman, 2020). In animal models, sleep disturbance and increased arousal lead to increased Aβ production and decreased Aβ clearance, while chronic increased arousal promotes Aβ aggregation and deposition, thus leading to sleep disturbance (Wang and Holtzman, 2020). Importantly, amyloid β and tau protein, which are core hallmarks of AD, can exacerbate the sleeping status sleep disorder in an AD person (Liu et al., 2019). Taken together, we hypothesized that there might be a shared genetic basis underlying these connections between AD and sleep disorders.

Genome-wide association studies (GWASs) have yielded new insights into the genetics of AD (Iris E. Jansen et al., 2019; Kunkle et al., 2019) and SRPs (Dashti et al., 2019; Lane et al., 2019; Philip R. Jansen et al., 2019; Campos et al., 2020). Despite the large sample sized GWAS cohorts, the identified genome-wide loci account for only a small portion of the variance of AD and sleep disorders (Manolio et al., 2009). The combined effects of whole-genome single nucleotide polymorphisms (SNPs), including those that do not reach genome-wide significance (Boyle et al., 2017) and shared genetic architecture are expected to account for the missing heritability. However, no cross-trait genome-wide study has been conducted to quantify the level of genetic overlap and identify the shared loci between AD and sleep disorders.

Therefore, to improve our understanding of genetic overlap and causality and to identify genomic loci shared between AD and sleep disorders, we conducted a large-scale cross-trait genome-wide study to explore genetic correlations and shared genetic components among these complex phenotypes using data from the Psychiatric Genomic Consortium (PGC)^9^ and the Complex Trait Genetics Lab (CTGlab)^11^. We further compared shared genes between AD and insomnia with known target genes of AD and insomnia meditation using protein–protein interaction analysis, which may provide insights into potential target genes for identifying drug targets.

## Materials and Methods

### Study Design, Data Source, and Study Population

**FIGURE 1:**
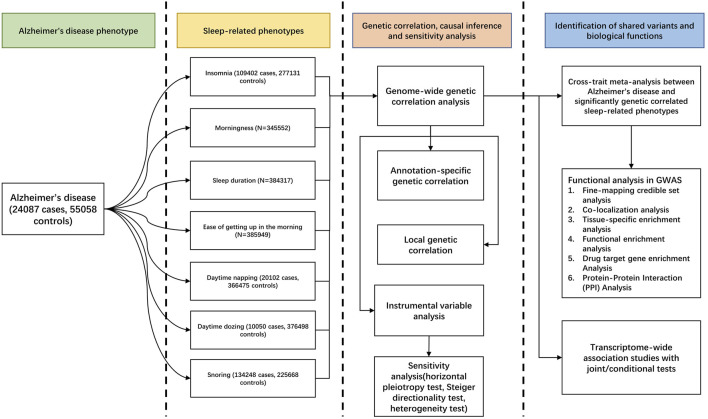
Workflow of the study.

Our overall study design is shown in Figure 1. We used the GWAS summary-level data from PGC and CTGlab for AD and seven SRPs (Iris E. Jansen et al., 2019; Philip R. Jansen et al., 2019) (Supplementary Table S1). The AD/AD-by-proxy meta-analysis summary statistics combined 71,880 cases and 383,378 controls from four cohort-level GWASs, including the Alzheimer workgroup initiative of the Psychiatric Genomic Consortium (PGC-ALZ), the International Genomics of Alzheimer’s Project (IGAP), the Alzheimer’s Disease Sequencing Project (ADSP), and UKB, whereas the SRP meta-analysis summary statistics combined ∼13,31,010 participants from the UKB and 23 and Me. In the original GWASs, AD cases were diagnosed according to the recommendations of the National Institute on Aging–Alzheimer’s Association (NIAAA) criteria, the National Institute of Neurological and Communicative Disorders and Stroke and the Alzheimer’s Disease and Related Disorders Association (NINCDS-ADRDA) criteria, or the International Classification of Diseases (ICD-10) criteria^27^. Details of the definition of each self-reported SRP are present in Supplementary Table S2. All participants were of European ancestry, with no overlapping samples (Supplementary Methods).

### Genetic Correlation Analysis

To evaluate genetic correlation (***r***_g_) between AD and SRPs, we used the conventional cross-trait linkage disequilibrium score regression (LDSC) (Bulik-Sullivan et al., 2015) and the more recent high-definition likelihood (HDL) method (Ning et al., 2020). As the HDL method yields more precise estimates of genetic correlations than LDSC, we chose the HDL method as the main result. The HDL method uses the LD reference computed from 335,265 genomic British individuals in the United Kingdom Biobank (UKB) (Ning et al., 2020). The *p* value was corrected by the Bonferroni procedure (*P*_Bonferroni_ < 0.05).

### Annotation-Specific Genetic Correlation

We calculated an annotation-specific genetic correlation using genetic covariance analyzer (GNOVA) software (Lu et al., 2017). As with LDSC, GNOVA is able to statistically correct for any sample overlap between two different sets of GWAS summary statistics. Compared to LDSC, GNOVA provides greater statistical power and higher estimation accuracy, especially in the case of moderate correlations (Lu et al., 2017). We estimated the genetic correlation across ∼5 million well-imputed SNPs in the 1,000 Genomes Project and partitioned the estimates among categories of SNPs defined by 20 functional categories implicated in open chromatin, histone modification, and transcription factor binding sites (TFBSs) (Lu et al., 2015), 22 autosome annotations (Lu et al., 2015), ten broadly defined tissue type annotations (Lu et al., 2016), and 66 epigenetic cell types (Lu et al., 2016). Using 0.5 as the cutoff, we converted continuous annotation scores into binary annotation scores (i.e., 0 and1). A detailed description of these different subdivisions can be found in (Supplementary Tables S3–S5).

### Local Genetic Correlation

We estimated local genetic correlations between AD and related SRPs in 1703 pre-specified LD-independent segments with a super genetic covariance analyzer (SUPERGNOVA) (Zhang et al., 2020). This method is designed to identify small contiguous regions of the genome where genetic associations are locally consistent with two traits. SUPERGNOVA quantifies the local genetic correlation and *p*-values (*P*_SuperGnova_) between pairs of traits in local regions. SUPERGNOVA has stronger statistical performance than Heritability Estimation from Summary Statistics (HESS) (Zhang et al., 2020), which is not robust to incorrectly specified overlapping sample sizes and is subject to type I error inflation when inaccurate overlapping sample sizes and phenotypic correlation values are provided. AD and SRPs were considered to have a genetic correlation in the local region if *P*_SUPERGNOVA_ was significant after correcting for suggestive *p*-value (*P*_SUPERGNOVA_ < 1.0 × 10^–4^).

### Cross-Trait Meta-Analysis

We conducted a pairwise cross-trait meta-analysis using Cross Phenotype Association (CPASSOC). CPASSOC combines effect estimates and standard errors of GWAS summary statistics to test the hypothesis of association between a SNP and two traits. (Zhu et al., 2015). We used the heterogonous version of cross-phenotype association (SHet) that is based on a sample size-weighted, fixed-effect model and is more powerful when there is a heterogonous effect present between studies (Zhu et al., 2018). The cross-trait meta-analysis was not inflated (Supplementary Figures S1–S3, Supplementary Methods).

### Fine-Mapping Credible Set Analysis

For each of the shared loci between AD and SRPs that meet the cross-trait meta-analysis significance criteria, we extracted variants within 500 kb of the index SNP and then identified a 99% credible set of causal SNPs using the Bayesian likelihood fine-mapping algorithm (FM-summary) (Farh et al., 2015).

### Co-Localization Analysis

We extracted summary statistics for variants within 500 kb of the index SNP at each of the shared loci between AD and SRPs and used the R ‘coloc’ package to perform genetic co-localization analysis, which calculated the probability that the two traits shared a common genetic causal variant. In our study, we considered loci with a probability (H4) greater than 0.4 to be co-localized (Giambartolomei et al., 2014).

### Tissue-Specific Enrichment Analysis

To test if shared gene sets identified from cross-trait meta-analysis were highly enriched or specifically expressed in tissues, we conducted tissue-specific enrichment analysis (TSEA) by using the R ‘TissueEnrich’ package (Jain and Tuteja, 2019). The *p* value was corrected by the Benjamini–Hochberg program.

### Functional Enrichment Analysis

To obtain biological insights for identified shared genes (***P***_meta_<1.67 × 10^–8^) from the cross-trait meta-analysis, we used the plug-in ClueGO (version: 2.5.7) of the Cytoscape (version: 3.8.2) tool to access enrichment of the gene sets in the Gene Ontology (GO) biological process (18,483 terms\pathways with 17,972 available unique genes) and Kyoto Encyclopedia of Genes and Genomes (KEGG) pathways (328 terms\pathways with 8,024 available unique genes) and displayed the relationship between genes and GO\KEGG terms (Shannon et al., 2003; Bindea et al., 2009). The Bonferroni procedure was used to account for multiple testing (*P*_Bonferroni_ < 0.05).

### Drug Target Gene Enrichment Analysis

We queried the Therapeutic Target Database (TTD) to identify Food and Drug Administration (FDA)-approved drugs that were used for AD and insomnia (Wang et al., 2020). Meditation target genes for AD and insomnia were extracted from the DrugBank 5.0 database (Wishart et al., 2018), respectively.

### Protein–Protein Interaction Analysis

We searched the STRING (version: 11.0) database to identify protein–protein interactions (PPIs) among AD drug target genes and insomnia drug target genes and identified AD and insomnia shared genes from CPASSOC (Szklarczyk et al., 2019). We selected *Homo sapiens* as the organism and considered total scores above 0.40 (medium confidence) to correspond to the combination of the following three different scores: co-expression, experimental, and text mining.

### Mendelian Randomization Analysis

To examine evidence for potential causal relationships between AD and genetically associated SRPs, we conducted instrumental variable analysis using bi-directional Mendelian randomization (MR) implemented in two-sample MR (TSMR, version: 0.5.6) (Hemani et al., 2018) and used inverse-variance weighting (IVW) as the primary method (Hemani et al., 2017). Furthermore, as horizontal pleiotropy is an important confounder that could bias the estimates and often results in an inflated test statistic in MR analysis, we used MR-Egger regression (Bowden et al., 2015) and MR-Pleiotropy Residual Sum and Outlier (MR-PRESSO) methods (Verbanck et al., 2018) to detect horizontal pleiotropy. *p*-values were corrected for multiple testing using the Bonferroni procedure(*p* < 0.05/3). Given the strong association between different SRPs and the significant genetic similarity between some psychiatric disorders (e.g., major depression (MDD)) and sleep abnormalities, we used multivariate MR (MVMR) (Sanderson et al., 2021) to assess the direct effect of genetic susceptibility to insomnia, sleepdur, and MDD on AD. We conducted sensitivity analyses using weighted median, simple median, MR-Steiger, and MR-Robust Adjusted Profile Scores (MR-RAPS). We applied MR-Steiger to assure that the causal direction between the hypothesized exposure and outcome was correctly assigned (Hemani et al., 2017). Considering the measurement error in SNP-exposure effects, the MR-RAPS is unbiased when there are many weak instruments and is robust to systematic and idiosyncratic pleiotropy (Zhao et al., 2020). A heterogeneity test was also performed to determine that each SNP has the same effect on the results. If exposure is a binary variable, we interpreted the causal estimates as the average change in outcome per doubling (2-fold increase) in the odds of exposure, which could be obtained by multiplying the causal estimate by 0.693 (log_*e*_2) (Burgess and Labrecque, 2018).

### Transcriptome-Wide Association Studies

To identify shared genes revealing the shared mechanisms of genetic correlations between AD and SRPs, we next traced down to the gene level to evaluate tissue-specific expression-trait associations and the shared expression-trait associations between AD and each genetically correlated SRP using transcriptome-wide association studies (TWAS method: FUSION) (Gusev et al., 2016). The Benjamini–Hochberg (BH) procedure was applied to identify significant expression-trait associations adjusted for multiple comparisons for all gene-tissue pairs tested for each trait (∼230,000 gene tissue pairs in total, significant expression-trait associations were defined as *P*_BH_ < 0.05). We further tested for conditional relationships among the shared genes to identify an independent set of gene-based genetic models using an extension of TWAS that leverages previous methods for joint/conditional tests of SNPs using summary statistics (Gusev et al., 2018) (Supplementary Material).

## Results

### Genetic Correlations of AD With SRPs

**FIGURE 2:**
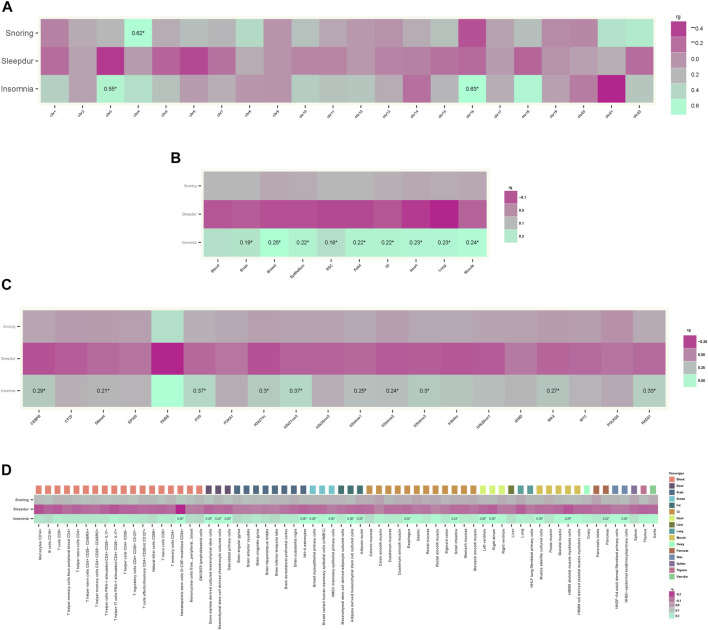
Annotation-specific genetic correlation. **(A)** Annotation-specific genetic correlation between AD and SRPs by 22 chromosomes. Colors represent the estimated annotation-specific genetic correlation between AD and SRPs (Insomnia, Sleepdur, and Snoring) using GNOVA; aquamarine for positive genetic correlation and violet red for negative genetic correlation at the corresponding annotation. The significant effects are labeled with ***r***_**g**_ and “*” (***p*** < 0.05/66). **(B)** Annotation-specific genetic correlation between AD and SRPs by ten tissues. Colors represent the estimated annotation-specific genetic correlation between AD and SRPs (Insomnia, Sleepdur, and Snoring) using GNOVA; aquamarine for positive genetic correlation and violet red for negative genetic correlation at the corresponding annotation. The significant effects are labeled with ***r***_**g**_ and “*” (***p*** < 0.05/30). **(C)** Annotation-specific genetic correlation between AD and SRPs by 20 functional categories. Colors represent the estimated annotation-specific genetic correlation between AD and SRPs (Insomnia, Sleepdur, and Snoring) using GNOVA; aquamarine for positive genetic correlation and violet red for negative genetic correlation at the corresponding annotation. The significant effects are labeled with ***r***_**g**_ and “*” (***p*** < 0.05/60). **(D)** Annotation-specific genetic correlation between AD and SRPs by 66 epigenetic cell types. Colors represent the estimated annotation-specific genetic correlation between AD and SRPs (Insomnia, Sleepdur, and Snoring) using GNOVA, aquamarine for positive genetic correlation and violet red for negative genetic correlation at the corresponding annotation. The significant effects are labeled with ***r***_**g**_ and “*” (***p*** < 0.05/198).

There was a positive overall positive genetic correlation of AD with insomnia (***r***_g_ = 0.20; ***p*** = 9.70 × 10^–5^) and snoring (***r***_g_ = 0.13; ***p*** = 2.45 × 10^–3^) and a negative genetic correlation with sleep duration (***r***_g_ = −0.11; ***p*** = 1.18 × 10^–3^) using HDL (Table 1). No significant genetic correlations of AD with daytime dozing, ease of getting up in the morning, morningness, and daytime napping were observed. Annotation-specific genetic correlation analyses showed that shared effects were concentrated in some chromosomes with the strongest positive genetic correlation at chr16 (***r***_g_ = 0.63; ***p*** = 1.24 × 10^–4^) between AD and insomnia and at chr4 (***r***_g_ = 0.62; ***p*** = 1.48 × 10^–4^) between AD and snoring (Figure 2A). We also observed a stronger genetic correlation at open chromatin, histone modification, and TFBS regions between AD and insomnia (Figure 2C). Figures 2B,D exhibit consistent results with the GNOVA estimate, with evidence of a positive genetic correlation in bone, breast, brain, heart, gastrointestinal, muscle, pancreas, adipose, skin, fetal, lung, and embryonic stem cell tissues between AD and insomnia.

**TABLE 1:** Genetic correlation of Alzheimer’s disease with related sleep-related phenotypes estimated by the high-definition likelihood method and linkage disequilibrium score regression.

Method	Trait	rg	rg, SE	rg, 95% CI	*p* value	h^2 (SE)
HDL	Dozing	0.10	0.066	−0.03 to 0.23	1.49E-01	0.01 (0.001)
	Getting up	−0.02	0.038	−0.09 to 0.05	6.61E-01	0.07 (0.003)
	Insomnia	0.20	0.052	0.10 to 0.30	9.70E-05	0.04 (0.002)
	Morningness	0.00	0.035	−0.07 to 0.07	9.67E-01	0.11 (0.003)
	Napping	0.16	0.067	0.03 to 0.29	1.61E-02	0.02 (0.001)
	Sleepdur	−0.11	0.035	−0.18 to −0.04	1.18E-03	0.07 (0.002)
	Snoring	0.13	0.042	0.05 to 0.21	2.45E-03	0.06 (0.002)
LDSC	Dozing	0.12	0.096	−0.07 to 0.31	2.26E-01	0.01 (0.001)
	Getting up	−0.05	0.051	−0.15 to 0.05	3.15E-01	0.07 (0.003)
	Insomnia	0.22	0.077	0.07 to 0.37	5.00E-03	0.05 (0.002)
	Morningness	−0.03	0.045	−0.12 to 0.06	5.64E-01	0.11 (0.004)
	Napping	0.17	0.086	0.00 to 0.34	4.52E-02	0.02 (0.002)
	Sleepdur	−0.12	0.051	−0.22 to −0.02	2.15E-02	0.07 (0.003)
	Snoring	0.10	0.058	−0.01 to 0.21	7.80E-02	0.06 (0.003)

Note: Summary statistics for each trait were merged with Hapmap3 SNPs excluding the HLA region to estimate rg. *p* value <0.05/7. Dozing, daytime dozing; Getting up, ease of getting up in the morning; Sleepdur, sleep duration.

The local genomic regions around individual AD loci from GWASs showed signals of genetic overlap with related SRPs. Accounting for correction, there was a genome-wide significant local genetic correlation between AD and SRPs at three regions (chr16:29036613-31382943 harboring previous AD locus *GDPD3* for AD with insomnia and snoring; chr19:4348967-5811852 harboring previous AD locus *APOE* for AD and insomnia; and chr2:201576284-202818637 harboring previous migraine locus *CDK15* for AD and sleep duration) using SUPERGNOVA (*P*_SuperGnova_ < 1.0 × 10^–4^).

### Cross-Trait Meta-Analysis Between AD and SRPs

In total, we identified 31 independent loci shared between AD and three genetically correlated SRPs (***p***_meta_ < 1.67 × 10^–8^ and single-trait ***p*** < 1 × 10^–3^) (Table 2). The credible set of SNPs for each of these shared loci was also identified (Supplementary Tables S6–S8). Among the 31 independent shared loci, two colocalized at the same candidate causal variant within each variant (rs12292911 and rs3121427) (Supplementary Table S9).

**TABLE 2:** Cross-trait meta-analysis results between Alzheimer’s disease and sleep-related phenotypes (*P*_meta_ < 1.67 × 10^–8^ and single-trait *p* < 1 × 10^–3^).

Model	Index.SNP	CHR	Genome position	A1	A2	P1	P2	P meta value	Genes within the clumping region	Variant annotation
AD_Insomnia	rs11234556	11	11q14.2	T	C	3.92E-17	3.75E-03	8.706E-17	[*PICALM*]	intergenic
	rs150567157	19	19q13.32	T	C	6.51E-21	9.43E-03	1.11E-20	[*PPP1R37*]	intron
	rs186110295	19	19q13.32	T	G	4.45E-11	5.56E-03	1.583E-11	[*BCL3*]	upstream
	rs2249152	19	19p13.3	T	C	9.43E-05	2.55E-06	9.532E-09	[*KDM4B*]	intron
	rs606757	19	19q13.32	C	A	4.16E-12	7.34E-03	1.193E-11	[*MARK4*]	intron
	rs6857	19	19q13.32	T	C	0	4.48E-03	0	[*APOC1*, *APOE*, *PVRL2*, and *TOMM40*]	3_prime_UTR
	rs9268428	6	6p21.32	T	G	1.24E-09	1.41E-03	3.194E-11	[*BTNL2*, *HCG23*, *HLA-DRA*, *HLA-DRB1*, *HLA-DRB5*, and *HLA-DRB6*]	intergenic
AD_Sleepdur	rs1081105	19	19q13.32	C	A	1.16E-232	4.73E-03	8.459E-241	[*APOC1*, *APOE*, *PVRL2*, and *TOMM40*]	upstream
	rs11672748	19	19q13.32	G	A	7.25E-24	3.24E-03	1.013E-23	[*APOC2*, *APOC4*, *APOC4-APOC2*, *CLPTM1*, and *RELB*]	intron
	rs12292911	11	11p11.2	A	G	1.25E-06	6.27E-05	1.511E-09	[*MADD*, *MYBPC3*, *PSMC3*, *RAPSN*, *SLC39A13*, and *SPI1*]	upstream
	rs12972970	19	19q13.32	A	G	0	9.38E-03	0	[*APOC1*, *APOE*, *PVRL2*, and *TOMM40*]	intron
	rs1633096	6	6p22.1	T	G	2.02E-03	3.49E-09	3.32E-09	[*HCG4*, *HCG4B*, *HCG8*, *HCG9*, *HLA-A*, *HLA-F-AS1*, *HLA-G*, *HLA-H*, *HLA-J*, *IFITM4P*, *LOC554223*, *PPP1R11*, *RNF39*, *ZNRD1*, and *ZNRD1-AS1*]	downstream
	rs1979377	19	19q13.32	C	A	4.56E-10	4.59E-03	9.751E-11	[*BCL3* and *MIR8085*]	intron
	rs2310752	1	1p31.3	A	G	1.37E-04	5.35E-07	3.68E-09	[*PDE4B*]	intron
	rs3121427	10	10q23.33	G	A	7.19E-05	4.76E-06	1.024E-08	[*MARK2P9*]	intergenic
	rs359539	3	3q25.31	A	G	1.15E-07	1.04E-03	3.182E-09	[*PLCH1*]	intron
	rs4727449	7	7q21.1	T	C	3.29E-08	2.59E-03	3.187E-09	[*AP4M1*, *C7orf43*, *C7orf61*, *CNPY4*, *COPS6*, *GAL3ST4*, *GATS*, *GPC2*, *LAMTOR4*, *MBLAC1*, *MCM7*, *MEPCE*, *MIR25*, *MIR93*, *MIR106B*, *MIR4658*, *MIR6840*, *NYAP1*, *PILRA*, *PILRB*, *PMS2P1*, *PPP1R35*, *PVRIG*, *PVRIG2P*, *SPDYE3*, *STAG3*, *STAG3L5P*, *STAG3L5P-PVRIG2P-PILRB*, *TAF6*, *TSC22D4*, *ZCWPW1*, *ZNF3*, and *ZSCAN21*]	intron
	rs56249331	1	1p35.2	A	G	1.02E-03	1.38E-07	8.476E-09	[*PUM1*, *SNORD85*, *SNORD103A*, and *SNORD103B*]	intergenic
	rs858502	7	7q21.1	T	C	2.36E-09	4.43E-03	3.684E-10	[*GATS*, *PVRIG*, and *STAG3*]	intron
AD_Snoring	rs1004173	6	6p12.3	T	C	5.64E-09	5.92E-03	8.448E-10	[*CD2AP*]	upstream
	rs11100203	4	4q32.1	G	A	4.13E-06	2.93E-05	2.563E-09	[*C4orf45*]	intron
	rs11642303	16	16p11.2	A	C	7.71E-06	2.04E-09	5.513E-13	[*BCKDK*, *BCL7C*, *CTF1*, *FBXL19*, *FBXL19-AS1*, *HSD3B7*, *KAT8*, *LOC101928736*, *MIR762*, *MIR4519*, *ORAI3*, *PRSS8*, *PRSS36*, *PRSS53*, *SETD1A*, *STX1B*, *STX4*, *VKORC1*, *ZNF646*, and *ZNF668*]	intergenic
	rs147188206	19	19q13.32	C	T	2.62E-14	2.89E-03	8.251E-15	[*CLASRP*]	intron
	rs204911	19	19q13.32	A	T	6.58E-10	6.66E-03	1.757E-10	[*CLPTM1*]	intron
	rs28469095	19	19q13.32	C	T	1.07E-38	3.14E-03	4.176E-39	[*GEMIN7*, *NKPD1*, and *PPP1R37*]	missense
	rs3098882	8	8q13.3	T	C	7.02E-03	2.27E-09	7.917E-09	[*RP11-326E22.1*]	intergenic
	rs429358	19	19q13.32	C	T	0	7.73E-03	0	[*APOC1* and *APOE*]	missense
	rs439401	19	19q13.32	T	C	7.70E-167	2.21E-03	1.862E-172	[*APOC1*]	upstream
	rs61597598	2	2q24.1	A	G	6.00E-03	4.52E-14	8.298E-14	[*AC093375.1*]	intron
	rs62402786	6	6p22.3	G	C	2.89E-07	1.67E-03	7.991E-09	[*PRL*]	intron
	rs7514002	1	1p31.3	A	G	8.02E-04	1.43E-07	8.921E-09	[*PATJ*]	intron

Note: **P1** is the Alzheimer’s disease single-trait *p* value, **P2** is the sleep-related phenotype (insomnia, sleep duration, and snoring) single-trait *p* value, and ***P*meta** is the cross-trait meta-analysis *p* value. A1, effect allele; A2, non-effect allele; Chr, chromosome; AD, Alzheimer’s disease; genes in blue are the nearest genes to this locus.

We identified 7, 12, and 12 independent loci shared between AD and insomnia, sleep duration, and snoring, respectively (Table 2). Notably, we identified one novel locus (10q23.33, index SNP: rs3121427, mapped gene: *MARK2P9*, ***P***_meta_ = 1.02 × 10^–8^) shared between AD and sleep duration and three novel loci (4q32.1, index SNP: rs11100203, mapped gene: *C4orf45*, ***P***_meta_ = 2.56 × 10^–9^; 6p22.3, index SNP: rs62402786, mapped gene: *PRL*, ***P***_meta_ = 7.99 × 10^–9^; and 1p31.3, index SNP: rs7514002, mapped gene: *PATJ*, ***P***_meta_ = 8.92 × 10^–9^) shared between AD and snoring. *MARK2P9* (microtubule affinity regulating kinase 2 pseudogene 9) is a pseudogene, and its function is unknown. However, this gene is adjacent to the *IDE* gene, which encodes a zinc metallopeptidase, an enzyme whose preferential affinity for insulin results in the inhibition of beta-amyloid degradation by insulin. The defective function of the *IDE* gene is therefore associated with AD and type 2 diabetes (Qiu and Folstein, 2006; Tang, 2016). *C4orf45* (chromosome 4 open reading frame 45), an uncharacterized protein-coding gene, has been shown to be associated with self-reported educational attainment and cognitive function (Lee et al., 2018). *PRL* (prolactin) encodes the anterior pituitary hormone prolactin and is involved in the regulation of many signaling pathways, including amyloid fibril formation, prolactin signaling, growth hormone receptor signaling, cytokine signaling in the immune system, and protein metabolism pathways (Bole-Feysot et al., 1998; Jacob et al., 2016). Prolactin, a pleiotropic hormone, has many functions in the brain, such as maternal behavior, neurogenesis, and neuronal plasticity, among others (Molina-Salinas et al., 2021). Recently, it has been reported to have a significant role in neuroprotection against excitotoxicity (Molina-Salinas et al., 2021). *PATJ* encodes a protein with multiple PDZ domains and plays a role in cell junction organization, tight junction, and the hippo signaling pathway. The missense variant in *PATJ* has a stronger association with daytime napping than any previously studied sleep-related phenotype (Lane et al., 2017). Research has found co-localization of the daytime napping loci with daytime sleepiness, snoring, BMI, and chronotype at *PATJ*, suggesting an obesity-hypersomnolence pathway (Panossian and Veasey, 2012).

A specific region at 19q13.32 is the strongest shared signal between AD and insomnia (index.SNP: rs6857, ***P***_meta_ = 0), sleep duration (index.SNP: rs12972970, ***P***_meta_ = 0), and snoring (index.SNP: rs429358, ***P***_meta_ = 0). These three loci map to the *AOPE* and *APOC1* genes, which are well known to be major genetic risk factors for AD (Tycko et al., 2004; Lucatelli et al., 2011; Liu et al., 2013). In addition, ten loci shared between AD and SRPs were also mapped to the 19q13.32 region. Excluding this strongest signal region, there are some additional loci of interest.

Index SNP rs2249152 (19p13.3, ***P***_meta_ = 9.53 × 10^–9^, mapped gene: *KDM4B*) was shared between AD and insomnia. *KDM4B* (lysine demethylase 4B) is a protein-coding gene and is engaged in chromatin organization, chromatin-modifying enzymes, and DNA double strand break response. Studies have shown that the *KDM4B* overexpression leads to inflammation and intellectual disability (Taniguchi and Moore, 2014; Zhang et al., 2021). Index SNPs rs12292911 (11p11.2, ***P***_meta_ = 1.51 × 10^–9^, mapped gene: *PSMC3*), rs2310752 (1p31.3, ***P***_meta_ = 3.68 × 10^–9^, mapped gene: *PDE4B*), rs359539 (3q25.31, ***P***_meta_ = 3.18 × 10^–9^, mapped gene: *PLCH1*), and rs56249331 (1p35.2, ***P***_meta_ = 8.48 × 10^–9^, mapped gene: *PUM1*) were shared between AD and sleep duration. *PSMC3* (proteasome 26S subunit, ATPase 3) encodes one of the ATPase subunits, a member of the triple-A family of ATPases that has chaperone-like activity and is associated with AD (Novikova et al., 2021). *PDE4B* encodes a protein that specifically hydrolyzes cAMP; the altered activity of this protein has been associated with schizophrenia and bipolar disorder (Millar et al., 2007; Fatemi et al., 2008). It has also recently been found to modulate cognition, as reduction in the *PDE4B* activity improves memory and long-term plasticity in mouse models, possibly supporting further therapeutic applications (Richter et al., 2013). Findings suggest that brief sleep deprivation disrupts the hippocampal function by increasing the PDE4 activity that interferes with cAMP signaling (Vecsey et al., 2009). Therefore, drugs that enhance cAMP signaling may provide a new therapeutic approach to counteract the cognitive effects of sleep deprivation (Vecsey et al., 2009). *PLCH1* encodes phospholipase C- η enzymes which have recently been implicated in the modulation and amplification of Ca^2^⁺ signals and are known to be expressed in neuronal regions of the brain associated with cognition and memory (Popovics and Stewart, 2012). *PUM1* (pumilio homolog 1) encodes a member of the PUF family and may be involved in translational regulation of embryogenesis and cell development and differentiation. A study has demonstrated the importance of *PUM1* for human neurological development and function and has described its role in neurodegenerative and neurodevelopmental disorders (Gennarino et al., 2018). In addition, we observed other common loci shared between AD and insomnia, sleep duration, or snoring. These results were in line with previous studies (Dashti et al., 2019; Kunkle et al., 2019; Philip R. Jansen et al., 2019; Campos et al., 2020).

### Tissue-Specific Enrichment Analysis

**FIGURE 3:**
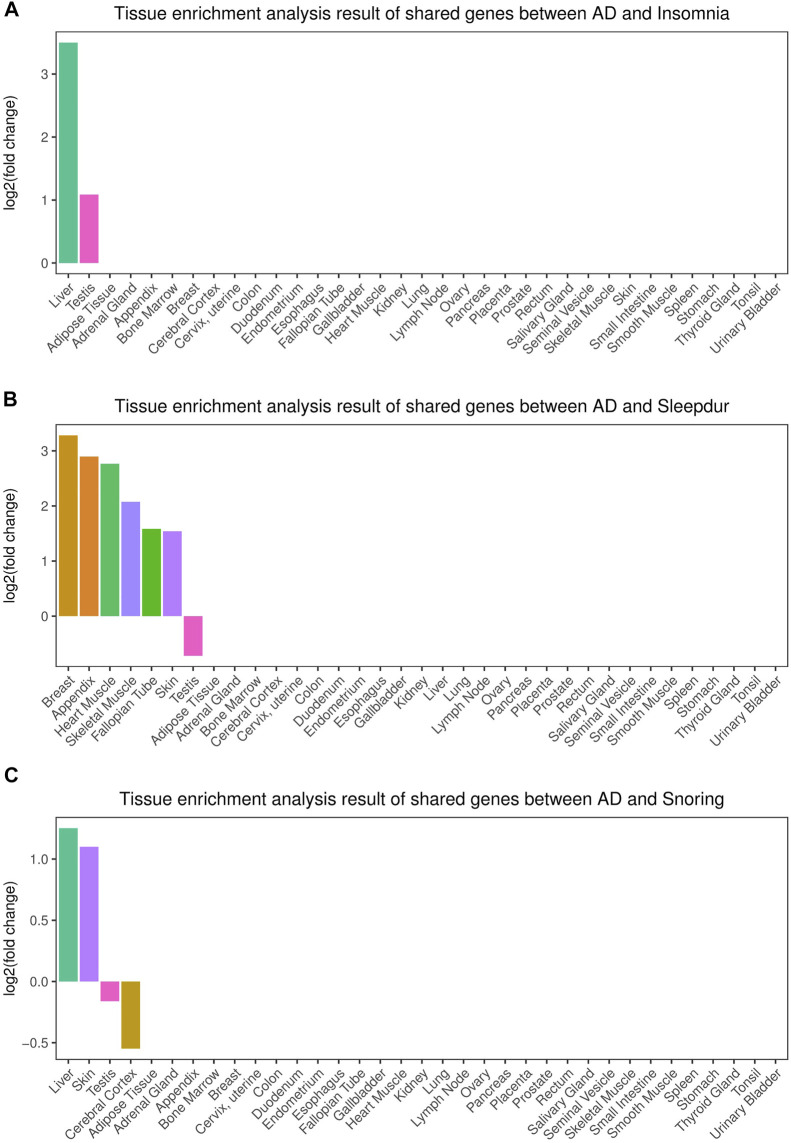
Tissue enrichment for the consensus set. **(A)** Tissue enrichment analysis results of shared genes between AD and insomnia; **(B)** Tissue enrichment analysis results of shared genes between AD and sleep duration. **(C)** Tissue enrichment analysis results of shared genes between AD and snoring. The vertical axis illustrates the logarithm of fold change after the Benjamini–Hochberg correction. The horizontal axis illustrates 35 independent tissue types.

We identified two, six, and two independent tissues that demonstrated significantly enriched expressions of cross-trait–associated genes shared between AD and insomnia, sleep duration, and snoring, respectively (Figure 3). The main strongly enriched tissues were part of the endocrine system, digestive system, integumentary system, and musculoskeletal system (including liver, testis, breast, skin, appendix, skeletal muscle, and heart muscle tissues).

### Functional Enrichment Analysis

**FIGURE 4:**
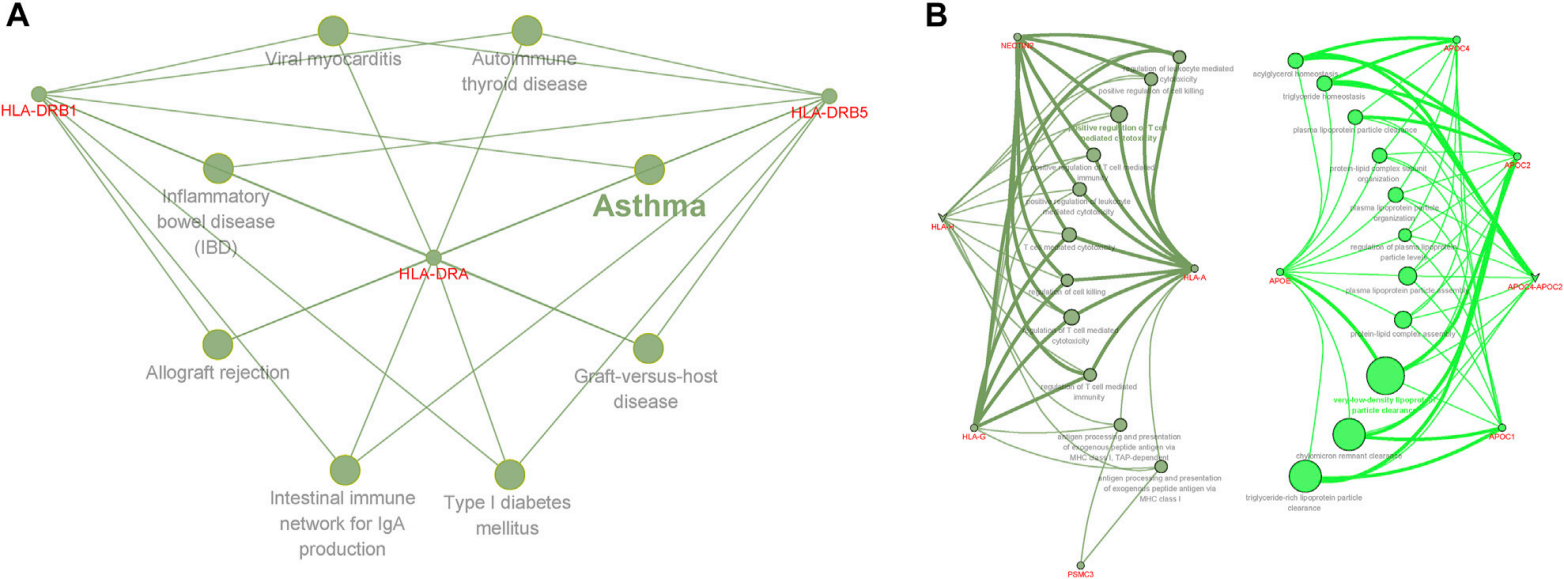
Functional enrichment for the consensus set. Functional enrichment analysis results using the ClueGO method in Cytoscape (Bindea et al., 2009). **(A)** Functional enrichment analysis results of AD and insomnia in KEGG pathways. **(B)** Functional enrichment analysis results for AD and sleep duration in GO terms. Each dot represented a gene, a GO term, or a KEGG pathway. Dots of the same color were considered to be from the same functional group by ClueGO annotation. Gene names were highlighted in red. Each edge indicated the gene was a component gene of the linked GO term or KEGG pathway.

Our results showed that genes shared between AD and insomnia in the KEGG pathways were significantly enriched in immunological disorders such as asthma (adjusted ***p*** = 1.45 × 10^–5^), inflammatory bowel disease (adjusted ***p*** = 1.74 × 10^–5^), allograft rejection (adjusted ***p*** = 2.37 × 10^–5^), and type I diabetes (adjusted ***p*** = 2.47 × 10^–5^), indicating the role in pathways related to immune regulation (Figure 4). Additionally, in GO terms, the genes shared between AD and sleep duration were enriched in very-low-density lipoprotein particle clearance (adjusted ***p*** = 4.66 × 10^–10^), triglyceride-rich lipoprotein particle clearance (adjusted ***p*** = 1.53 × 10^–7^), chylomicron remnant clearance (adjusted ***p*** = 1.53 × 10^–7^), and positive regulation of T-cell–mediated cytotoxicity (adjusted ***p*** = 1.91 × 10^–5^), implicating that processes involving immunity and lipid metabolism may account for shared causes. Evidence of ClueGO log and enrichment results are provided in Supplementary Tables S10–S11 (Supplementary Results).

### Potential Drug Target Genes and Protein–Protein Interaction Analysis

**FIGURE 5:**
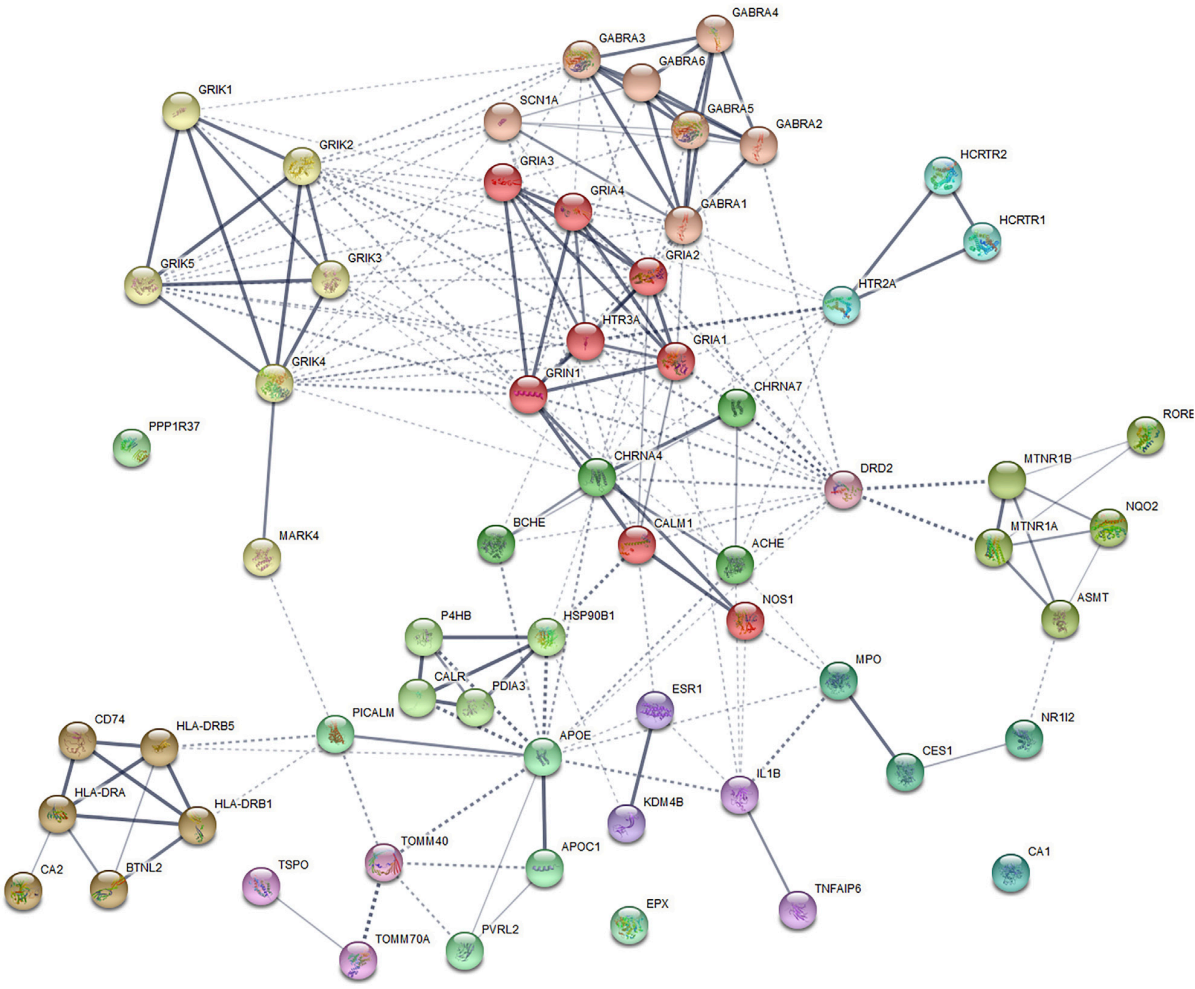
Protein–protein interaction subnetworks identified by the Markov cluster algorithm. The protein-coding genes shared between AD and insomnia and the FDA-approved AD and insomnia drug target genes were used to construct a functional similarity network of genes (see the *Methods* section). Nodes are colored to show association. The thickness of lines connecting nodes indicates the strength of the association between nodes.

Disease-related genes are natural candidates for drug development in complex diseases (Zhao et al., 2015; Lee et al., 2016). We further compared shared genes between AD and insomnia identified from CPASSOC with known target genes of AD and insomnia meditation using the TTD. Overall, eight FDA-approved drugs were found for AD and corresponded to 11 target genes. For insomnia, 28 FDA-approved drugs were found to correspond to 36 target genes (Supplementary Table S12). None of them were included in the shared genes we identified. We queried the STRING database for the interactions between the 47 drug target genes and the 15 shared genes (Figure 5). We observed three shared genes had medium confidence (>0.40) in interaction with nine drug target genes: *APOE* (shared gene) interacting with *ACHE*, *BCHE*, *ESR1*, *IL1B*, *MPO*, *DRD2*, and *CHRNA4* (drug targets), *MARK4* (shared gene) interacting with *GRIK4* (drug target), and *HLA-DRA* (shared gene) interacting with *CA2* (drug target). These gene interactions provide some insights into potential target genes for identifying drug targets from multi-omics datasets.

### Causal Inference

We then used bi-directional MR analysis to test the causality between AD and SRPs. Forward MR showed non-significant instrumental effects of AD on three SRPs (Table 3), while reversed MR showed there was robust evidence suggesting that per-SD increase in genetic liability to insomnia was associated with a higher risk of AD (OR_IVW_ = 1.02, ***P***_IVW_ = 6.7 × 10^–6^, Table 3). However, after adjusting for sleep duration and MDD, MVMR showed no significant direct effect of genetic liability to insomnia on AD (OR_MVMR-IVW_ = 1.03; *p* = 0.052, Table 4), implying a potential mediating role of sleep duration and MDD in the association of insomnia with AD. Direct effects are slightly different from total effects but have directional consistency. In our analyses, all heterogeneity *p*-values were non-significant (***P***_heterogeneity_ > 0.01), indicating at worst only subtle heterogeneity among retained instruments (Supplementary Table S13). Sensitivity analysis for the main MR analysis using weighted median, simple median, MR-RAPS, MR-Egger, and MR-PRESSO suggested there was no systematic bias due to pleiotropy (all ***P***_MR_Egger_ > 0.05) (Supplementary Table S13). MR-Steiger results showed that all the causal estimates were oriented in the intended direction (all ***P***_MR-Steiger_ < 0.05). Taken together, the instrumental analysis suggested a potential causal role of the increased risk of insomnia on a higher risk of AD.

**TABLE 3:** Causal inference between Alzheimer’s disease and sleep-related phenotypes using two-sample MR.

Outcome	Direction	Method	N_snp	Causal_Effect_Size^a^	SE	*p*_value
Insomnia	Forward	Simple median	24	−0.048	0.076	5.24E-01
	Forward	Weighted median	24	−0.022	0.073	7.59E-01
	Forward	MR-Egger	24	−0.122	0.091	1.91E-01
	Forward	Inverse-variance weighted	24	−0.029	0.058	6.20E-01
	Forward	MR-RAPS	24	−0.036	0.055	5.10E-01
	Forward	MR-PRESSO	24	−0.029	0.058	6.25E-01
	Reverse	Simple median	201	0.023	0.006	**1.18E-04**
	Reverse	Weighted median	201	0.017	0.006	**7.54E-03**
	Reverse	MR-Egger	201	−0.02	0.018	2.65E-01
	Reverse	Inverse-variance weighted	201	0.021	0.005	**6.70E-06**
	Reverse	MR-RAPS	201	0.021	0.005	**1.20E-05**
	Reverse	MR-PRESSO	201	0.021	0.005	**1.14E-05**
Sleepdur	Forward	Simple median	24	−0.035	0.039	3.69E-01
	Forward	Weighted median	24	−0.067	0.033	4.08E-02
	Forward	MR-Egger	24	−0.088	0.038	2.78E-02
	Forward	Inverse-variance weighted	24	−0.049	0.024	3.67E-02
	Forward	MR-RAPS	24	−0.05	0.024	3.83E-02
	Forward	MR-PRESSO	24	−0.049	0.018	1.04E-02
	Reverse	Simple median	43	−0.031	0.034	3.66E-01
	Reverse	Weighted median	43	−0.021	0.033	5.24E-01
	Reverse	MR-Egger	43	−0.115	0.066	8.78E-02
	Reverse	Inverse-variance weighted	43	−0.049	0.023	3.58E-02
	Reverse	MR-RAPS	43	−0.052	0.024	2.88E-02
	Reverse	MR-PRESSO	43	−0.049	0.023	4.18E-02
Snoring	Forward	Simple median	24	0.101	0.084	2.28E-01
	Forward	Weighted median	24	−0.029	0.073	6.86E-01
	Forward	MR-Egger	24	−0.156	0.115	1.89E-01
	Forward	Inverse-variance weighted	24	−0.049	0.073	5.01E-01
	Forward	MR-RAPS	24	−0.089	0.065	1.70E-01
	Forward	MR-PRESSO	24	−0.049	0.073	5.08E-01
	Reverse	Simple median	32	0.032	0.018	8.30E-02
	Reverse	Weighted median	32	0.031	0.018	7.59E-02
	Reverse	MR-Egger	32	−0.068	0.093	4.71E-01
	Reverse	Inverse-variance weighted	32	0.029	0.014	4.60E-02
	Reverse	MR-RAPS	32	0.029	0.014	4.40E-02
	Reverse	MR-PRESSO	32	0.029	0.014	5.48E-02

Sleepdur, sleep duration; MR, Mendelian randomization; Direction, forward means the causal effect size of Alzheimer’s disease on sleep-related phenotypes, and reverse means the causal effect size of sleep-related phenotypes on Alzheimer’s disease; N_snp, number of instrumental variables; The threshold of significance was set at the Bonferroni-adjusted level of *p*-value < 0.016 (0.05/3). Abbreviations as in Table1.

a The causal effect size was the beta coefficient from linear or logistic regression models for the corresponding outcome.

**TABLE 4:** Multivariable Mendelian randomization of insomnia and Alzheimer’s disease adjusted for sleep duration and major depression.

Exposure	Outcome	BETA	SE	*p*	F-stat	Q-stat for instrument validity	*p*-value for instrument validity
Insomnia	AD	0.025	0.013	0.052	4.4	344	5.72E-05
Sleepdur		−0.030	0.025	0.239	4.6		
MDD		0.017	0.009	0.078	4.6		

Note: Sleepdur: sleep duration; MDD: major depression; SE = standard error; Q-stat: Cochran’s Q statistic; F-stat: conditional F-statistic.

### Single-Trait TWAS and Shared Genetics Between AD and SRPs From the TWAS

We next went down to the gene level to examine the shared TWAS genes between AD and related SRPs. In total, 2108 gene-tissue pairs were found across 48 GTEx tissues to be significantly associated with AD after BH correction, in addition to 551, 2227, and 3313 gene-tissue pairs with insomnia, sleep duration, and snoring, respectively (Supplementary Figure S4).

**FIGURE 6:**
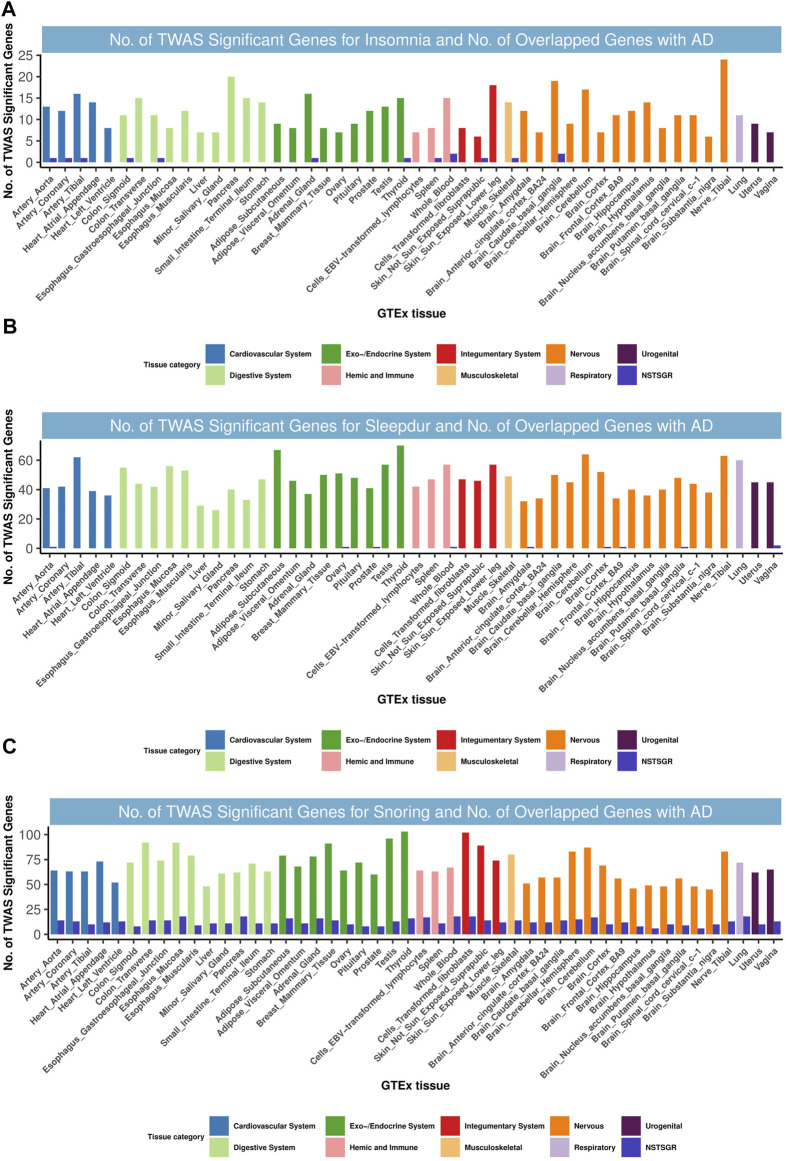
Numbers of significant genes related with insomnia, sleepdur, and snoring and the number of shared genes with AD. Significant genes were identified by ***P***_BH_ < 0.05. GTEx, genotype-tissue expression project; GWAS, genome-wide association studies; TWAS, transcriptome-wide association study; NSTSGR, number of shared TWAS-significant genes with AD; AD, Alzheimer’s disease; Sleepdur, sleep duration. **(A)**: No. of TWAS-significant genes for insomnia and No. of overlapped genes with AD. **(B)**: No. of TWAS-significant genes for Sleepdur and No. of overlapped genes with AD. **(C)**: No. of TWAS-significant genes for snoring and No. of overlapped genes with AD

We identified nine, eight, and 77 TWAS-significant genes shared between AD and insomnia, sleep duration, and snoring, respectively (Supplementary Tables S14–S16), most of which were observed in tissues from the immune system, cardiovascular system, endocrine system, digestive system, and nervous system (Figure 6). Restricting this list to shared genes with independent signals, we identified 30 genes that were TWAS-significant for both AD and at least one of the SRP traits from tissues including the liver, brain, thyroid, skin, heart, and muscle (Supplementary Tables S14–S16). Intriguingly and highly consistent with the results from CPASSOC, some loci were shared among AD and related SRPs. For example, the *PTPMT1* gene, located at 11p11.2, was co-independently TWAS-significant for AD, insomnia, and sleep duration and was simultaneously a prominent shared locus among AD and sleep duration. *PTPMT1* is a lipid phosphatase that dephosphorylates mitochondrial proteins, which in turn regulates mitochondrial membrane integrity. Its expression is highly correlated in human brains, and this correlation is lost in AD brains (Efthymiou and Goate, 2017). *PTPMT1* was previously thought to have a role in the rhythmicity of the sleep cycle (Nohara et al., 2020). Notably, we found *KAT8* (16p11.2) was co-significant for AD and snoring and was also the most enriched and significant gene in 48 tissues. *KAT8* (lysine acetyltransferase 8) encodes a member of the MYST histone acetylase protein family. A study indicated that aberrant expression patterns of *KAT8* might be associated with AD progression (Chen et al., 2020).

## Discussion

Our study has five main findings. First and foremost, we provided evidence that AD shared a genetic basis with insomnia, snoring, and sleep duration. Second, cross-trait meta-analysis identified independent shared loci between AD and insomnia, snoring, or sleep duration, and functional analysis highlighted that those shared loci were mainly enriched in the liver tissue and lipid metabolic system, as well as the immune inflammatory system, and were involved in immunological disorders, very-low-density lipoprotein particle clearance, triglyceride-rich lipoprotein particle clearance, chylomicron remnant clearance, and positive regulation of T-cell–mediated cytotoxicity pathways. Third, PPI analysis identified three potential drug target genes that interact with known FDA-approved drug target genes. Fourth, TWAS identified genes that were shared between AD and sleep phenotypes in tissue from the immune system, cardiovascular system, endocrine system, digestive system, and nervous system. Fifth, bi-directional MR suggested that a higher risk of insomnia was causally related to a higher risk of AD. Our findings advance our understanding of the genetic contribution of AD and sleep patterns, provide insights into the potential regulatory role of shared inheritance whose function warrants follow-up, and elucidate the etiology and mechanisms underlying the co-morbidity of AD and sleep disorders.

Circadian rhythm disturbances have been suggested as biomarkers for clinical stage AD (Liguori et al., 2020). The findings of our genetic analyses were highly consistent, generally supporting the observational positive associations between AD with insomnia (Sadeghmousavi et al., 2020) and snoring (Kuo et al., 2020) and the negative associations with sleep duration (Lutsey et al., 2018). We also observed that AD was positively associated with napping (*r*_g_ = 0.16, *p* = 1.61 × 10^–2^), but this significance disappeared after Bonferroni correction. However, recent longitudinal studies have shown that men with longer napping duration had greater cognitive decline and a higher risk of cognitive impairment after adjustment for all covariates (Leng et al., 2019). Mechanisms for this association between AD and napping were unknown; it might be partially explained by daytime napping which is a result of the erosion of the area of the brain responsible for wakefulness by toxic tau proteins, the accumulation of which ultimately leads to AD; however, such a putative causal mechanism needs further experimental validation (Leng et al., 2019).

Meanwhile, 31 independent SNPs from CPASSOC and 30 genes from independent TWAS signals of both AD and three SRPs suggested potential functions relevant to AD. The loci identified in both the CPASSOC and TWAS analysis revealed potential shared biological mechanisms in AD progress and SRP regulation involving immunological disorders, very-low-density lipoprotein particle clearance, triglyceride-rich lipoprotein particle clearance, chylomicron remnant clearance, and positive regulation of T-cell–mediated cytotoxicity pathways. Consequently, we highlighted the potentially interesting functions of the novel associations for *PRL* (6p22.3) between AD and snoring, as well as the focused *PTPMT1*(11p11.2) and *KAT8*(16p11.2) regions shared between AD and insomnia or sleep duration.

Shared genes associated with AD and SRPs were enriched for expressions in most liver and brain tissues, indicating that these disorders might be caused by malfunctions of the endocrine system and nervous system. For example, *PRL* can influence the sleep structure, and *PRL*-deficient mice display less rapid eye movement (REM) sleep than wild-type mice (Machado et al., 2017). Molina-Salinas et al. have shown that *PRL* can inhibit glutamate excitotoxicity through the AKT and STAT5 pathways, thereby protecting neuronal cells and decreasing the progression of Alzheimer’s disease (Molina-Salinas et al., 2021). Evidence has suggested that the somatostatin expression is downregulated in early aging brains in snoring samples, leading to a progressive decrease in *PRL* and neprilysin activity and resulting in amyloid b (Ab) peptide accumulation in AD patients (Cao et al., 2021). Additionally, *PTPMT1* is localized to mitochondria *via* an N-terminal signaling sequence and is found anchored to the stromal surface of the inner membrane. The study shows that activation of protein tyrosine phosphatase (PTP) hastened the progression of AD (Stewart and Chen, 2020). W. Lutz et al. identified *PTPMT1* as a common signal in AD and a major depressive disorder, which showed a consistent moderate expression in brain tissues (Lutz et al., 2020). A highly promising candidate gene is *KAT8*, as the dominant SNP at 16p11.2 is located within the third intron of *KAT8*, and multiple important variants within this locus affect the expression or methylation levels of *KAT8* in multiple brain regions, including the hippocampus (Li et al., 2020). The chromatin modifier *KAT8* is regulated by *KANSL1*, a gene associated with AD deficient in Apoε4. A study on Parkinson’s disease reported that *KAT8* is a potentially causal gene based on GWAS and differential gene expression, implying that *KAT8* may have a common role in the neurodegeneration of AD and Parkinson’s disease (Dumitriu et al., 2016). Although previously reported information on gene function may be of great value, it is best to consider all implicated genes as putative causal factors to guide potential functional follow-up experiments.

Our MR analysis does not support a causal effect of sleep duration on AD risk. Notably, growing evidence suggests a J-shaped association between sleep duration and AD, suggesting that the causal effect in the long-sleeper group was larger than in the short-sleeper group (Henry et al., 2019; Leng et al., 2021). Apparent inconsistencies between our findings and previous MR studies may be partly due to different definitions, diagnostic criteria, and forms of characterization of AD (such as cognitive impairment, memory loss, reaction time, and so on) (Henry et al., 2019), different types of data (individual-level data or summary-level data) (Henry et al., 2019), different MR methods (linear MR or non-linear MR) (Henry et al., 2019), or different statistical analysis methods (genetic risk score) (Leng et al., 2021). In addition, MR provided strong evidence that insomnia is associated with a higher risk of AD. The pathogenic processes through which insomnia increases the likelihood of developing AD may entail abnormal Aβ deposition or an imbalance in the neurotransmitter regulating system. Neurons in the physiological state release Aβ, and Aβ levels in the brain fluctuate from day to day: secretion increases during wakefulness and decreases during sleep; a decrease in low-quality sleep or slow-wave sleep increases the cortical neuronal activity and also increases the release of Aβ compared to high-quality sleep (Ju et al., 2014). Studies have found that having adequate sleep is beneficial in eliminating Aβ_42_ levels in the cerebrospinal fluid, while insomnia causes impaired clearance (Ooms et al., 2014). Furthermore, certain excitatory or inhibitory neurotransmitters have been shown in studies to be involved in the regulation of sleep and wakefulness, promoting neuroplasticity and memory formation, and these transmitters play an important role in individual learning and memory consolidation (Leiser et al., 2015; Atucha et al., 2017; Niwa et al., 2018), while insomnia will affect the balance of the transmitters and affect the brain’s memory function in the long run. However, we found no causal relationship between AD and insomnia, whereas a recent MR study conducted by Huang et al. found that a higher risk of AD was associated with a lower risk of insomnia (OR: 0.99, *P*_IVW_ = 7 × 10^–13^) (Huang et al., 2020). Given the consistency in population, sample size, and statistical methods between the study by Huang et al. and the present study, we consider that the difference in results is due to the fact that Huang et al. adopted F-statistic > 10 to filter exposure-related SNPs to reduce the weak instrumental bias of using genotype data (Huang et al., 2020). Finally, there was little evidence to support a causal effect of sleep duration and snoring on AD risk; this finding is consistent with recent research studies (Huang et al., 2020; Anderson et al., 2021). Our findings add to previous evidence that insomnia pathologically leads to elevated Aβ levels in the cerebrospinal fluid and induces aggregation of Aβ peptides and tau proteins (Di Meco et al., 2014; Chen et al., 2018). There are also suggestive results reinforcing the evidence that AD pathology leads to increased wakefulness and high sleep fragmentation in transgenic mouse models (Roh et al., 2012) and results in neuronal loss of the suprachiasmatic nucleus (SCN), the master circadian clock of mammals, and the locus coeruleus, which are essential for maintaining normal wakefulness (Wang et al., 2015). Mechanisms underlying the causality between SRPs and AD remain to be elucidated.

Our study has notable strengths. Specifically, we used data from the largest GWAS available for each trait or disorder, and we explored a wide range of SPRs. Second, we leveraged SUPERGNOVA and GNOVA to assess the local genetic correlation and annotation-specific genetic correlation between AD and seven SRPs, respectively. SUPERGNOVA has stronger statistical performance than HESS, and GNOVA provides more accurate genetic covariance estimates and powerful statistical inference than LDSC. Third, the identification of potential target genes through PPI analysis provides a new perspective on the shared structure. Fourth, we conducted cross-trait meta-analysis using CPASSOC, which is robust to heterogeneous effects and overlaps samples between two phenotypes. Nevertheless, there are several potential limitations to our study. First, although TWAS increased the power to detect significant expression trait associations, the relatively smaller sample size for metabolic traits and GTEx reference panels in certain tissues may be inadequate to detect signals with small to moderate effects. Second, despite the large sample sizes of the consortium-based meta-analysis studies, there were differences in the sample size and number of SNPs among different studies. Therefore, the enrichment of SNPs with potential common effects may be lower for traits with relatively few loci and samples in the source studies. Third, some of the observed associations may not be due to independent effects of the same locus on AD and SRPs but rather due to correlations of traits in the causal pathway or through other unmeasured traits. Fourth, our study was limited to European ancestry, and the shared genetics in other ethnic groups are uncertain. Therefore, future research in other ethnic groups is encouraged. More work is needed to identify individual cell types and more detailed molecular mechanisms with the goal of developing potential therapeutic strategies.

In conclusion, our study provides strong evidence of genetic correlations and causality between AD and sleep patterns and identifies genetic loci associated with both AD and SRP risk, thus providing therapeutic opportunities to improve sleep quality and lower the risk of AD. Our results further advance our understanding of AD and provide insight into the shared etiology of comorbid AD and sleep disorders.

## Data Availability

The datasets presented in this study can be found in online repositories. The names of the repository/repositories and accession number(s) can be found at: https://ctg.cncr.nl/software/summary_statistics.

## Abbreviations

SRPs, sleep-related phenotypes; HDL, high-definition likelihood; LDSC, linkage disequilibrium score regression; GNOVA, genetic covariance analyzer; SUPERGNOVA, super genetic covariance analyzer; CPASSOC, cross phenotype association study; TSEA, tissue-specific enrichment analysis; MR, Mendelian randomization; TWAS, transcriptome-wide association study; SNP, single nucleotide polymorphism; GTEx, genotype-tissue expression portal.
