# Supplementary material for: Sleep and Late-Onset Alzheimer’s Disease: Shared Genetic Risk Factors, Drug Targets, Molecular Mechanisms, and Causal Effects
Source: Front Genet. 2022 May 17;13:794202. doi: 10.3389/fgene.2022.794202 (PMC9152224; doi:10.3389/fgene.2022.794202)

**Sleep and** **Alzheimer’s disease: Shared genetic risk factors,** **drug targets, molecular mechanisms, and causal effects**

Dongze Chen^1^, Xinpei Wang^1^, Jinzhu Jia^1,2^ and Tao Huang^3,4,5,6^

**^1^**Department of Biostatistics, School of Public Health, Peking University, Beijing, China

**^2^**Center for Statistical Science, Peking University, Beijing, China

**^3^**Department of Epidemiology & Biostatistics, School of Public Health, Peking University, Beijing, China

^4^Department of Global Health, School of Public Health, Peking University, 100191 China.

^5^Key Laboratory of Molecular Cardiovascular Sciences (Peking University), Ministry of Education, 100191 China.

^6^Center for Intelligent Public Health, Institute for Artificial Intelligence, Peking University, Beijing, China.

**Contents**

[Online Methods 4](#_Toc78451348)

[Summary statistics for Alzheimer’s disease and sleep-related phenotypes, quality control 4](#_Toc78451349)

[Study design 4](#_Toc78451350)

[Cross trait meta-analysis 4](#_Toc78451351)

[Online Results 5](#_Toc78451352)

[The ClueGO log in our experiment 5](#_Toc78451353)

[References 8](#_Toc78451354)

[Supplementary Table 9](#_Toc78451355)

[Supplementary Table1 Information of Summary Statistics Used in This Study and Trait Definition 9](#_Toc78451356)

[Supplementary Table2 Definition of self-reported sleep-related phenotypes 10](#_Toc78451357)

[Supplementary Table3 The 20 functional categories used in partitional genetic correlation 12](#_Toc78451358)

[Supplementary Table4 The 22 autosomes annotations used in partitional genetic correlation 13](#_Toc78451359)

[Supplementary Table5 The ten broadly-defined tissue types annotations and 66 epigenetic annotations used in partitional genetic correlation 14](#_Toc78451360)

[Supplementary Table6 Fine-mapping 99% credible-set of sentinel SNP from cross-trait meta-analysis between Alzheimer's disease (AD) and insomnia. 17](#_Toc78451361)

[Supplementary Table7 Fine-mapping 99% credible-set of sentinel SNP from cross-trait meta-analysis between Alzheimer's disease (AD) and sleep duration (Sleepdur). 25](#_Toc78451362)

[Supplementary Table8 Fine-mapping 99% credible-set of sentinel SNP from cross-trait meta-analysis between Alzheimer's disease (AD) and snoring. 33](#_Toc78451363)

[Supplementary Table9 Co-localization analysis of both Alzheimer’s disease and sleep-related phenotypes are associated and share causal variants 51](#_Toc78451364)

[Supplementary Table10. Biological process of the shared gene set between AD and insomnia in KEGG pathways 53](#_Toc78451365)

[Supplementary Table11. Biological process of the shared gene set between AD and sleep duration in GO terms. 54](#_Toc78451366)

[Supplementary Table12. FDA approved Alzheimer’s Disease drugs and insomnia drugs and their target genes obtained from Therapeutic Target Database and DrugBank 56](#_Toc78451367)

[Supplementary Table13. Horizontal pleiotropy test, steiger directionality test and heterogeneity test in the bidirectional MR analysis between AD and sleep-related phenotypes. 58](#_Toc78451368)

[Supplementary Table 14 Shared TWAS significant genes between AD and Insomnia across 48 GTEx tissues. 59](#_Toc78451369)

[Supplementary Table 15 Shared TWAS significant genes between AD and Sleepdur across 48 GTEx tissues. 60](#_Toc78451370)

[Supplementary Table 16 Shared TWAS significant genes between AD and Snoring across 48 GTEx tissues. 62](#_Toc78451371)

[Supplementary Figure 89](#_Toc78451372)

[Supplementary Figure1. Quantile-quantile (QQ) plot for Alzheimer's disease (AD), Insomnia, and cross-trait meta-analysis between AD and Insomnia. 89](#_Toc78451373)

[Supplementary Figure2. Quantile-quantile (QQ) plot for Alzheimer's disease (AD), Sleep duration (Sleepdur), and cross-trait meta-analysis between AD and Sleepdur. 89](#_Toc78451374)

[Supplementary Figure3. Quantile-quantile (QQ) plot for Alzheimer's disease (AD), Snoring, and cross-trait meta-analysis between AD and Snoring. 90](#_Toc78451375)

[Supplementary Figure4. Number of significant expression-trait associations from transcriptome-wide association study (TWAS) for Alzheimer’s disease, insomnia, sleep duration, and snoring 91](#_Toc78451376)

# Online Methods

## Summary statistics for Alzheimer’s disease and sleep-related phenotypes, quality control

Information for all of the Consortia is presented in eTable1.The ethical approval and quality control procedures of each consortium have been described in previous studies, respectively. In addition, we restricted the chromosome region to autosomal chromosomes and excluded single nucleotide polymorphism (SNPs) in MHC region (chr6:25Mb-34Mb) as recommended. All participants provided written informed consent to each of the sub-cohort of the consortium.

## Study design

We assessed the genetic correlations of AD with seven sleep-related phenotypes, followed by a cross phenotype association study to combine association evidence for AD with related SRPs at individual loci and a bidirectional two sample Mendelian Randomization (MR) analysis to infer the causality between them. Then, we investigated the shared genes between them using TWAS, which relates genetic effect of tissue-specific gene expression with each of the aforementioned traits. We further applied tissue-specific gene enrichment analysis, protein-protein interaction analysis functional enrichment analysis, colocalization analysis to explore possible biological pathways, molecular mechanisms and causal loci.

## Cross trait meta-analysis

After assessing genetic correlations among all traits, we applied cross-trait GWAS meta-analysis using the R package Cross-Phenotype Association (CPASSOC) to combine the association evidence for AD with related SRPs, based on the criteria of both Rg > 10% and P_bonferroni < 0.05 from HDL^1^. SHet is a cross-phenotype meta-analysis method based on fixed effect model. It can be viewed as the maximum of weighted sum of trait-specific test statistics, which is closely related to a gamma distribution. It is more powerful when there is heterogeneous effect present between studies, which is common in meta-analysis of different phenotypes ^2, 3^. SHet also uses the sample size for a trait as a weight instead of variance.

We applied PLINK1.9 clumping function (parameters: --clump-p1 1.67e-8 --clump-p2 1e-5 --clump-r2 0.4 --clump-kb 500) to determine top loci that are independent of each other, i.e., variants with P value less than 1×10^-5^ have *r*^2^ more than 0.4 and less than 500 kb away from the peak will be assigned to that peak’s clump. We identified all genes falling within each clump region. A P value of 1.67×10^-9^ (5×10^-8^/3) was used as genome-wide significance level for cross-trait meta-analysis to account of 3 meta-analysis testing. SNPs with a meta-analysis P value less than 1.67×10^-9^ and trait specific P value less than 1×10^-5^ were selected for downstream analysis.

# Online Results

## The ClueGO log in our experiment

1. Part1. AD and insomnia in KEGG pathways.

### All results were created with ClueGO v2.5.7 ###

Organism analyzed: Homo Sapiens [9606]

Identifier types used: [SymbolID]

Evidence codes used: [All]

#Genes in KEGG_08.05.2020 : 8024

#All unique genes in selected ontologies: 8024 (reference set for hypergeometric test)

#Genes from Cluster#1: unique uploaded ids 15 -> corresponding genes 14, with 1 (6.67%) missing -> 14 recognized by ClueGO.

-> To improve the % of found genes, verify gene identifiers, download new available ClueGO conversion files or add/request additional files.

#Genes with functional annotations in all selected Ontologies from Cluster#1: 7 (50.0%) -> 7 (50.0%) are not functionally annotated in any selected Ontology!

-> To improve the % of annotated genes, chose additional ontologies.

#Genes from all Clusters associated to 8 representative Terms and Pathways (after applying general selection criteria): 3 (21.43%)

KappaScore Grouping:

Iteration: 0 with 1 groups

Final KappaScore groups = 1

# Terms not grouped = 0

# Merge redundant groups with >50.0% overlap

Final group size after merging: 1

#GO All Terms Specific for Cluster #1: 8

Ontology used:

KEGG_08.05.2020

Evidence codes used:

All

Identifiers used:

SymbolID

List of missing Genes:

Cluster #1

Gene IDs not found (no annotation or wrong ids) in Cluster#1:

snoZ6

Statistical Test Used = Enrichment/Depletion (Two-sided hypergeometric test)

Correction Method Used = Bonferroni step down

Min GO Level = 3

Max GO Level = 8

Cluster #1

Sample File Name = File selection: ManuallyAddedOrModifiedIDs

Number of Genes = 3

Min Percentage = 4.0

GO Fusion = false

GO Group = true

Kappa Score Threshold = 0.4

Over View Term = SmallestPValue

Group By Kappa Statistics = true

Initial Group Size = 1

Sharing Group Percentage = 50.0

1. Part1. AD and sleep duration in GO terms.

### All results were created with ClueGO v2.5.7 ###

Organism analyzed: Homo Sapiens [9606]

Identifier types used: [SymbolID]

Evidence codes used: [All]

#Genes in GO_BiologicalProcess-EBI-UniProt-GOA-ACAP-ARAP_08.05.2020_00h00 : 17972

#All unique genes in selected ontologies: 17972 (reference set for hypergeometric test)

#Genes from Cluster#1: unique uploaded ids 72 -> corresponding genes 70, with 1 (1.41%) missing -> 70 recognized by ClueGO.

-> To improve the % of found genes, verify gene identifiers, download new available ClueGO conversion files or add/request additional files.

#Genes with functional annotations in all selected Ontologies from Cluster#1: 47 (67.14%) -> 23 (32.86%) are not functionally annotated in any selected Ontology!

-> To improve the % of annotated genes, chose additional ontologies.

#Genes from all Clusters associated to 22 representative Terms and Pathways (after applying general selection criteria): 10 (14.29%)

KappaScore Grouping:

Iteration: 0 with 2 groups

Final KappaScore groups = 2

# Terms not grouped = 0

# Merge redundant groups with >50.0% overlap

Final group size after merging: 2

#GO All Terms Specific for Cluster #1: 22

Ontology used:

GO_BiologicalProcess-EBI-UniProt-GOA-ACAP-ARAP_08.05.2020_00h00

Evidence codes used:

All

Identifiers used:

SymbolID

List of missing Genes:

Cluster #1

Gene IDs not found (no annotation or wrong ids) in Cluster#1:

LOC554223

Statistical Test Used = Enrichment/Depletion (Two-sided hypergeometric test)

Correction Method Used = Bonferroni step down

Min GO Level = 3

Max GO Level = 8

Cluster #1

Sample File Name = File selection: ManuallyAddedOrModifiedIDs

Number of Genes = 4

Min Percentage = 4.0

GO Fusion = false

GO Group = true

Kappa Score Threshold = 0.4

Over View Term = SmallestPValue

Group By Kappa Statistics = true

Initial Group Size = 1

Sharing Group Percentage = 50.0

# References

1. Ning Z, Pawitan Y, Shen X. High-definition likelihood inference of genetic correlations across human complex traits. *Nature genetics* 2020; **52**(8)**:** 859-864.

2. Watson HJ, Yilmaz Z, Thornton LM, Hübel C, Coleman JRI, Gaspar HA *et al.* Genome-wide association study identifies eight risk loci and implicates metabo-psychiatric origins for anorexia nervosa. *Nature genetics* 2019; **51**(8)**:** 1207-1214.

3. Zhu Z, Anttila V, Smoller JW, Lee PH. Statistical power and utility of meta-analysis methods for cross-phenotype genome-wide association studies. *PLoS One* 2018; **13**(3)**:** e0193256.

# Supplementary Table

## Supplementary Table1 Information of Summary Statistics Used in This Study and Trait Definition

| Trait | Trait types | Consortium | Sample size | Reference |
| --- | --- | --- | --- | --- |
| AD/AD-by-proxy | binary | PGC-ALZ, IGAP, ADSP, UKB | 455258(71880 cases and 383378 controls) | Jansen IE, et al. Genome-wide meta-analysis identifies new loci and functional pathways influencing Alzheimer's disease risk. Nature genetics 51, 404-413 (2019). |
| Insomnia | binary | UKB, 23andMe | 1331010 (397959 cases, 933051 controls) | Jansen PR, et al. Genome-wide analysis of insomnia in 1,331,010 individuals identifies new risk loci and functional pathways. Nature genetics 51, 394-403 (2019). |
| Morningness | continuous |  | 384490 |  |
| Sleep duration | continuous |  | 384317 |  |
| Ease of getting up in the morning | continuous |  | 385949 |  |
| Daytime napping | dichotomous |  | 386577 (20102 napping, 366475 no napping) |  |
| Daytime sleepiness/Dozing | dichotomous |  | 386548 (10050 dozing, 376498 no dozing) |  |
| Snoring | binary |  | 359916 (134248 cases, 225668 controls) |  |

*Note*: AD: Alzheimer’s disease; PGC-ALZ: Alzheimer workgroup initiative of the Psychiatric Genomic Consortium; IGAP: International Genomics of Alzheimer's Project; ADSP: Alzheimer’s Disease Sequencing Project; UKB: UK biobank

## Supplementary Table2 Definition of self-reported sleep-related phenotypes

|  | Trait definition |
| --- | --- |
| Insomnia | Insomnia complaints were assessed by asking: “Do you have trouble falling asleep at night or do you wake up in the middle of the night?” Participants were instructed to answer this question in relation to the previous four weeks when in doubt. The participants were able to choose one of the following four answers: “never/rarely”, “sometimes”, “usually”, or “prefer not to answer”. Insomnia cases were defined as participants who answered this question with “usually”, while participants answering “never/rarely” or “sometimes” were defined as controls. |
| Morningness | Morningness (i.e. being a morning person rather than an evening person) was assessed by asking: “Do you consider yourself to be?”, followed by the answering categories “Definitely a ‘morning’ person”, “More a ‘morning’ than ‘evening’ person”, “More an ‘evening’ than a ‘morning’ person”, “Definitely an ‘evening’ person”, and “Do not know”, and was analyzed on a continuous scale. |
| Sleep duration | Sleep duration was assessed by asking: “About how many hours sleep do you get in every 24 hours? (please include naps)". The answer could only contain integer values (round hours). Sleep duration was analyzed as a continuous outcome |
| Ease of getting up in the morning | Ease of getting up in the morning was assessed by asking: “On an average day, how easy do you find getting up in the morning?”. The possible answers included “not at all easy”, “not very easy”, “fairly easy” and “very easy”. Data were available 385,949 unrelated participants of European descent after quality control. Ease of getting up in the morning was analyzed as a continuous outcome, with four categories |
| Daytime napping | Daytime napping was assessed by asking: “Do you have a nap during the day?”. The three possible answers included categories “never/rarely”, “sometimes” and “usually”. Daytime napping was analyzed as a dichotomous trait (“never/rarely” and “sometimes”, vs. “usually”). |
| Daytime sleepiness/Dozing | Daytime sleepiness (dozing) was assessed by asking: “How likely are you to doze off or fall asleep during the daytime when you don't mean to? (e.g. when working, reading or driving)”. The possible answering options were: “never/rarely”, “sometimes”, “often” and “all of the time”. The answers were analyzed as a dichotomous trait (“never/rarely” and “sometimes” vs. “often” and “all the time”). |
| Snoring | Snoring was assessed by asking: "Does your partner or a close relative or friend complain about your snoring?". Participants could answer with “yes” or “no” |

## Supplementary Table3 The 20 functional categories used in partitional genetic correlation

| Annotation | Category | Online source |
| --- | --- | --- |
| DNase I | Open Chromatin | http://hgdownload.cse.ucsc.edu/goldenPath/hg19/encodeDCC/wgEncodeRegDnaseClustered/ |
| FAIRE |  | http://ftp.ebi.ac.uk/pub/databases/ensembl/encode/integration_data_jan2011/byDataType/openchrom/jan2011/faire_fseq_peaks/ |
| H3k4mel | Histone Modification | http://genome.ucsc.edu/cgi-bin/hgFileUi?db=hg19&g=wgEncodeBroadHistone/ |
| H3k4me2 |  |  |
| H3k4me3 |  |  |
| H3k9ac |  |  |
| H3k27ac |  |  |
| H3k27me3 |  |  |
| H3k36me3 |  |  |
| H4k20mel |  |  |
| CEBPB | TFBS | http://hgdownload.cse.ucsc.edu/goldenPath/hg19/encodeDCC/wgEncodeRegTfbsClustered/ |
| CTCF |  |  |
| EP300 |  |  |
| FOS |  |  |
| FOXA1 |  |  |
| JUND |  |  |
| MAX |  |  |
| MYC |  |  |
| POLR2A |  |  |
| RAD21 |  |  |

## Supplementary Table4 The 22 autosomes annotations used in partitional genetic correlation

| Predicted functional proportion for each autosome using  0.5 as the cutoff | | | |
| --- | --- | --- | --- |
| **Chromosome** | **Proportion** | **Chromosome** | **Proportion** |
| 1 | 0.332 | 12 | 0.307 |
| 2 | 0.331 | 13 | 0.365 |
| 3 | 0.356 | 14 | 0.344 |
| 4 | 0.293 | 15 | 0.392 |
| 5 | 0.374 | 16 | 0.338 |
| 6 | 0.316 | 17 | 0.334 |
| 7 | 0.321 | 18 | 0.362 |
| 8 | 0.328 | 19 | 0.313 |
| 9 | 0.388 | 20 | 0.340 |
| 10 | 0.337 | 21 | 0.331 |
| 11 | 0.322 | 22 | 0.383 |
| proportion overall average: **0.333** | | | |

Note: For each position in the human genome there is a continuous annotation score between 0 and 1. In our case, we chose 0.5 as a threshold to discretize genome-wide continuous annotation into binary annotation.

## Supplementary Table5 The ten broadly-defined tissue types annotations and 66 epigenetic annotations used in partitional genetic correlation

| Epigenome ID | Order | Tissue Type | Cell Type |
| --- | --- | --- | --- |
| E029&E124* | 1 | Blood | Monocytes CD14+ |
| E032 | 2 | Blood | B cells CD19+ |
| E034 | 3 | Blood | T cells CD3+ |
| E037 | 4 | Blood | T helper memory cells from peripheral blood CD4+ |
| E038 | 5 | Blood | T helper naive cells CD4+ |
| E039 | 6 | Blood | T helper naive cells CD4+ CD25- CD45RA+ |
| E040 | 7 | Blood | T helper memory cells CD4+ CD25- CD45RO+ |
| E041 | 8 | Blood | T helper cells PMA-I stimulated CD4+ CD25- IL17- |
| E042 | 9 | Blood | T helper 17 cells PMA-I stimulated CD4+ CD25- IL17+ |
| E043 | 10 | Blood | T helper cells CD4+ CD25- |
| E044 | 11 | Blood | T regulatory cells CD4+ CD25+ CD127- |
| E045 | 12 | Blood | T cells effector/memory CD4+ CD25int CD127+ |
| E046 | 13 | Blood | Natural killer cells CD56+ |
| E047 | 14 | Blood | T naive cells CD8+ |
| E048 | 15 | Blood | T memory cells CD8+ |
| E051 | 16 | Blood | Hematopoietic stem cells G-CSF-mobilized CD34+ |
| E062 | 17 | Blood | Mononuclear cells from peripheral blood |
| E116 | 18 | Blood | GM12878 lymphoblastoid cells |
| E026 | 19 | Bone/connective | Bone marrow derived cultured mesenchymal stem cells |
| E049 | 20 | Bone/connective | Mesenchymal stem cell derived chondrocyte cultured cells |
| E129 | 21 | Bone/connective | Osteoblast primary cells |
| E067 | 22 | Brain | Brain angular gyrus |
| E068 | 23 | Brain | Brain anterior caudate |
| E069 | 24 | Brain | Brain cingulate gyrus |
| E071 | 25 | Brain | Brain hippocampus middle |
| E072 | 26 | Brain | Brain inferior temporal lobe |
| E073 | 27 | Brain | Brain dorsolateral prefrontal cortex |
| E074 | 28 | Brain | Brain substantia nigra |
| E125 | 29 | Brain | NH-A astrocytes |
| E027 | 30 | Epithelium | Breast myoepithelial primary cells |
| E028 | 31 | Epithelium | Breast variant human mammary epithelial cells (vHMEC) |
| E119 | 32 | Epithelium | HMEC mammary epithelial primary cells |
| E023 | 33 | Fat | Mesenchymal stem cell derived adipocyte cultured cells |
| E025 | 34 | Fat | Adipose derived mesenchymal stem cell cultured cells |
| E063 | 35 | Fat | Adipose nuclei |
| E075 | 36 | GI | Colonic mucosa |
| E076 | 37 | GI | Colon smooth muscle |
| E077 | 38 | GI | Duodenum mucosa |
| E078 | 39 | GI | Duodenum smooth muscle |
| E079 | 40 | GI | Esophagus |
| E094 | 41 | GI | Gastric |
| E101&E102* | 42 | GI | Rectal mucosa |
| E103 | 43 | GI | Rectal smooth muscle |
| E106 | 44 | GI | Sigmoid colon |
| E109 | 45 | GI | Small intestine |
| E110 | 46 | GI | Stomach mucosa |
| E111 | 47 | GI | Stomach smooth muscle |
| E095 | 48 | Heart | Left ventricle |
| E104 | 49 | Heart | Right atrium |
| E105 | 50 | Heart | Right ventricle |
| E066 | 51 | Liver | Liver |
| E096 | 52 | Lung | Lung |
| E128 | 53 | Lung | NHLF lung fibroblast primary cells |
| E052 | 54 | Muscle | Muscle satellite cultured cells |
| E100 | 55 | Muscle | Psoas muscle |
| E107&E108* | 56 | Muscle | Skeletal muscle |
| E120 | 57 | Muscle | HSMM skeletal muscle myoblasts cells |
| E121 | 58 | Muscle | HSMM cell derived skeletal muscle myotubes cells |
| E097 | 59 | Ovary | Ovary |
| E087 | 60 | Pancreas | Pancreatic islets |
| E098 | 61 | Pancreas | Pancreas |
| E126 | 62 | Skin | NHDF-Ad adult dermal fibroblast primary cells |
| E127 | 63 | Skin | NHEK-epidermal keratinocyteprimary cells |
| E113 | 64 | Spleen | Spleen |
| E112 | 65 | Thymus | Thymus |
| E065 | 66 | Vascular | Aorta |
|  |  | ESC | Embryonic stem cells |
|  |  | fetal | fetal cells |

Note: * Multiple epigenomes for the same cell type were combined. In our case, we included nine broadly defined tissue categories (i.e., blood, brain,

epithelium, fetal, embryonic stem cells(ESC), gastrointestinal(GI), heart, lung, and muscle). The 66 included epigenetic cell types have been numbered in the table (‘Order’ column)

## Supplementary Table6 Fine-mapping 99% credible-set of sentinel SNP from cross-trait meta-analysis between Alzheimer's disease (AD) and insomnia.

| Sentinel.SNP | Credible-set SNPs | CHR | BP | Alzheimer's disease | | | insomnia | | |
| --- | --- | --- | --- | --- | --- | --- | --- | --- | --- |
|  |  |  |  | **GWAS-p-value** | **probNorm** | **cumSum** | **GWAS-p-value** | **probNorm** | **cumSum** |
| rs11234556 | rs11234556 | 11 | 8.6E+07 | 3.92429E-17 | 0.007932 | 0.948715 | 0.003751 | 0.022383 | 0.429244 |
|  | rs1973630 | 11 | 8.6E+07 | 2.77518E-17 | 0.011164 | 0.911268 | 0.07281 | 0.001677 | 0.94691 |
|  | rs2508691 | 11 | 8.6E+07 | 3.96675E-17 | 0.007848 | 0.956564 | 0.07314 | 0.001671 | 0.948581 |
|  | rs2508696 | 11 | 8.6E+07 | 5.43466E-18 | 0.0558 | 0.61419 | 0.08064 | 0.001543 | 0.961334 |
|  | rs3844143 | 11 | 8.6E+07 | 9.02186E-17 | 0.00349 | 0.976682 | 0.004019 | 0.021022 | 0.450266 |
|  | rs471470 | 11 | 8.6E+07 | 2.24909E-16 | 0.001418 | 0.990353 | 0.03667 | 0.002977 | 0.917484 |
|  | rs472486 | 11 | 8.6E+07 | 8.95701E-17 | 0.003515 | 0.973193 | 0.1003 | 0.001295 | 0.974476 |
|  | rs493254 | 11 | 8.6E+07 | 3.09643E-18 | 0.097234 | 0.558391 | 0.07841 | 0.001579 | 0.956659 |
|  | rs536841 | 11 | 8.6E+07 | 2.37202E-17 | 0.013034 | 0.88886 | 0.1106 | 0.001198 | 0.986883 |
|  | rs561646 | 11 | 8.6E+07 | 2.83034E-17 | 0.01095 | 0.922218 | 0.07844 | 0.001578 | 0.958237 |
|  | rs561655 | 11 | 8.6E+07 | 2.05222E-17 | 0.015036 | 0.847717 | 0.106 | 0.001239 | 0.980827 |
|  | rs587038 | 11 | 8.6E+07 | 1.26117E-16 | 0.002508 | 0.985302 | 0.111 | 0.001195 | 0.988077 |
|  | rs602222 | 11 | 8.6E+07 | 7.58628E-18 | 0.040147 | 0.756729 | 0.1115 | 0.001191 | 0.989268 |
|  | rs606505 | 11 | 8.6E+07 | 6.72658E-17 | 0.004662 | 0.966093 | 0.1014 | 0.001284 | 0.977046 |
|  | rs609903 | 11 | 8.6E+07 | 1.30986E-17 | 0.023418 | 0.815364 | 0.07997 | 0.001554 | 0.959791 |
|  | rs636355 | 11 | 8.6E+07 | 8.66332E-18 | 0.035216 | 0.791945 | 0.07519 | 0.001634 | 0.951871 |
|  | rs639012 | 11 | 8.6E+07 | 3.04049E-17 | 0.010203 | 0.93242 | 0.08899 | 0.001425 | 0.973181 |
|  | rs645299 | 11 | 8.6E+07 | 6.43831E-17 | 0.004868 | 0.961431 | 0.1145 | 0.001166 | 0.990434 |
|  | rs669813 | 11 | 8.6E+07 | 6.20407E-18 | 0.048963 | 0.716583 | 0.08419 | 0.00149 | 0.965882 |
|  | rs673751 | 11 | 8.6E+07 | 1.15456E-16 | 0.002736 | 0.982794 | 0.1104 | 0.0012 | 0.985684 |
|  | rs676733 | 11 | 8.6E+07 | 1.77849E-17 | 0.017318 | 0.832681 | 0.07659 | 0.001609 | 0.953481 |
|  | rs677909 | 11 | 8.6E+07 | 2.15103E-17 | 0.014355 | 0.862072 | 0.1017 | 0.001281 | 0.978327 |
|  | rs682928 | 11 | 8.6E+07 | 5.67896E-18 | 0.053429 | 0.66762 | 0.06933 | 0.001746 | 0.943544 |
|  | rs694011 | 11 | 8.6E+07 | 2.24622E-17 | 0.013754 | 0.875826 | 0.08531 | 0.001475 | 0.97032 |
|  | rs867611 | 11 | 8.6E+07 | 1.48189E-18 | 0.201296 | 0.201296 | 0.07395 | 0.001656 | 0.950237 |
| rs150567157 | rs145903417 | 19 | 4.6E+07 | 3.19536E-23 | 0.498761 | 0.498761 | 0.04682 | 0.079547 | 1 |
|  | rs150685845 | 19 | 4.6E+07 | 3.19683E-23 | 0.498535 | 0.997296 | 0.0197 | 0.167262 | 0.701867 |
| rs186110295 | rs186110295 | 19 | 4.5E+07 | 4.44511E-11 | 0.994836 | 0.994836 | 0.005563 | 0.90422 | 0.90422 |
| rs2249152 | rs10401977 | 19 | 5038785 | 0.000267392 | 0.021543 | 0.660337 | 2.12E-06 | 0.008256 | 0.737842 |
|  | rs10411421 | 19 | 5036564 | 0.000297407 | 0.019499 | 0.721391 | 1.51E-06 | 0.011469 | 0.678466 |
|  | rs10853982 | 19 | 5088080 | 0.000187988 | 0.029988 | 0.573346 | 3.02E-06 | 0.005883 | 0.875873 |
|  | rs11085110 | 19 | 5037415 | 0.000283669 | 0.020383 | 0.701892 | 7.13E-07 | 0.023575 | 0.402798 |
|  | rs11085112 | 19 | 5085862 | 0.000123807 | 0.044432 | 0.302197 | 1.66E-06 | 0.010452 | 0.710091 |
|  | rs11666210 | 19 | 5081231 | 7.95656E-05 | 0.067447 | 0.145437 | 5.48E-06 | 0.003326 | 0.946335 |
|  | rs11670830 | 19 | 5085485 | 0.00014352 | 0.038657 | 0.467102 | 1.44E-06 | 0.011997 | 0.666997 |
|  | rs11880565 | 19 | 5087167 | 0.000147082 | 0.037775 | 0.543358 | 7.5E-06 | 0.002464 | 0.975509 |
|  | rs12459464 | 19 | 5013831 | 0.004672171 | 0.001534 | 0.944835 | 8.15E-06 | 0.002276 | 0.984981 |
|  | rs1471508 | 19 | 5074746 | 0.000369202 | 0.015927 | 0.79123 | 5.85E-07 | 0.028554 | 0.19613 |
|  | rs16992773 | 19 | 5052147 | 0.000823945 | 0.007541 | 0.913309 | 2.29E-06 | 0.007684 | 0.753528 |
|  | rs1706980 | 19 | 4973584 | 0.004971207 | 0.001451 | 0.949343 | 8.42E-07 | 0.020089 | 0.486406 |
|  | rs174655 | 19 | 4980597 | 0.003867789 | 0.001821 | 0.928326 | 2.95E-06 | 0.006013 | 0.864034 |
|  | rs197139 | 19 | 4996460 | 0.006632817 | 0.001118 | 0.978131 | 4.39E-06 | 0.00411 | 0.921115 |
|  | rs197143 | 19 | 4989028 | 0.006990817 | 0.001067 | 0.982486 | 4.75E-06 | 0.00381 | 0.928822 |
|  | rs197151 | 19 | 4985911 | 0.005519818 | 0.00132 | 0.957626 | 2.57E-06 | 0.006875 | 0.774771 |
|  | rs197153 | 19 | 4984351 | 0.006784483 | 0.001096 | 0.980342 | 6.91E-07 | 0.024315 | 0.379223 |
|  | rs2240679 | 19 | 5041316 | 0.000357808 | 0.016401 | 0.775303 | 6.67E-07 | 0.025143 | 0.32982 |
|  | rs2249152 | 19 | 5061052 | 9.43319E-05 | 0.057426 | 0.202864 | 2.55E-06 | 0.006919 | 0.767896 |
|  | rs2531131 | 19 | 5010022 | 0.005582865 | 0.001306 | 0.960244 | 6.4E-06 | 0.002867 | 0.964991 |
|  | rs2613769 | 19 | 5075034 | 0.000144219 | 0.038481 | 0.505583 | 3.11E-06 | 0.005723 | 0.887353 |
|  | rs2613770 | 19 | 5075227 | 0.000263898 | 0.021811 | 0.617208 | 6.48E-07 | 0.025853 | 0.304677 |
|  | rs2613776 | 19 | 5076777 | 9.89323E-05 | 0.054902 | 0.257765 | 3.61E-06 | 0.004961 | 0.908435 |
|  | rs263047 | 19 | 5044972 | 0.000470189 | 0.012708 | 0.834783 | 3.18E-06 | 0.005609 | 0.892962 |
|  | rs263048 | 19 | 5017703 | 0.004973317 | 0.00145 | 0.950794 | 5.43E-06 | 0.003355 | 0.943009 |
|  | rs263049 | 19 | 5017567 | 0.006073392 | 0.001211 | 0.967714 | 6.06E-06 | 0.00302 | 0.962125 |
|  | rs263050 | 19 | 5016619 | 0.005307671 | 0.001367 | 0.95498 | 5.55E-06 | 0.003284 | 0.949619 |
|  | rs263064 | 19 | 5026004 | 0.003446557 | 0.002023 | 0.920812 | 5.79E-06 | 0.003154 | 0.955957 |
|  | rs263066 | 19 | 5027230 | 0.005180126 | 0.001398 | 0.953613 | 6.51E-06 | 0.002821 | 0.967813 |
|  | rs263068 | 19 | 5029012 | 0.004673856 | 0.001534 | 0.946369 | 7.38E-06 | 0.002503 | 0.973045 |
|  | rs35218861 | 19 | 5074003 | 6.82393E-05 | 0.07799 | 0.07799 | 7.94E-06 | 0.002333 | 0.982705 |
|  | rs36115836 | 19 | 5063649 | 0.000135983 | 0.040673 | 0.428445 | 4.24E-06 | 0.004251 | 0.917005 |
|  | rs389354 | 19 | 4972982 | 0.006487517 | 0.001141 | 0.974759 | 8.36E-07 | 0.020242 | 0.466317 |
|  | rs390027 | 19 | 4972771 | 0.006561161 | 0.001129 | 0.975888 | 8.56E-07 | 0.019784 | 0.50619 |
|  | rs400263 | 19 | 4971999 | 0.006190604 | 0.00119 | 0.970113 | 1.2E-06 | 0.014264 | 0.616815 |
|  | rs401002 | 19 | 4972849 | 0.006597696 | 0.001124 | 0.977012 | 7.6E-07 | 0.022184 | 0.424981 |
|  | rs418115 | 19 | 4977478 | 0.004306202 | 0.001652 | 0.938523 | 2.36E-06 | 0.007449 | 0.760977 |
|  | rs4807005 | 19 | 4985526 | 0.006918421 | 0.001077 | 0.981419 | 3.26E-06 | 0.005473 | 0.898435 |
|  | rs4807673 | 19 | 4976121 | 0.005082235 | 0.001422 | 0.952215 | 3.09E-06 | 0.005757 | 0.88163 |
|  | rs4807674 | 19 | 4976357 | 0.004149654 | 0.001709 | 0.933512 | 2.88E-06 | 0.006159 | 0.845781 |
|  | rs4807679 | 19 | 5016399 | 0.005606719 | 0.001301 | 0.961545 | 7.54E-06 | 0.002451 | 0.97796 |
|  | rs4807684 | 19 | 5065780 | 0.000129002 | 0.042743 | 0.387772 | 3.55E-06 | 0.00504 | 0.903475 |
|  | rs495555 | 19 | 4974846 | 0.006445913 | 0.001148 | 0.973618 | 5.45E-07 | 0.030578 | 0.167577 |
|  | rs523064 | 19 | 4977060 | 0.004708818 | 0.001524 | 0.947893 | 5.73E-06 | 0.003184 | 0.952803 |
|  | rs56123572 | 19 | 4979988 | 0.004036182 | 0.001752 | 0.930078 | 2.9E-06 | 0.006122 | 0.851903 |
|  | rs57063808 | 19 | 5071529 | 0.000260827 | 0.022051 | 0.595398 | 5.17E-06 | 0.003514 | 0.939654 |
|  | rs57308607 | 19 | 5046168 | 0.000720193 | 0.008544 | 0.905769 | 1.01E-06 | 0.016865 | 0.55752 |
|  | rs59541213 | 19 | 5004191 | 0.006266331 | 0.001177 | 0.97247 | 4.83E-06 | 0.003756 | 0.932577 |
|  | rs62112545 | 19 | 4971945 | 0.005492241 | 0.001326 | 0.956306 | 1.63E-06 | 0.010661 | 0.689127 |
|  | rs62112547 | 19 | 4972478 | 0.007133395 | 0.001048 | 0.984586 | 1.26E-06 | 0.013663 | 0.630478 |
|  | rs62112548 | 19 | 4974271 | 0.006652114 | 0.001116 | 0.979246 | 5.87E-07 | 0.028432 | 0.224562 |
|  | rs62114274 | 19 | 4980132 | 0.003711935 | 0.001891 | 0.924652 | 2.98E-06 | 0.005957 | 0.86999 |
|  | rs62114275 | 19 | 4980171 | 0.003371716 | 0.002063 | 0.918789 | 2.6E-06 | 0.006794 | 0.788384 |
|  | rs62114282 | 19 | 5003033 | 0.008599127 | 0.000886 | 0.989472 | 5.1E-06 | 0.003562 | 0.93614 |
|  | rs62114283 | 19 | 5003275 | 0.0059222 | 0.001239 | 0.964055 | 7.66E-06 | 0.002413 | 0.980372 |
|  | rs62114286 | 19 | 5005387 | 0.006082066 | 0.001209 | 0.968923 | 8.77E-06 | 0.002121 | 0.991504 |
|  | rs62114350 | 19 | 5016881 | 0.005556089 | 0.001312 | 0.958938 | 8.2E-06 | 0.002263 | 0.987244 |
|  | rs62114353 | 19 | 5027200 | 0.00458115 | 0.001562 | 0.943301 | 8.69E-06 | 0.002139 | 0.989383 |
|  | rs62114356 | 19 | 5033966 | 0.000534456 | 0.011277 | 0.857865 | 1.71E-06 | 0.010192 | 0.720283 |
|  | rs62114357 | 19 | 5034179 | 0.000672375 | 0.009108 | 0.888315 | 9.95E-07 | 0.017105 | 0.540655 |
|  | rs62114358 | 19 | 5034492 | 0.000688499 | 0.008909 | 0.897224 | 1.42E-06 | 0.012143 | 0.655 |
|  | rs62115605 | 19 | 5088927 | 0.001942465 | 0.003417 | 0.916726 | 1.18E-06 | 0.014473 | 0.60255 |
|  | rs632616 | 19 | 4976335 | 0.004106062 | 0.001725 | 0.931803 | 2.86E-06 | 0.0062 | 0.839622 |
|  | rs635271 | 19 | 4976985 | 0.004498663 | 0.001588 | 0.941739 | 2.7E-06 | 0.006554 | 0.808272 |
|  | rs6417190 | 19 | 5009028 | 0.006051012 | 0.001215 | 0.966503 | 6.74E-06 | 0.002729 | 0.970542 |
|  | rs7253495 | 19 | 5065041 | 0.000382341 | 0.015415 | 0.822075 | 2.19E-06 | 0.008003 | 0.745845 |
|  | rs7255647 | 19 | 5000822 | 0.007099405 | 0.001052 | 0.983538 | 4.17E-06 | 0.004319 | 0.912755 |
|  | rs72620527 | 19 | 5039309 | 0.000272388 | 0.021173 | 0.681509 | 6.47E-07 | 0.025914 | 0.278824 |
|  | rs72620528 | 19 | 5040480 | 0.000381937 | 0.01543 | 0.80666 | 8.01E-07 | 0.021094 | 0.446075 |
|  | rs730291 | 19 | 5070809 | 0.000308082 | 0.018866 | 0.740257 | 5.8E-06 | 0.003148 | 0.959105 |
|  | rs734925 | 19 | 5054027 | 0.000311994 | 0.018644 | 0.758902 | 6.69E-07 | 0.025088 | 0.354908 |
|  | rs753842 | 19 | 4977744 | 0.004375338 | 0.001628 | 0.940151 | 2.83E-06 | 0.006274 | 0.82097 |
|  | rs78183186 | 19 | 5065896 | 0.000266843 | 0.021585 | 0.638793 | 1.65E-06 | 0.010512 | 0.699639 |
|  | rs8099963 | 19 | 4975564 | 0.004204178 | 0.001688 | 0.9352 | 2.66E-06 | 0.006656 | 0.801718 |
|  | rs8100907 | 19 | 4975636 | 0.003794832 | 0.001853 | 0.926505 | 2.65E-06 | 0.006678 | 0.795061 |
|  | rs8105691 | 19 | 4985768 | 0.005948915 | 0.001234 | 0.965288 | 2.76E-06 | 0.006424 | 0.814696 |
|  | rs858353 | 19 | 4978466 | 0.003587839 | 0.00195 | 0.922762 | 2.59E-06 | 0.006819 | 0.78159 |
|  | rs9636178 | 19 | 5033752 | 0.000508882 | 0.011804 | 0.846587 | 1.88E-06 | 0.009303 | 0.729586 |
|  | rs9676980 | 19 | 4974058 | 0.006249887 | 0.00118 | 0.971293 | 5.89E-07 | 0.028348 | 0.25291 |
| rs606757 | rs2627641 | 19 | 4.6E+07 | 8.18813E-23 | 0.114092 | 0.582644 | 0.06162 | 0.02879 | 0.873305 |
|  | rs4803806 | 19 | 4.6E+07 | 1.20849E-22 | 0.077607 | 0.837981 | 0.05431 | 0.031976 | 0.722927 |
|  | rs582747 | 19 | 4.6E+07 | 2.22594E-22 | 0.042396 | 0.999535 | 0.05798 | 0.030281 | 0.784107 |
|  | rs595290 | 19 | 4.6E+07 | 4.64964E-23 | 0.199786 | 0.199786 | 0.05832 | 0.030135 | 0.844515 |
|  | rs597668 | 19 | 4.6E+07 | 1.11209E-22 | 0.084263 | 0.760374 | 0.058 | 0.030273 | 0.81438 |
|  | rs598183 | 19 | 4.6E+07 | 1.2834E-22 | 0.073122 | 0.911102 | 0.0532 | 0.032532 | 0.690951 |
|  | rs605003 | 19 | 4.6E+07 | 6.05558E-23 | 0.153805 | 0.353592 | 0.05659 | 0.030899 | 0.753826 |
|  | rs7248787 | 19 | 4.6E+07 | 1.00152E-22 | 0.093467 | 0.676111 | 0.07724 | 0.023913 | 0.97709 |
|  | rs7249082 | 19 | 4.6E+07 | 8.12563E-23 | 0.11496 | 0.468552 | 0.06788 | 0.026581 | 0.927146 |
|  | rs7249570 | 19 | 4.6E+07 | 2.04819E-22 | 0.046036 | 0.957139 | 0.06583 | 0.02726 | 0.900565 |
| rs9268428 | rs1980495 | 6 | 3.2E+07 | 4.07363E-09 | 0.007944 | 0.857299 | 0.001545 | 0.007353 | 0.265211 |
|  | rs2076520 | 6 | 3.2E+07 | 1.5705E-08 | 0.002139 | 0.970307 | 0.001517 | 0.007478 | 0.198514 |
|  | rs2076522 | 6 | 3.2E+07 | 9.46638E-09 | 0.003498 | 0.937695 | 0.001517 | 0.007478 | 0.191036 |
|  | rs2076524 | 6 | 3.2E+07 | 1.57022E-08 | 0.002139 | 0.968168 | 0.00152 | 0.007464 | 0.220919 |
|  | rs2076525 | 6 | 3.2E+07 | 1.85305E-08 | 0.001822 | 0.97804 | 0.001647 | 0.006932 | 0.532559 |
|  | rs2294880 | 6 | 3.2E+07 | 1.37714E-08 | 0.00243 | 0.956854 | 0.001378 | 0.00817 | 0.074483 |
|  | rs2395155 | 6 | 3.2E+07 | 1.83152E-09 | 0.017303 | 0.371632 | 0.001562 | 0.007279 | 0.360374 |
|  | rs2395156 | 6 | 3.2E+07 | 2.29757E-09 | 0.013874 | 0.601397 | 0.001546 | 0.007348 | 0.294618 |
|  | rs2395157 | 6 | 3.2E+07 | 3.49251E-09 | 0.009228 | 0.83298 | 0.001581 | 0.007198 | 0.468958 |
|  | rs2894252 | 6 | 3.2E+07 | 4.29522E-09 | 0.007545 | 0.880059 | 0.001536 | 0.007392 | 0.235717 |
|  | rs3763307 | 6 | 3.2E+07 | 2.4914E-08 | 0.001367 | 0.984157 | 0.001706 | 0.006712 | 0.546104 |
|  | rs3763311 | 6 | 3.2E+07 | 7.86979E-10 | 0.039424 | 0.080763 | 0.00231 | 0.005082 | 0.588015 |
|  | rs3763316 | 6 | 3.2E+07 | 5.60715E-09 | 0.005821 | 0.912915 | 0.001096 | 0.010094 | 0.066313 |
|  | rs3793126 | 6 | 3.2E+07 | 1.35576E-08 | 0.002467 | 0.954423 | 0.001519 | 0.007469 | 0.213455 |
|  | rs3806157 | 6 | 3.2E+07 | 2.00601E-08 | 0.001687 | 0.979726 | 0.003242 | 0.003728 | 0.709311 |
|  | rs3817962 | 6 | 3.2E+07 | 1.6489E-08 | 0.00204 | 0.972348 | 0.001506 | 0.007528 | 0.168558 |
|  | rs3817963 | 6 | 3.2E+07 | 2.43357E-08 | 0.001398 | 0.98279 | 0.001604 | 0.007103 | 0.483255 |
|  | rs8180659 | 6 | 3.2E+07 | 1.91453E-09 | 0.016572 | 0.388204 | 0.001545 | 0.007353 | 0.272564 |
|  | rs8180664 | 6 | 3.2E+07 | 2.11017E-09 | 0.015073 | 0.529701 | 0.001558 | 0.007296 | 0.345816 |
|  | rs9268400 | 6 | 3.2E+07 | 2.94954E-09 | 0.010878 | 0.813492 | 0.001565 | 0.007266 | 0.374915 |
|  | rs9268401 | 6 | 3.2E+07 | 4.29917E-09 | 0.007538 | 0.887598 | 0.00233 | 0.005042 | 0.593057 |
|  | rs9268403 | 6 | 3.2E+07 | 2.03162E-09 | 0.015641 | 0.468599 | 0.001508 | 0.007519 | 0.176076 |
|  | rs9268404 | 6 | 3.2E+07 | 1.29666E-09 | 0.024227 | 0.2722 | 0.001608 | 0.007087 | 0.504525 |
|  | rs9268405 | 6 | 3.2E+07 | 2.06916E-09 | 0.015364 | 0.49945 | 0.001578 | 0.007211 | 0.46176 |
|  | rs9268406 | 6 | 3.2E+07 | 1.9888E-09 | 0.015969 | 0.452958 | 0.001578 | 0.007211 | 0.454549 |
|  | rs9268407 | 6 | 3.2E+07 | 2.0523E-09 | 0.015487 | 0.484086 | 0.001578 | 0.007211 | 0.447338 |
|  | rs9268409 | 6 | 3.2E+07 | 3.95724E-09 | 0.008171 | 0.849355 | 0.000799 | 0.013533 | 0.056219 |
|  | rs9268412 | 6 | 3.2E+07 | 3.94145E-09 | 0.008203 | 0.841183 | 0.000795 | 0.013596 | 0.042686 |
|  | rs9268413 | 6 | 3.2E+07 | 3.23487E-08 | 0.001061 | 0.986519 | 0.000769 | 0.01401 | 0.029091 |
|  | rs9268414 | 6 | 3.2E+07 | 3.71496E-08 | 0.000927 | 0.988375 | 0.000711 | 0.01508 | 0.01508 |
|  | rs9268415 | 6 | 3.2E+07 | 1.94374E-09 | 0.016329 | 0.404533 | 0.001558 | 0.007296 | 0.33852 |
|  | rs9268416 | 6 | 3.2E+07 | 2.19925E-09 | 0.014478 | 0.559174 | 0.001566 | 0.007262 | 0.389443 |
|  | rs9268417 | 6 | 3.2E+07 | 2.7396E-09 | 0.011689 | 0.7572 | 0.001533 | 0.007406 | 0.228325 |
|  | rs9268418 | 6 | 3.2E+07 | 2.38224E-09 | 0.013394 | 0.656079 | 0.001516 | 0.007482 | 0.183558 |
|  | rs9268420 | 6 | 3.2E+07 | 2.7812E-09 | 0.011519 | 0.768719 | 0.001537 | 0.007388 | 0.250497 |
|  | rs9268421 | 6 | 3.2E+07 | 4.73389E-09 | 0.006864 | 0.894461 | 0.001423 | 0.007932 | 0.106419 |
|  | rs9268422 | 6 | 3.2E+07 | 4.26087E-09 | 0.007604 | 0.872515 | 0.001563 | 0.007275 | 0.367649 |
|  | rs9268423 | 6 | 3.2E+07 | 4.25655E-09 | 0.007612 | 0.86491 | 0.001423 | 0.007932 | 0.098487 |
|  | rs9268424 | 6 | 3.2E+07 | 2.8123E-09 | 0.011395 | 0.780114 | 0.001554 | 0.007313 | 0.331224 |
|  | rs9268425 | 6 | 3.2E+07 | 2.23005E-09 | 0.014283 | 0.573457 | 0.001554 | 0.007313 | 0.316597 |
|  | rs9268426 | 6 | 3.2E+07 | 3.13225E-09 | 0.01026 | 0.823752 | 0.001554 | 0.007313 | 0.323911 |
|  | rs9268427 | 6 | 3.2E+07 | 1.94818E-09 | 0.016293 | 0.420826 | 0.001543 | 0.007361 | 0.257859 |
|  | rs9268428 | 6 | 3.2E+07 | 1.24058E-09 | 0.025294 | 0.247973 | 0.001412 | 0.007989 | 0.090555 |
|  | rs9268431 | 6 | 3.2E+07 | 2.09521E-09 | 0.015178 | 0.514628 | 0.001536 | 0.007392 | 0.243109 |
|  | rs9268432 | 6 | 3.2E+07 | 2.5257E-09 | 0.012652 | 0.721175 | 0.001565 | 0.007266 | 0.382181 |
|  | rs9268436 | 6 | 3.2E+07 | 2.31215E-09 | 0.013789 | 0.629049 | 0.001569 | 0.007249 | 0.411198 |
|  | rs9268439 | 6 | 3.2E+07 | 1.96417E-09 | 0.016164 | 0.436989 | 0.00155 | 0.007331 | 0.309284 |
|  | rs9268446 | 6 | 3.2E+07 | 2.71177E-09 | 0.011806 | 0.745511 | 0.001562 | 0.007279 | 0.353095 |
|  | rs9268447 | 6 | 3.2E+07 | 2.83962E-09 | 0.011288 | 0.791402 | 0.001549 | 0.007335 | 0.301953 |
|  | rs9268448 | 6 | 3.2E+07 | 1.70099E-09 | 0.018596 | 0.354328 | 0.001447 | 0.00781 | 0.145726 |
|  | rs9268449 | 6 | 3.2E+07 | 1.67353E-09 | 0.018893 | 0.335733 | 0.001609 | 0.007083 | 0.511608 |
|  | rs9268450 | 6 | 3.2E+07 | 2.12148E-09 | 0.014995 | 0.544696 | 0.001518 | 0.007473 | 0.205987 |
|  | rs9268452 | 6 | 3.2E+07 | 2.39068E-09 | 0.013348 | 0.68282 | 0.001572 | 0.007236 | 0.418435 |
|  | rs9268453 | 6 | 3.2E+07 | 2.3388E-09 | 0.013636 | 0.642685 | 0.001572 | 0.007236 | 0.432907 |
|  | rs9268454 | 6 | 3.2E+07 | 2.29944E-09 | 0.013863 | 0.61526 | 0.001572 | 0.007236 | 0.425671 |
|  | rs9268456 | 6 | 3.2E+07 | 2.38241E-09 | 0.013393 | 0.669472 | 0.001576 | 0.007219 | 0.440127 |
|  | rs9268457 | 6 | 3.2E+07 | 2.85932E-09 | 0.011212 | 0.802614 | 0.001506 | 0.007528 | 0.16103 |
|  | rs9268458 | 6 | 3.2E+07 | 1.81252E-08 | 0.001861 | 0.976218 | 0.001582 | 0.007194 | 0.476152 |
|  | rs9268459 | 6 | 3.2E+07 | 2.55093E-09 | 0.01253 | 0.733705 | 0.001434 | 0.007876 | 0.122205 |
|  | rs9268461 | 6 | 3.2E+07 | 2.45201E-09 | 0.013022 | 0.695842 | 0.001607 | 0.007091 | 0.490346 |
|  | rs9268462 | 6 | 3.2E+07 | 2.51978E-09 | 0.012681 | 0.708523 | 0.001607 | 0.007091 | 0.497438 |
|  | rs9268474 | 6 | 3.2E+07 | 1.50057E-09 | 0.021012 | 0.316839 | 0.001394 | 0.008084 | 0.082567 |
|  | rs9268475 | 6 | 3.2E+07 | 2.2655E-09 | 0.014066 | 0.587523 | 0.001644 | 0.006944 | 0.525626 |
|  | rs9268477 | 6 | 3.2E+07 | 1.39948E-08 | 0.002393 | 0.961671 | 0.001755 | 0.006539 | 0.559333 |
|  | rs9268478 | 6 | 3.2E+07 | 1.52808E-08 | 0.002197 | 0.963867 | 0.001673 | 0.006833 | 0.539392 |
|  | rs9268480 | 6 | 3.2E+07 | 1.24656E-08 | 0.002677 | 0.946949 | 0.001611 | 0.007075 | 0.518682 |
|  | rs9268481 | 6 | 3.2E+07 | 1.38052E-08 | 0.002424 | 0.959278 | 0.001427 | 0.007911 | 0.11433 |
|  | rs9268482 | 6 | 3.2E+07 | 1.55348E-08 | 0.002162 | 0.966029 | 0.001436 | 0.007865 | 0.130071 |
|  | rs9268491 | 6 | 3.2E+07 | 1.6748E-08 | 0.00201 | 0.974357 | 0.001569 | 0.007249 | 0.403949 |
|  | rs9268492 | 6 | 3.2E+07 | 7.43642E-09 | 0.004423 | 0.922662 | 0.002911 | 0.004113 | 0.658892 |
|  | rs9268493 | 6 | 3.2E+07 | 1.33181E-08 | 0.002511 | 0.949459 | 0.001888 | 0.006114 | 0.571705 |
|  | rs9268499 | 6 | 3.2E+07 | 8.36965E-09 | 0.003943 | 0.926605 | 0.002427 | 0.004857 | 0.602793 |
|  | rs9268514 | 6 | 3.2E+07 | 1.33925E-08 | 0.002497 | 0.951956 | 0.001454 | 0.007776 | 0.153502 |
|  | rs9268516 | 6 | 3.2E+07 | 8.43339E-09 | 0.003914 | 0.930519 | 0.001712 | 0.00669 | 0.552794 |
|  | rs9268838 | 6 | 3.2E+07 | 4.02338E-08 | 0.000858 | 0.990138 | 0.003694 | 0.003311 | 0.792733 |

## Supplementary Table7 Fine-mapping 99% credible-set of sentinel SNP from cross-trait meta-analysis between Alzheimer's disease (AD) and sleep duration (Sleepdur).

| Sentinel.SNP | Credible-set SNPs | CHR | BP | Alzheimer's disease | | | sleep duration | | |
| --- | --- | --- | --- | --- | --- | --- | --- | --- | --- |
|  |  |  |  | **GWAS-p-value** | **probNorm** | **cumSum** | **GWAS-p-value** | **probNorm** | **cumSum** |
| rs1081105 | rs1081105 | 19 | 45412955 | 1.2E-232 | 1 | 1 | 0.004731 | 0.035951 | 0.944367 |
| rs11672748 | rs11672748 | 19 | 45490192 | 7.25E-24 | 0.262005 | 0.262005 | 0.003237 | 0.056426 | 0.234825 |
|  | rs2075619 | 19 | 45495682 | 1.64E-23 | 0.116782 | 0.734999 | 0.003054 | 0.0595 | 0.119269 |
|  | rs3786505 | 19 | 45490570 | 2.48E-23 | 0.07768 | 0.926275 | 0.003039 | 0.059768 | 0.059768 |
|  | rs56784978 | 19 | 45499388 | 1.69E-23 | 0.113596 | 0.848595 | 0.004144 | 0.045075 | 0.526356 |
|  | rs57204168 | 19 | 45496776 | 2.61E-23 | 0.073676 | 0.999951 | 0.003445 | 0.053315 | 0.28814 |
|  | rs8111069 | 19 | 45483438 | 8.81E-24 | 0.216148 | 0.478154 | 0.003939 | 0.0472 | 0.387674 |
|  | rs909134 | 19 | 45493061 | 1.37E-23 | 0.140063 | 0.618217 | 0.003075 | 0.05913 | 0.178398 |
| rs12292911 | rs10437655 | 11 | 47391948 | 9.29E-07 | 0.088351 | 0.088351 | 8.95E-05 | 0.035083 | 0.901737 |
|  | rs10742802 | 11 | 47432725 | 1.39E-06 | 0.060089 | 0.397143 | 0.000059 | 0.052009 | 0.30718 |
|  | rs10769263 | 11 | 47417183 | 2.59E-06 | 0.032999 | 0.863243 | 5.59E-05 | 0.054737 | 0.201175 |
|  | rs10769264 | 11 | 47423340 | 2.84E-06 | 0.030152 | 0.893395 | 6.65E-05 | 0.046459 | 0.6993 |
|  | rs10838702 | 11 | 47410888 | 1.46E-06 | 0.057363 | 0.454506 | 8.44E-05 | 0.037058 | 0.866654 |
|  | rs10838705 | 11 | 47424931 | 1.48E-06 | 0.056281 | 0.510788 | 8.21E-05 | 0.038047 | 0.829596 |
|  | rs10838709 | 11 | 47447346 | 2.29E-06 | 0.037101 | 0.830244 | 6.34E-05 | 0.048585 | 0.557924 |
|  | rs11039221 | 11 | 47427739 | 2.14E-06 | 0.039682 | 0.754942 | 5.91E-05 | 0.051934 | 0.359114 |
|  | rs11039225 | 11 | 47430599 | 1.36E-06 | 0.061198 | 0.21605 | 6.69E-05 | 0.04617 | 0.74547 |
|  | rs11606287 | 11 | 47407439 | 8.76E-06 | 0.010258 | 0.99083 | 0.000462 | 0.007512 | 0.980413 |
|  | rs12292911 | 11 | 47449072 | 1.25E-06 | 0.066501 | 0.154852 | 6.27E-05 | 0.049121 | 0.509339 |
|  | rs1377416 | 11 | 47416746 | 3.66E-06 | 0.023651 | 0.917045 | 5.67E-05 | 0.053996 | 0.255171 |
|  | rs1534576 | 11 | 47419663 | 1.37E-06 | 0.060602 | 0.276652 | 5.91E-05 | 0.051901 | 0.411015 |
|  | rs2278890 | 11 | 47399602 | 6.05E-06 | 0.014613 | 0.954667 | 0.000548 | 0.006403 | 0.994178 |
|  | rs2293579 | 11 | 47440758 | 1.55E-06 | 0.054097 | 0.619085 | 5.24E-05 | 0.058248 | 0.146438 |
|  | rs34937994 | 11 | 47389638 | 3.77E-06 | 0.02301 | 0.940055 | 0.000369 | 0.00925 | 0.964335 |
|  | rs35032070 | 11 | 47408592 | 7.56E-06 | 0.01182 | 0.980572 | 0.000472 | 0.007362 | 0.987775 |
|  | rs4434960 | 11 | 47454551 | 1.54E-06 | 0.054201 | 0.564989 | 6.4E-05 | 0.048126 | 0.60605 |
|  | rs7103648 | 11 | 47461783 | 1.76E-06 | 0.047714 | 0.71526 | 6.6E-05 | 0.046792 | 0.652841 |
|  | rs7104036 | 11 | 47462140 | 1.73E-06 | 0.048461 | 0.667546 | 6.26E-05 | 0.049203 | 0.460218 |
|  | rs7947450 | 11 | 47429904 | 1.38E-06 | 0.060403 | 0.337055 | 6.71E-05 | 0.046078 | 0.791548 |
|  | rs896817 | 11 | 47394305 | 2.22E-06 | 0.038201 | 0.793143 | 0.000117 | 0.027342 | 0.929079 |
| rs1633096 | rs1610602 | 6 | 29702061 | 0.005803 | 0.062758 | 0.302976 | 7.28E-08 | 0.03103 | 0.82658 |
|  | rs1610608 | 6 | 29709407 | 0.0059 | 0.06183 | 0.55212 | 7.49E-08 | 0.030191 | 0.980372 |
|  | rs1610612 | 6 | 29710831 | 0.006079 | 0.060191 | 0.795166 | 6.18E-08 | 0.036341 | 0.669489 |
|  | rs1622223 | 6 | 29715063 | 0.006548 | 0.056295 | 0.910769 | 2.22E-07 | 0.010529 | 0.990902 |
|  | rs1633096 | 6 | 29707267 | 0.00202 | 0.164005 | 0.164005 | 3.49E-09 | 0.592782 | 0.592782 |
|  | rs1633098 | 6 | 29705028 | 0.005998 | 0.060917 | 0.674773 | 7.33E-08 | 0.030829 | 0.919446 |
|  | rs1633099 | 6 | 29704633 | 0.006078 | 0.060202 | 0.734974 | 7.35E-08 | 0.030736 | 0.950181 |
|  | rs1736915 | 6 | 29704316 | 0.004681 | 0.076213 | 0.240217 | 7.28E-08 | 0.031026 | 0.857606 |
|  | rs885942 | 6 | 29709949 | 0.00591 | 0.061736 | 0.613856 | 5.55E-08 | 0.040365 | 0.633148 |
|  | rs885943 | 6 | 29709812 | 0.006179 | 0.059309 | 0.854474 | 7.08E-08 | 0.031871 | 0.733481 |
|  | rs885944 | 6 | 29709794 | 0.006902 | 0.053691 | 0.964461 | 7.02E-08 | 0.032121 | 0.70161 |
|  | rs929158 | 6 | 29706900 | 0.005833 | 0.062468 | 0.365444 | 7.28E-08 | 0.031035 | 0.79555 |
|  | rs929159 | 6 | 29706889 | 0.005835 | 0.06245 | 0.427894 | 7.28E-08 | 0.031035 | 0.764515 |
|  | rs929160 | 6 | 29706793 | 0.005841 | 0.062395 | 0.49029 | 7.28E-08 | 0.03101 | 0.888616 |
| rs1979377 | rs10425556 | 19 | 45259871 | 1.33E-10 | 0.491173 | 0.491173 | 0.01019 | 0.087226 | 0.852055 |
|  | rs1979377 | 19 | 45259002 | 4.56E-10 | 0.14749 | 0.895703 | 0.004587 | 0.178671 | 0.358909 |
|  | rs2927455 | 19 | 45267258 | 2.58E-10 | 0.257039 | 0.748213 | 0.0134 | 0.068413 | 0.920469 |
|  | rs2965133 | 19 | 45265435 | 7.67E-10 | 0.088705 | 0.984408 | 0.004543 | 0.180238 | 0.180238 |
|  | rs2965143 | 19 | 45279340 | 5.58E-09 | 0.012839 | 0.997247 | 0.008229 | 0.10556 | 0.76483 |
| rs2310752 | rs11208779 | 1 | 66417145 | 0.000463 | 0.031708 | 0.764523 | 3.61E-06 | 0.002851 | 0.992125 |
|  | rs1937433 | 1 | 66444183 | 0.000648 | 0.023153 | 0.866316 | 1.04E-06 | 0.009438 | 0.962446 |
|  | rs1937443 | 1 | 66469643 | 0.00134 | 0.011812 | 0.981569 | 3.66E-08 | 0.2389 | 0.492667 |
|  | rs1937450 | 1 | 66478840 | 0.000974 | 0.015865 | 0.957336 | 6.94E-07 | 0.013895 | 0.921833 |
|  | rs1937455 | 1 | 66416939 | 0.000469 | 0.031308 | 0.795831 | 2.62E-07 | 0.035604 | 0.872626 |
|  | rs2186120 | 1 | 66453163 | 0.000626 | 0.023921 | 0.819752 | 5.48E-07 | 0.017445 | 0.907937 |
|  | rs2186122 | 1 | 66470206 | 0.001269 | 0.012421 | 0.969757 | 4.32E-08 | 0.203483 | 0.69615 |
|  | rs2310752 | 1 | 66392405 | 0.000137 | 0.09958 | 0.09958 | 5.35E-07 | 0.017866 | 0.890492 |
|  | rs2310819 | 1 | 66440096 | 0.000778 | 0.019551 | 0.905929 | 9.84E-07 | 0.009932 | 0.953008 |
|  | rs2503185 | 1 | 66461401 | 0.00092 | 0.016726 | 0.94147 | 8.79E-07 | 0.011067 | 0.932899 |
|  | rs2997084 | 1 | 66393732 | 0.000141 | 0.096521 | 0.196101 | 9.59E-07 | 0.010176 | 0.943076 |
|  | rs3009872 | 1 | 66411400 | 0.000178 | 0.077625 | 0.273726 | 1.25E-06 | 0.007867 | 0.978304 |
|  | rs6421482 | 1 | 66419905 | 0.000756 | 0.020063 | 0.886379 | 1.11E-07 | 0.081731 | 0.777881 |
|  | rs6690398 | 1 | 66447394 | 0.000811 | 0.018815 | 0.924744 | 3.44E-08 | 0.253767 | 0.253767 |
|  | rs7519259 | 1 | 66434743 | 0.001577 | 0.010169 | 0.991738 | 2.44E-06 | 0.004143 | 0.989275 |
|  | rs7528604 | 1 | 66407352 | 0.000181 | 0.076299 | 0.350025 | 1.45E-06 | 0.006828 | 0.985131 |
|  | rs7547416 | 1 | 66419087 | 0.000641 | 0.023411 | 0.843163 | 1.55E-07 | 0.059141 | 0.837022 |
| rs3121427 | rs11186957 | 10 | 94139014 | 0.000139 | 0.027545 | 0.63999 | 1.03E-05 | 0.019529 | 0.907858 |
|  | rs11186958 | 10 | 94139274 | 0.000136 | 0.028104 | 0.584808 | 1.27E-05 | 0.015943 | 0.941625 |
|  | rs11186959 | 10 | 94140831 | 0.000152 | 0.025365 | 0.744033 | 1.32E-05 | 0.015377 | 0.957002 |
|  | rs11186960 | 10 | 94142607 | 0.000202 | 0.019374 | 0.919815 | 1.74E-05 | 0.011818 | 0.994204 |
|  | rs11186965 | 10 | 94143306 | 0.000207 | 0.01897 | 0.938784 | 8.37E-06 | 0.023712 | 0.844896 |
|  | rs11186966 | 10 | 94143315 | 0.000188 | 0.020779 | 0.859957 | 7.86E-06 | 0.025198 | 0.674714 |
|  | rs11186967 | 10 | 94143848 | 0.000194 | 0.020117 | 0.90044 | 7.68E-06 | 0.025746 | 0.624211 |
|  | rs11186968 | 10 | 94144131 | 0.000168 | 0.023049 | 0.816233 | 8.26E-06 | 0.024019 | 0.797353 |
|  | rs11186973 | 10 | 94157411 | 7.93E-05 | 0.046848 | 0.098244 | 4.94E-06 | 0.039295 | 0.241498 |
|  | rs1810809 | 10 | 94146088 | 0.000268 | 0.014896 | 0.971985 | 9.95E-06 | 0.020102 | 0.888329 |
|  | rs1810810 | 10 | 94146243 | 0.000149 | 0.025785 | 0.692979 | 8.16E-06 | 0.024303 | 0.773334 |
|  | rs2019640 | 10 | 94146113 | 0.000298 | 0.013467 | 0.985452 | 1.13E-05 | 0.017824 | 0.925683 |
|  | rs2257625 | 10 | 94145450 | 0.000192 | 0.020367 | 0.880324 | 7.32E-06 | 0.026969 | 0.545135 |
|  | rs2259053 | 10 | 94155360 | 9.92E-05 | 0.037917 | 0.216832 | 5.51E-06 | 0.0354 | 0.427864 |
|  | rs2259090 | 10 | 94154893 | 9.63E-05 | 0.038998 | 0.178915 | 5.11E-06 | 0.038057 | 0.318228 |
|  | rs2259373 | 10 | 94148764 | 0.000102 | 0.036908 | 0.25374 | 7.07E-06 | 0.02787 | 0.518165 |
|  | rs2259430 | 10 | 94147345 | 0.000215 | 0.018305 | 0.957089 | 8.33E-06 | 0.023831 | 0.821185 |
|  | rs2259433 | 10 | 94147300 | 0.000169 | 0.022945 | 0.839178 | 8.52E-06 | 0.023331 | 0.868227 |
|  | rs2265199 | 10 | 94147632 | 0.000162 | 0.023887 | 0.793184 | 8E-06 | 0.024782 | 0.724476 |
|  | rs2265200 | 10 | 94150707 | 0.00015 | 0.025689 | 0.718668 | 6.91E-06 | 0.028482 | 0.490296 |
|  | rs2480302 | 10 | 94157060 | 8.97E-05 | 0.041674 | 0.139917 | 5.3E-06 | 0.036735 | 0.392464 |
|  | rs2480306 | 10 | 94153247 | 0.000121 | 0.031535 | 0.4938 | 4.8E-06 | 0.040409 | 0.122301 |
|  | rs2480307 | 10 | 94153313 | 0.000121 | 0.031457 | 0.525257 | 4.88E-06 | 0.039781 | 0.202203 |
|  | rs2480308 | 10 | 94153319 | 0.000121 | 0.031446 | 0.556703 | 4.83E-06 | 0.04012 | 0.162421 |
|  | rs2771266 | 10 | 94148246 | 0.000153 | 0.025264 | 0.769297 | 8.07E-06 | 0.024556 | 0.749032 |
|  | rs2771268 | 10 | 94155702 | 0.000107 | 0.035356 | 0.360196 | 5.75E-06 | 0.03395 | 0.461813 |
|  | rs2987829 | 10 | 94153688 | 0.000106 | 0.035512 | 0.32484 | 5.19E-06 | 0.037501 | 0.355729 |
|  | rs2995786 | 10 | 94153814 | 0.000106 | 0.035588 | 0.289328 | 5.02E-06 | 0.038673 | 0.280171 |
|  | rs3121424 | 10 | 94149034 | 0.000112 | 0.033946 | 0.428671 | 7.42E-06 | 0.026632 | 0.598464 |
|  | rs3121426 | 10 | 94153435 | 0.000113 | 0.033594 | 0.462265 | 4.7E-06 | 0.041216 | 0.041216 |
|  | rs3121427 | 10 | 94159701 | 7.19E-05 | 0.051396 | 0.051396 | 4.76E-06 | 0.040677 | 0.081892 |
|  | rs7911784 | 10 | 94139488 | 0.00011 | 0.034529 | 0.394725 | 1.63E-05 | 0.012535 | 0.982386 |
|  | rs7912223 | 10 | 94139842 | 0.000139 | 0.027638 | 0.612445 | 1.59E-05 | 0.01285 | 0.969851 |
|  | rs913645 | 10 | 94146032 | 0.000141 | 0.027205 | 0.667195 | 7.93E-06 | 0.024979 | 0.699694 |
| rs359539 | rs358900 | 3 | 1.55E+08 | 2.3E-07 | 0.083685 | 0.843548 | 0.00427 | 0.029909 | 0.982901 |
|  | rs359538 | 3 | 1.55E+08 | 6.98E-07 | 0.028648 | 0.945681 | 0.00325 | 0.038332 | 0.85157 |
|  | rs359539 | 3 | 1.55E+08 | 1.15E-07 | 0.164059 | 0.164059 | 0.001042 | 0.108986 | 0.341252 |
|  | rs359556 | 3 | 1.55E+08 | 1.6E-06 | 0.012935 | 0.993878 | 0.01675 | 0.008817 | 0.991718 |
|  | rs359561 | 3 | 1.55E+08 | 1.72E-07 | 0.11092 | 0.274979 | 0.000989 | 0.114403 | 0.232265 |
|  | rs359562 | 3 | 1.55E+08 | 1.83E-07 | 0.104593 | 0.485845 | 0.001057 | 0.107555 | 0.448806 |
|  | rs359564 | 3 | 1.55E+08 | 1.8E-07 | 0.106273 | 0.381252 | 0.000958 | 0.117862 | 0.117862 |
|  | rs359566 | 3 | 1.55E+08 | 1.86E-07 | 0.102884 | 0.588729 | 0.001179 | 0.097222 | 0.546029 |
|  | rs359568 | 3 | 1.55E+08 | 2.23E-07 | 0.086254 | 0.674983 | 0.001228 | 0.093633 | 0.639662 |
|  | rs359569 | 3 | 1.55E+08 | 1.25E-06 | 0.016388 | 0.980943 | 0.003962 | 0.032011 | 0.952992 |
|  | rs359570 | 3 | 1.55E+08 | 2.63E-07 | 0.073485 | 0.917033 | 0.001366 | 0.084867 | 0.813238 |
|  | rs359572 | 3 | 1.55E+08 | 2.27E-07 | 0.08488 | 0.759863 | 0.001302 | 0.088709 | 0.728371 |
| rs4727449 | rs13236234 | 7 | 99963018 | 1.28E-07 | 0.110571 | 0.654203 | 0.002488 | 0.121981 | 0.377999 |
|  | rs13246354 | 7 | 99950006 | 1.06E-07 | 0.132554 | 0.543632 | 0.002427 | 0.124783 | 0.256018 |
|  | rs34183059 | 7 | 1E+08 | 1.47E-06 | 0.010465 | 1 | 0.005853 | 0.056074 | 1 |
|  | rs34893607 | 7 | 99946207 | 2.67E-07 | 0.054143 | 0.925031 | 0.003015 | 0.102345 | 0.719372 |
|  | rs35146386 | 7 | 1E+08 | 1.59E-07 | 0.089214 | 0.743417 | 0.004639 | 0.069179 | 0.875389 |
|  | rs36033613 | 7 | 99921243 | 2.03E-07 | 0.070546 | 0.813963 | 0.003611 | 0.086838 | 0.80621 |
|  | rs3807475 | 7 | 1E+08 | 3.86E-07 | 0.037916 | 0.962946 | 0.002499 | 0.12149 | 0.499489 |
|  | rs4727449 | 7 | 99785750 | 3.29E-08 | 0.411078 | 0.411078 | 0.002591 | 0.117538 | 0.617027 |
|  | rs4727455 | 7 | 1E+08 | 2.53E-07 | 0.056924 | 0.870887 | 0.004687 | 0.068537 | 0.943926 |
|  | rs5015755 | 7 | 1E+08 | 5.58E-07 | 0.026589 | 0.989535 | 0.002297 | 0.131235 | 0.131235 |
| rs56249331 | rs10914243 | 1 | 31487513 | 0.001687 | 0.026037 | 0.392002 | 3.03E-07 | 0.011086 | 0.952792 |
|  | rs10914251 | 1 | 31510263 | 0.003166 | 0.014628 | 0.921188 | 1.9E-07 | 0.017412 | 0.748067 |
|  | rs10914267 | 1 | 31568793 | 0.00191 | 0.023228 | 0.614406 | 9.58E-08 | 0.033725 | 0.485343 |
|  | rs10914269 | 1 | 31569231 | 0.00182 | 0.024286 | 0.567427 | 1.11E-07 | 0.029137 | 0.51448 |
|  | rs12066225 | 1 | 31526251 | 0.002144 | 0.020889 | 0.700733 | 2.01E-07 | 0.016497 | 0.781896 |
|  | rs1539361 | 1 | 31444200 | 0.001621 | 0.027012 | 0.365965 | 2.99E-07 | 0.01123 | 0.941706 |
|  | rs1999810 | 1 | 31464422 | 0.003557 | 0.013156 | 0.948631 | 6.94E-08 | 0.046066 | 0.240708 |
|  | rs1999811 | 1 | 31464327 | 0.002318 | 0.019448 | 0.800804 | 6.87E-08 | 0.046507 | 0.148253 |
|  | rs2153850 | 1 | 31421324 | 0.001768 | 0.024935 | 0.518547 | 2.26E-07 | 0.014677 | 0.84425 |
|  | rs2491135 | 1 | 31445932 | 0.001485 | 0.02928 | 0.282662 | 3.66E-07 | 0.009234 | 0.982255 |
|  | rs4576693 | 1 | 31544006 | 0.001566 | 0.027876 | 0.338953 | 1.8E-07 | 0.018279 | 0.65981 |
|  | rs4607940 | 1 | 31491550 | 0.001759 | 0.025053 | 0.493612 | 2.91E-07 | 0.011524 | 0.907697 |
|  | rs4642942 | 1 | 31492150 | 0.003591 | 0.013044 | 0.961675 | 3.07E-08 | 0.101745 | 0.101745 |
|  | rs4949186 | 1 | 31402148 | 0.002075 | 0.021531 | 0.679844 | 2.64E-07 | 0.012643 | 0.883902 |
|  | rs4949191 | 1 | 31513035 | 0.002884 | 0.015927 | 0.90656 | 1.64E-07 | 0.020008 | 0.584422 |
|  | rs4949192 | 1 | 31567131 | 0.001864 | 0.023752 | 0.591178 | 7.01E-08 | 0.045658 | 0.286366 |
|  | rs4949329 | 1 | 31440361 | 0.001795 | 0.024593 | 0.54314 | 3.38E-07 | 0.009964 | 0.973021 |
|  | rs4949332 | 1 | 31503691 | 0.006565 | 0.007556 | 0.986545 | 2.58E-07 | 0.012922 | 0.87126 |
|  | rs4949334 | 1 | 31511383 | 0.002215 | 0.020278 | 0.74179 | 1.72E-07 | 0.019099 | 0.622902 |
|  | rs4949340 | 1 | 31562297 | 0.002156 | 0.02078 | 0.721512 | 7.43E-08 | 0.04312 | 0.373246 |
|  | rs56249331 | 1 | 31393417 | 0.001024 | 0.041256 | 0.119644 | 1.38E-07 | 0.023768 | 0.564414 |
|  | rs56389855 | 1 | 31540394 | 0.001708 | 0.025746 | 0.417748 | 2.36E-07 | 0.014088 | 0.858338 |
|  | rs61780504 | 1 | 31522834 | 0.002059 | 0.021682 | 0.658313 | 2.01E-07 | 0.016457 | 0.798354 |
|  | rs6425694 | 1 | 31491210 | 0.005842 | 0.008394 | 0.978988 | 2.91E-07 | 0.011513 | 0.91921 |
|  | rs6425699 | 1 | 31520915 | 0.002004 | 0.022225 | 0.636631 | 1.83E-07 | 0.018057 | 0.677867 |
|  | rs6656007 | 1 | 31504963 | 0.003249 | 0.014287 | 0.935475 | 1.91E-07 | 0.017333 | 0.7654 |
|  | rs6656963 | 1 | 31459054 | 0.001534 | 0.028415 | 0.311078 | 3.68E-07 | 0.009176 | 0.991432 |
|  | rs6667620 | 1 | 31557320 | 0.002685 | 0.017005 | 0.890633 | 7.32E-08 | 0.043759 | 0.330125 |
|  | rs6668198 | 1 | 31517020 | 0.002266 | 0.019858 | 0.761649 | 2.07E-07 | 0.016011 | 0.814365 |
|  | rs6668319 | 1 | 31487097 | 0.001336 | 0.032279 | 0.253382 | 2.73E-07 | 0.012271 | 0.896173 |
|  | rs6669623 | 1 | 31413648 | 0.001314 | 0.032775 | 0.221103 | 1.25E-07 | 0.026165 | 0.540646 |
|  | rs6685634 | 1 | 31542705 | 0.007021 | 0.007114 | 0.993659 | 1.77E-07 | 0.018629 | 0.641531 |
|  | rs6688087 | 1 | 31542579 | 0.005462 | 0.008919 | 0.970594 | 1.89E-07 | 0.017475 | 0.730655 |
|  | rs6698832 | 1 | 31518461 | 0.002454 | 0.018459 | 0.838438 | 1.89E-07 | 0.017492 | 0.71318 |
|  | rs72887507 | 1 | 31550527 | 0.002354 | 0.019175 | 0.819979 | 8.9E-08 | 0.036229 | 0.451618 |
|  | rs7527893 | 1 | 31493479 | 0.001748 | 0.025199 | 0.468559 | 2.18E-07 | 0.015209 | 0.829574 |
|  | rs7537241 | 1 | 31511004 | 0.002604 | 0.017487 | 0.873628 | 1.7E-07 | 0.019381 | 0.603804 |
|  | rs7544112 | 1 | 31403711 | 0.001303 | 0.033031 | 0.188328 | 3.28E-07 | 0.010266 | 0.963057 |
|  | rs945696 | 1 | 31566345 | 0.001717 | 0.025612 | 0.44336 | 7.61E-08 | 0.042143 | 0.415389 |
|  | rs955809 | 1 | 31535557 | 0.002569 | 0.017703 | 0.856141 | 1.85E-07 | 0.017821 | 0.695688 |
|  | rs9661612 | 1 | 31428852 | 0.0012 | 0.035652 | 0.155297 | 2.98E-07 | 0.011266 | 0.930476 |
| rs858502 | rs10953295 | 7 | 99791539 | 4.33E-08 | 0.012924 | 0.961959 | 0.007898 | 0.043453 | 0.860369 |
|  | rs12705073 | 7 | 99774122 | 3.89E-09 | 0.134257 | 0.801377 | 0.01161 | 0.030821 | 1 |
|  | rs1727145 | 7 | 99800835 | 6.15E-08 | 0.009189 | 0.983985 | 0.005459 | 0.060571 | 0.535649 |
|  | rs1859787 | 7 | 99824353 | 3.22E-08 | 0.017219 | 0.916748 | 0.004302 | 0.075137 | 0.075137 |
|  | rs2246713 | 7 | 99802812 | 3.31E-08 | 0.016758 | 0.933507 | 0.005779 | 0.057537 | 0.770835 |
|  | rs2906646 | 7 | 99818137 | 2.48E-08 | 0.022161 | 0.881173 | 0.004836 | 0.067581 | 0.215854 |
|  | rs2950518 | 7 | 99807344 | 2.07E-08 | 0.026484 | 0.859013 | 0.007397 | 0.046082 | 0.816916 |
|  | rs3735241 | 7 | 99796146 | 6.21E-08 | 0.009116 | 0.993101 | 0.005558 | 0.059596 | 0.655418 |
|  | rs4299940 | 7 | 99793942 | 3.01E-08 | 0.018356 | 0.899529 | 0.005741 | 0.05788 | 0.713298 |
|  | rs4729580 | 7 | 99789817 | 3.52E-09 | 0.147971 | 0.667121 | 0.01092 | 0.032544 | 0.969179 |
|  | rs705867 | 7 | 99818927 | 1.75E-08 | 0.031151 | 0.832529 | 0.005008 | 0.065478 | 0.347468 |
|  | rs7799441 | 7 | 99776272 | 1.7E-09 | 0.300377 | 0.300377 | 0.009315 | 0.037493 | 0.936636 |
|  | rs858502 | 7 | 99843353 | 2.36E-09 | 0.218772 | 0.51915 | 0.004432 | 0.073136 | 0.148273 |
|  | rs858510 | 7 | 99821894 | 3.58E-08 | 0.015528 | 0.949035 | 0.004953 | 0.066135 | 0.28199 |
|  | rs941287 | 7 | 99807473 | 4.36E-08 | 0.012837 | 0.974796 | 0.005499 | 0.060173 | 0.595822 |

## Supplementary Table8 Fine-mapping 99% credible-set of sentinel SNP from cross-trait meta-analysis between Alzheimer's disease (AD) and snoring.

| Sentinel.SNP | Credible-set SNPs | CHR | BP | Alzheimer's disease | | | snoring | | |
| --- | --- | --- | --- | --- | --- | --- | --- | --- | --- |
|  |  |  |  | **GWAS-p-value** | **probNorm** | **cumSum** | **GWAS-p-value** | **probNorm** | **cumSum** |
| rs1004173 | rs1004173 | 6 | 47445017 | 5.64E-09 | 0.023509 | 0.075601 | 0.005916 | 0.014884 | 0.339171 |
|  | rs10456570 | 6 | 47559451 | 1.04E-08 | 0.012936 | 0.633771 | 0.007562 | 0.011936 | 0.805503 |
|  | rs10948363 | 6 | 47487762 | 8.9E-09 | 0.015075 | 0.261658 | 0.005747 | 0.015278 | 0.279392 |
|  | rs10948367 | 6 | 47585615 | 1.56E-08 | 0.008718 | 0.94771 | 0.00807 | 0.011261 | 0.934074 |
|  | rs12193051 | 6 | 47541415 | 1.07E-08 | 0.01264 | 0.710359 | 0.007592 | 0.011894 | 0.82933 |
|  | rs12195738 | 6 | 47583664 | 1.78E-08 | 0.007701 | 0.971487 | 0.006145 | 0.014383 | 0.484746 |
|  | rs13193054 | 6 | 47552817 | 9.37E-09 | 0.014347 | 0.378431 | 0.007437 | 0.012116 | 0.733435 |
|  | rs13201473 | 6 | 47489708 | 1.16E-08 | 0.011606 | 0.82023 | 0.005936 | 0.014838 | 0.354009 |
|  | rs13212790 | 6 | 47576367 | 9.84E-09 | 0.013673 | 0.447627 | 0.007115 | 0.012607 | 0.658597 |
|  | rs1485780 | 6 | 47556630 | 8.56E-09 | 0.015656 | 0.231136 | 0.007647 | 0.011817 | 0.841148 |
|  | rs1872505 | 6 | 47480975 | 1.01E-08 | 0.01333 | 0.542008 | 0.005483 | 0.01594 | 0.171497 |
|  | rs1931837 | 6 | 47442377 | 5.42E-09 | 0.024433 | 0.052092 | 0.007479 | 0.012055 | 0.74549 |
|  | rs1948047 | 6 | 47585912 | 1.28E-08 | 0.010614 | 0.863779 | 0.009238 | 0.009979 | 0.976622 |
|  | rs2151974 | 6 | 47515630 | 1.06E-08 | 0.012763 | 0.685076 | 0.006659 | 0.01338 | 0.595018 |
|  | rs2151975 | 6 | 47515663 | 9.18E-09 | 0.014629 | 0.320564 | 0.006576 | 0.013532 | 0.568141 |
|  | rs2171086 | 6 | 47590104 | 1.49E-08 | 0.009163 | 0.921124 | 0.008411 | 0.010851 | 0.956139 |
|  | rs2171089 | 6 | 47515811 | 8.26E-09 | 0.016208 | 0.183792 | 0.006413 | 0.013841 | 0.554609 |
|  | rs2396825 | 6 | 47568696 | 9.91E-09 | 0.013582 | 0.461209 | 0.007496 | 0.012031 | 0.769573 |
|  | rs34744382 | 6 | 47536444 | 1.03E-08 | 0.013029 | 0.620835 | 0.00801 | 0.011336 | 0.922813 |
|  | rs4711878 | 6 | 47456118 | 1.13E-08 | 0.011984 | 0.796821 | 0.005738 | 0.015299 | 0.248818 |
|  | rs4711880 | 6 | 47480676 | 8.11E-09 | 0.016513 | 0.167583 | 0.006033 | 0.014623 | 0.398077 |
|  | rs4715018 | 6 | 47441871 | 5.91E-09 | 0.022444 | 0.098045 | 0.007181 | 0.012503 | 0.721319 |
|  | rs4715019 | 6 | 47447041 | 9.19E-09 | 0.014608 | 0.335172 | 0.006595 | 0.013497 | 0.581638 |
|  | rs4715025 | 6 | 47483653 | 8.37E-09 | 0.016007 | 0.199799 | 0.005975 | 0.014751 | 0.36876 |
|  | rs6903331 | 6 | 47562915 | 9.31E-09 | 0.014431 | 0.364084 | 0.00751 | 0.01201 | 0.781583 |
|  | rs6904764 | 6 | 47503497 | 9.96E-09 | 0.013521 | 0.488309 | 0.006118 | 0.01444 | 0.455978 |
|  | rs6913202 | 6 | 47489104 | 9.06E-09 | 0.014823 | 0.27648 | 0.005911 | 0.014895 | 0.324287 |
|  | rs6931011 | 6 | 47525202 | 1.66E-08 | 0.008227 | 0.955938 | 0.005055 | 0.017154 | 0.072418 |
|  | rs6931478 | 6 | 47461913 | 1.02E-08 | 0.01316 | 0.581603 | 0.004669 | 0.018432 | 0.037429 |
|  | rs7738044 | 6 | 47469273 | 1.05E-08 | 0.012785 | 0.672312 | 0.005441 | 0.016051 | 0.155557 |
|  | rs7749167 | 6 | 47493940 | 1.4E-08 | 0.009723 | 0.883723 | 0.005739 | 0.015297 | 0.264114 |
|  | rs7749271 | 6 | 47485002 | 1.09E-08 | 0.012363 | 0.76041 | 0.005116 | 0.016969 | 0.106404 |
|  | rs7754282 | 6 | 47502024 | 9.42E-09 | 0.014262 | 0.392693 | 0.006089 | 0.014502 | 0.412579 |
|  | rs7754971 | 6 | 47590476 | 1.23E-08 | 0.011009 | 0.831239 | 0.008108 | 0.011214 | 0.945288 |
|  | rs7767350 | 6 | 47485126 | 7.78E-09 | 0.01718 | 0.151071 | 0.006173 | 0.014324 | 0.49907 |
|  | rs7774667 | 6 | 47514758 | 9.28E-09 | 0.014481 | 0.349653 | 0.00639 | 0.013886 | 0.526916 |
|  | rs901186 | 6 | 47514246 | 2.01E-08 | 0.006846 | 0.993397 | 0.006407 | 0.013852 | 0.540768 |
|  | rs9296558 | 6 | 47451883 | 9.7E-09 | 0.013864 | 0.406558 | 0.007139 | 0.012569 | 0.683747 |
|  | rs9296559 | 6 | 47452270 | 1.02E-08 | 0.01325 | 0.555258 | 0.007181 | 0.012503 | 0.708816 |
|  | rs9296561 | 6 | 47488938 | 1.08E-08 | 0.012537 | 0.73551 | 0.005264 | 0.016537 | 0.139507 |
|  | rs9296564 | 6 | 47494759 | 8.68E-09 | 0.015446 | 0.246583 | 0.006105 | 0.014468 | 0.441538 |
|  | rs9296567 | 6 | 47553402 | 1.51E-08 | 0.009007 | 0.930131 | 0.005737 | 0.015302 | 0.233518 |
|  | rs9349409 | 6 | 47463126 | 1.11E-08 | 0.012138 | 0.784837 | 0.0051 | 0.017017 | 0.089435 |
|  | rs9349413 | 6 | 47511491 | 1.74E-08 | 0.007848 | 0.963786 | 0.004516 | 0.018997 | 0.018997 |
|  | rs9349415 | 6 | 47551861 | 1.05E-08 | 0.012879 | 0.646651 | 0.007648 | 0.011816 | 0.864781 |
|  | rs9349416 | 6 | 47551938 | 1.05E-08 | 0.012877 | 0.659528 | 0.007648 | 0.011816 | 0.876597 |
|  | rs9349417 | 6 | 47580657 | 1.14E-08 | 0.011803 | 0.808624 | 0.007675 | 0.011779 | 0.888375 |
|  | rs9357546 | 6 | 47549495 | 1E-08 | 0.013428 | 0.528678 | 0.007141 | 0.012566 | 0.696313 |
|  | rs9367279 | 6 | 47448336 | 1.02E-08 | 0.013158 | 0.594761 | 0.006885 | 0.012985 | 0.608002 |
|  | rs9367284 | 6 | 47516369 | 9.12E-09 | 0.014728 | 0.291209 | 0.005888 | 0.014947 | 0.309393 |
|  | rs9369693 | 6 | 47433751 | 1.03E-08 | 0.013046 | 0.607807 | 0.01716 | 0.005768 | 0.98239 |
|  | rs9369695 | 6 | 47440565 | 4.77E-09 | 0.027659 | 0.027659 | 0.008723 | 0.010504 | 0.966643 |
|  | rs9369716 | 6 | 47552180 | 8.55E-09 | 0.015682 | 0.21548 | 0.007481 | 0.012052 | 0.757542 |
|  | rs9369717 | 6 | 47554468 | 1.79E-08 | 0.007642 | 0.97913 | 0.00551 | 0.015869 | 0.187366 |
|  | rs9381562 | 6 | 47429767 | 7.56E-09 | 0.017667 | 0.133891 | 0.02456 | 0.00422 | 0.991749 |
|  | rs9381564 | 6 | 47443806 | 7.34E-09 | 0.018178 | 0.116223 | 0.006352 | 0.01396 | 0.513031 |
|  | rs9381575 | 6 | 47528764 | 9.96E-09 | 0.013511 | 0.50182 | 0.006094 | 0.014491 | 0.42707 |
|  | rs9381578 | 6 | 47556634 | 9.82E-09 | 0.013709 | 0.420267 | 0.007647 | 0.011817 | 0.852965 |
|  | rs9381579 | 6 | 47556680 | 1.46E-08 | 0.009339 | 0.902631 | 0.007564 | 0.011934 | 0.817436 |
|  | rs9381581 | 6 | 47580695 | 9.91E-09 | 0.013579 | 0.474788 | 0.00777 | 0.01165 | 0.900025 |
|  | rs9395262 | 6 | 47465267 | 1.1E-08 | 0.012289 | 0.772699 | 0.005254 | 0.016566 | 0.122969 |
|  | rs9395279 | 6 | 47542864 | 1.08E-08 | 0.012537 | 0.748047 | 0.007131 | 0.012582 | 0.671178 |
|  | rs9395283 | 6 | 47551444 | 1.07E-08 | 0.012643 | 0.697719 | 0.007529 | 0.011983 | 0.793566 |
|  | rs9395285 | 6 | 47554177 | 1.85E-08 | 0.007422 | 0.986551 | 0.005679 | 0.015442 | 0.202809 |
|  | rs9395286 | 6 | 47575332 | 1.23E-08 | 0.010988 | 0.842227 | 0.007087 | 0.012652 | 0.633351 |
|  | rs9463335 | 6 | 47479136 | 1.46E-08 | 0.00933 | 0.911961 | 0.004842 | 0.017835 | 0.055264 |
|  | rs9463342 | 6 | 47585106 | 1.42E-08 | 0.009568 | 0.893291 | 0.00792 | 0.011452 | 0.911477 |
|  | rs9473117 | 6 | 47431284 | 1.24E-08 | 0.010938 | 0.853165 | 0.01958 | 0.005139 | 0.98753 |
|  | rs9473119 | 6 | 47450618 | 1.02E-08 | 0.013184 | 0.568442 | 0.007095 | 0.012639 | 0.64599 |
|  | rs9473122 | 6 | 47474962 | 1E-08 | 0.013429 | 0.515249 | 0.005693 | 0.015408 | 0.218217 |
|  | rs9473123 | 6 | 47475339 | 1.07E-08 | 0.012613 | 0.722972 | 0.005842 | 0.015053 | 0.294445 |
|  | rs9473126 | 6 | 47481833 | 9.12E-09 | 0.014727 | 0.305935 | 0.006001 | 0.014693 | 0.383454 |
|  | rs9473128 | 6 | 47505010 | 9.83E-09 | 0.013687 | 0.433954 | 0.006144 | 0.014385 | 0.470363 |
| rs11100203 | rs10434104 | 4 | 1.6E+08 | 4.12E-05 | 0.066525 | 0.863211 | 0.000118 | 0.078905 | 0.869232 |
|  | rs10857319 | 4 | 1.6E+08 | 8.39E-05 | 0.033932 | 0.969703 | 0.000122 | 0.076516 | 0.945748 |
|  | rs11100200 | 4 | 1.6E+08 | 7.91E-05 | 0.035865 | 0.935771 | 0.000175 | 0.054252 | 1 |
|  | rs11100203 | 4 | 1.6E+08 | 4.13E-06 | 0.596473 | 0.596473 | 2.93E-05 | 0.294418 | 0.616603 |
|  | rs2114489 | 4 | 1.6E+08 | 1.29E-05 | 0.200213 | 0.796687 | 2.67E-05 | 0.322186 | 0.322186 |
|  | rs4234926 | 4 | 1.6E+08 | 7.72E-05 | 0.036695 | 0.899906 | 0.000114 | 0.081452 | 0.790328 |
|  | rs6830034 | 4 | 1.6E+08 | 9.48E-05 | 0.03021 | 0.999914 | 9.97E-05 | 0.092272 | 0.708875 |
| rs11642303 | rs1108431 | 16 | 31054607 | 6.27E-05 | 0.009187 | 0.944141 | 1.35E-09 | 0.042111 | 0.084344 |
|  | rs112906665 | 16 | 31040139 | 3.38E-05 | 0.016524 | 0.64158 | 2.48E-09 | 0.023309 | 0.677416 |
|  | rs11640767 | 16 | 31060647 | 3.99E-05 | 0.014116 | 0.760276 | 1.63E-09 | 0.035175 | 0.238592 |
|  | rs11640957 | 16 | 31060363 | 4.44E-05 | 0.012749 | 0.853746 | 1.83E-09 | 0.031346 | 0.302662 |
|  | rs11642003 | 16 | 31068391 | 7.27E-06 | 0.071483 | 0.071483 | 2.2E-09 | 0.026231 | 0.60234 |
|  | rs11642192 | 16 | 31082025 | 1.94E-05 | 0.02797 | 0.408496 | 6.15E-09 | 0.009631 | 0.906097 |
|  | rs11642303 | 16 | 31068392 | 7.71E-06 | 0.067561 | 0.139044 | 2.04E-09 | 0.028129 | 0.388176 |
|  | rs11647284 | 16 | 31077335 | 2.85E-05 | 0.019415 | 0.606536 | 7.25E-09 | 0.008203 | 0.97527 |
|  | rs11862744 | 16 | 31057800 | 3.7E-05 | 0.01516 | 0.68841 | 2.16E-09 | 0.02662 | 0.549596 |
|  | rs11864839 | 16 | 31095251 | 1.46E-05 | 0.036822 | 0.292797 | 7.01E-09 | 0.008475 | 0.94213 |
|  | rs11865038 | 16 | 31095171 | 1.31E-05 | 0.040661 | 0.179705 | 6.25E-09 | 0.00947 | 0.915567 |
|  | rs12445650 | 16 | 31044897 | 5.18E-05 | 0.011015 | 0.925245 | 2.16E-09 | 0.026632 | 0.496355 |
|  | rs12447930 | 16 | 31023273 | 4.2E-05 | 0.013425 | 0.801604 | 2.71E-09 | 0.021342 | 0.74246 |
|  | rs17839567 | 16 | 31057945 | 4.02E-05 | 0.014023 | 0.774299 | 2.16E-09 | 0.02662 | 0.522975 |
|  | rs17839568 | 16 | 31099783 | 1.94E-05 | 0.027937 | 0.436433 | 5.98E-09 | 0.009889 | 0.877159 |
|  | rs2199036 | 16 | 31006972 | 3.88E-05 | 0.014504 | 0.717426 | 5.06E-09 | 0.011629 | 0.847122 |
|  | rs28814987 | 16 | 31081460 | 3E-05 | 0.018519 | 0.625056 | 3.79E-09 | 0.015418 | 0.798617 |
|  | rs34454770 | 16 | 31005567 | 6.57E-05 | 0.008795 | 0.961754 | 7.16E-09 | 0.008301 | 0.958854 |
|  | rs35468353 | 16 | 31056433 | 4.21E-05 | 0.013416 | 0.81502 | 1.99E-09 | 0.028943 | 0.331606 |
|  | rs35713203 | 16 | 31090407 | 2.29E-05 | 0.02389 | 0.564815 | 1.07E-08 | 0.005629 | 0.994994 |
|  | rs35961830 | 16 | 31032317 | 3.59E-05 | 0.015609 | 0.673251 | 2.25E-09 | 0.025617 | 0.654106 |
|  | rs3751855 | 16 | 31091209 | 1.35E-05 | 0.039429 | 0.219134 | 7.24E-09 | 0.008214 | 0.967067 |
|  | rs4468641 | 16 | 31096876 | 1.88E-05 | 0.028865 | 0.380526 | 5.92E-09 | 0.009996 | 0.86727 |
|  | rs4889526 | 16 | 31030344 | 2.23E-05 | 0.024483 | 0.540925 | 2.84E-09 | 0.020391 | 0.76285 |
|  | rs4889609 | 16 | 31026427 | 3.9E-05 | 0.014402 | 0.731828 | 2.85E-09 | 0.020349 | 0.7832 |
|  | rs55979739 | 16 | 31049155 | 6.55E-05 | 0.008819 | 0.95296 | 1.49E-09 | 0.038202 | 0.203417 |
|  | rs56284083 | 16 | 31052895 | 4.29E-05 | 0.013182 | 0.828202 | 2.14E-09 | 0.026862 | 0.469722 |
|  | rs57434408 | 16 | 31066380 | 4.06E-05 | 0.01388 | 0.788179 | 1.75E-09 | 0.032724 | 0.271316 |
|  | rs57576577 | 16 | 31036367 | 3.92E-05 | 0.014331 | 0.746159 | 2.12E-09 | 0.027184 | 0.44286 |
|  | rs58726213 | 16 | 31044683 | 4.42E-05 | 0.012796 | 0.840998 | 2.09E-09 | 0.0275 | 0.415676 |
|  | rs59061704 | 16 | 31062704 | 4.65E-05 | 0.012213 | 0.891141 | 1.46E-09 | 0.039122 | 0.165215 |
|  | rs6565217 | 16 | 31083324 | 2.19E-05 | 0.024926 | 0.516442 | 6.41E-09 | 0.00925 | 0.924817 |
|  | rs7184567 | 16 | 31021078 | 4.55E-05 | 0.012448 | 0.878928 | 2.61E-09 | 0.022136 | 0.699552 |
|  | rs7196726 | 16 | 31092075 | 1.84E-05 | 0.029379 | 0.351661 | 4.8E-09 | 0.012246 | 0.823401 |
|  | rs7197717 | 16 | 31083075 | 1.95E-05 | 0.027838 | 0.464271 | 6.14E-09 | 0.00964 | 0.896466 |
|  | rs7199949 | 16 | 31096164 | 1.45E-05 | 0.036842 | 0.255976 | 6.72E-09 | 0.008837 | 0.933655 |
|  | rs7203999 | 16 | 31017554 | 4.82E-05 | 0.011791 | 0.902932 | 5.82E-09 | 0.010152 | 0.857274 |
|  | rs72800847 | 16 | 31022639 | 5.04E-05 | 0.011297 | 0.914229 | 4.87E-09 | 0.012092 | 0.835493 |
|  | rs7294 | 16 | 31102321 | 1.84E-05 | 0.029484 | 0.322281 | 6.12E-09 | 0.009667 | 0.886826 |
|  | rs729482 | 16 | 31016970 | 5.92E-05 | 0.009709 | 0.934954 | 7.98E-09 | 0.007475 | 0.982745 |
|  | rs732172 | 16 | 31050033 | 7.75E-05 | 0.007518 | 0.985256 | 1.35E-09 | 0.042233 | 0.042233 |
|  | rs732173 | 16 | 31050023 | 7.23E-05 | 0.00803 | 0.969785 | 1.36E-09 | 0.041749 | 0.126093 |
|  | rs73530203 | 16 | 31099859 | 2.46E-05 | 0.022306 | 0.587122 | 9.04E-09 | 0.006621 | 0.989365 |
|  | rs7500176 | 16 | 31041137 | 7.31E-05 | 0.007953 | 0.977738 | 2.69E-09 | 0.021566 | 0.721118 |
|  | rs750952 | 16 | 31093954 | 2E-05 | 0.027245 | 0.491516 | 4.69E-09 | 0.012538 | 0.811156 |
|  | rs8056842 | 16 | 31047330 | 4.45E-05 | 0.012734 | 0.866481 | 2.02E-09 | 0.028441 | 0.360047 |
|  | rs8061047 | 16 | 31068960 | 3.48E-05 | 0.016062 | 0.657642 | 2.17E-09 | 0.026513 | 0.576109 |
|  | rs9673641 | 16 | 31039376 | 3.87E-05 | 0.014512 | 0.702922 | 2.2E-09 | 0.02615 | 0.628489 |
| rs147188206 | rs147188206 | 19 | 45552587 | 2.62E-14 | 1 | 1 | 0.002893 | 1 | 1 |
| rs204911 | rs190651665 | 19 | 45456103 | 1.06E-09 | 0.153696 | 0.847657 | 0.007915 | 0.200729 | 0.892484 |
|  | rs204906 | 19 | 45461980 | 1.07E-09 | 0.152343 | 1 | 0.007007 | 0.223909 | 0.691755 |
|  | rs204910 | 19 | 45463385 | 6.75E-10 | 0.238008 | 0.481892 | 0.006685 | 0.233576 | 0.467846 |
|  | rs204911 | 19 | 45463540 | 6.58E-10 | 0.243885 | 0.243885 | 0.006663 | 0.23427 | 0.23427 |
|  | rs71352241 | 19 | 45443934 | 7.6E-10 | 0.212068 | 0.693961 | 0.01599 | 0.107516 | 1 |
| rs28469095 | rs10401157 | 19 | 45642044 | 5.21E-38 | 0.077103 | 0.571857 | 0.007344 | 0.052535 | 0.607929 |
|  | rs10401823 | 19 | 45642545 | 8.22E-38 | 0.049053 | 0.868386 | 0.007997 | 0.048673 | 0.75728 |
|  | rs1048699 | 19 | 45650386 | 2.16E-37 | 0.018778 | 0.973967 | 0.008446 | 0.046351 | 0.851836 |
|  | rs1114831 | 19 | 45636319 | 4.7E-37 | 0.008667 | 0.99699 | 0.006779 | 0.056451 | 0.446166 |
|  | rs113321260 | 19 | 45651237 | 3.25E-38 | 0.123335 | 0.494755 | 0.008463 | 0.046268 | 0.898104 |
|  | rs12462040 | 19 | 45612226 | 1.64E-37 | 0.024689 | 0.893075 | 0.00788 | 0.04932 | 0.708607 |
|  | rs17643262 | 19 | 45631816 | 1.89E-37 | 0.02139 | 0.914465 | 0.007109 | 0.054091 | 0.555394 |
|  | rs2004357 | 19 | 45618959 | 7.1E-38 | 0.056737 | 0.76852 | 0.006321 | 0.060116 | 0.271234 |
|  | rs28469095 | 19 | 45655333 | 1.07E-38 | 0.371419 | 0.371419 | 0.003141 | 0.113285 | 0.113285 |
|  | rs28620490 | 19 | 45638245 | 7.93E-38 | 0.050813 | 0.819333 | 0.006455 | 0.058992 | 0.389716 |
|  | rs34545713 | 19 | 45614601 | 1.96E-37 | 0.020692 | 0.935158 | 0.006959 | 0.055137 | 0.501303 |
|  | rs7248421 | 19 | 45606125 | 2.83E-37 | 0.014356 | 0.988323 | 0.009508 | 0.041699 | 0.939803 |
|  | rs74846209 | 19 | 45650099 | 5.62E-38 | 0.071546 | 0.643404 | 0.01012 | 0.039444 | 0.979247 |
|  | rs754366 | 19 | 45633686 | 2.02E-37 | 0.020031 | 0.955189 | 0.008084 | 0.048204 | 0.805485 |
|  | rs78273125 | 19 | 45644354 | 5.88E-38 | 0.068379 | 0.711783 | 0.007532 | 0.051358 | 0.659287 |
| rs3098882 | rs10090230 | 8 | 71797013 | 0.027109 | 0.009829 | 0.939575 | 1.18E-07 | 0.000956 | 0.991125 |
|  | rs10110676 | 8 | 71970809 | 0.089156 | 0.003626 | 0.992183 | 1.16E-09 | 0.085357 | 0.085357 |
|  | rs10429277 | 8 | 71701391 | 0.019522 | 0.013073 | 0.378195 | 9.1E-08 | 0.001232 | 0.968147 |
|  | rs10453108 | 8 | 71782940 | 0.025573 | 0.010338 | 0.858934 | 1.09E-07 | 0.001035 | 0.985208 |
|  | rs11779364 | 8 | 71954596 | 0.016654 | 0.015025 | 0.226101 | 3.88E-09 | 0.02633 | 0.756736 |
|  | rs11989644 | 8 | 71641196 | 0.023771 | 0.011013 | 0.784814 | 9.48E-08 | 0.001184 | 0.974158 |
|  | rs11994908 | 8 | 71721619 | 0.018612 | 0.01363 | 0.324969 | 8.93E-08 | 0.001254 | 0.965683 |
|  | rs12155783 | 8 | 71999126 | 0.035992 | 0.007707 | 0.982201 | 5.92E-09 | 0.017468 | 0.824229 |
|  | rs13252808 | 8 | 71616655 | 0.023065 | 0.011305 | 0.75142 | 1.06E-07 | 0.001065 | 0.984173 |
|  | rs13255849 | 8 | 71752753 | 0.019195 | 0.013267 | 0.365122 | 9.79E-08 | 0.001147 | 0.976482 |
|  | rs13262350 | 8 | 71981606 | 0.017751 | 0.014207 | 0.269524 | 1.33E-09 | 0.074821 | 0.235164 |
|  | rs13268251 | 8 | 71978814 | 0.031434 | 0.008654 | 0.957984 | 3.58E-09 | 0.028474 | 0.674684 |
|  | rs13269690 | 8 | 71644807 | 0.021497 | 0.012019 | 0.564911 | 1.13E-07 | 0.000997 | 0.98822 |
|  | rs13271606 | 8 | 71741723 | 0.020546 | 0.012502 | 0.454829 | 8.76E-08 | 0.001278 | 0.964429 |
|  | rs13272884 | 8 | 71641648 | 0.027349 | 0.009755 | 0.94933 | 9.94E-08 | 0.001131 | 0.979883 |
|  | rs13273979 | 8 | 71737322 | 0.017042 | 0.014724 | 0.240825 | 9.44E-08 | 0.001189 | 0.971787 |
|  | rs13280922 | 8 | 71625398 | 0.023822 | 0.010993 | 0.795807 | 1.05E-07 | 0.001072 | 0.98204 |
|  | rs13281864 | 8 | 71642573 | 0.022083 | 0.011741 | 0.635948 | 6.66E-08 | 0.001666 | 0.960339 |
|  | rs1481796 | 8 | 71994350 | 0.033212 | 0.008255 | 0.966239 | 4.22E-09 | 0.024264 | 0.806762 |
|  | rs1481801 | 8 | 71989719 | 0.022988 | 0.011338 | 0.740114 | 1.33E-09 | 0.074986 | 0.160343 |
|  | rs1838392 | 8 | 71682583 | 0.022265 | 0.011657 | 0.682724 | 9.09E-08 | 0.001233 | 0.966916 |
|  | rs1973364 | 8 | 71867242 | 0.020804 | 0.012367 | 0.5044 | 1.32E-08 | 0.008023 | 0.875992 |
|  | rs2008517 | 8 | 71856520 | 0.024808 | 0.010613 | 0.817242 | 8.93E-09 | 0.011707 | 0.851177 |
|  | rs2380689 | 8 | 71681983 | 0.022125 | 0.011722 | 0.659398 | 9.15E-08 | 0.001225 | 0.970598 |
|  | rs2639917 | 8 | 71876891 | 0.020302 | 0.012633 | 0.442327 | 1.29E-08 | 0.008186 | 0.867969 |
|  | rs2639925 | 8 | 71904172 | 0.022299 | 0.011642 | 0.694366 | 1.79E-08 | 0.005975 | 0.953789 |
|  | rs2639929 | 8 | 71884648 | 0.021987 | 0.011785 | 0.61246 | 1.47E-08 | 0.007225 | 0.91266 |
|  | rs2639942 | 8 | 71913174 | 0.018127 | 0.013948 | 0.297659 | 1.42E-08 | 0.007477 | 0.883469 |
|  | rs2732098 | 8 | 71920997 | 0.017353 | 0.014492 | 0.255317 | 3.66E-09 | 0.027868 | 0.702552 |
|  | rs2732117 | 8 | 71909078 | 0.018534 | 0.01368 | 0.311339 | 1.55E-08 | 0.006841 | 0.947813 |
|  | rs2732119 | 8 | 71908377 | 0.021477 | 0.012029 | 0.552892 | 1.52E-08 | 0.006998 | 0.934028 |
|  | rs2732120 | 8 | 71904993 | 0.019959 | 0.012822 | 0.416967 | 1.53E-08 | 0.006945 | 0.940973 |
|  | rs2732124 | 8 | 71898682 | 0.024254 | 0.010822 | 0.806629 | 1.23E-08 | 0.008607 | 0.859784 |
|  | rs2732127 | 8 | 71896819 | 0.022069 | 0.011747 | 0.624207 | 1.44E-08 | 0.007347 | 0.890816 |
|  | rs2732130 | 8 | 71893473 | 0.023369 | 0.011177 | 0.773801 | 1.45E-08 | 0.007312 | 0.898128 |
|  | rs2732132 | 8 | 71891689 | 0.022538 | 0.011534 | 0.7059 | 1.47E-08 | 0.00722 | 0.91988 |
|  | rs2732133 | 8 | 71890976 | 0.021681 | 0.01193 | 0.588776 | 1.45E-08 | 0.007307 | 0.905435 |
|  | rs2732143 | 8 | 71875242 | 0.020874 | 0.01233 | 0.516731 | 1.48E-08 | 0.007149 | 0.92703 |
|  | rs28376252 | 8 | 71659887 | 0.02013 | 0.012727 | 0.429694 | 9.46E-08 | 0.001186 | 0.972973 |
|  | rs3098869 | 8 | 71929832 | 0.015 | 0.016472 | 0.100758 | 2.38E-09 | 0.042443 | 0.414168 |
|  | rs3098872 | 8 | 71932204 | 0.014953 | 0.016518 | 0.084286 | 2.14E-09 | 0.04697 | 0.282134 |
|  | rs3098875 | 8 | 71934688 | 0.015768 | 0.015764 | 0.165301 | 3.16E-09 | 0.032208 | 0.64621 |
|  | rs3098878 | 8 | 71937469 | 0.01515 | 0.016329 | 0.117087 | 2.23E-09 | 0.045125 | 0.327259 |
|  | rs3098882 | 8 | 71940094 | 0.007015 | 0.032395 | 0.032395 | 2.27E-09 | 0.044466 | 0.371725 |
|  | rs3110260 | 8 | 71940488 | 0.015231 | 0.016252 | 0.133339 | 2.47E-09 | 0.040902 | 0.496135 |
|  | rs3110261 | 8 | 71941451 | 0.015288 | 0.016198 | 0.149537 | 2.46E-09 | 0.041064 | 0.455232 |
|  | rs3110262 | 8 | 71941630 | 0.014632 | 0.016837 | 0.067768 | 2.48E-09 | 0.040806 | 0.536941 |
|  | rs3110264 | 8 | 71944280 | 0.013122 | 0.018536 | 0.050931 | 2.54E-09 | 0.039865 | 0.576806 |
|  | rs4074908 | 8 | 71955332 | 0.016625 | 0.015048 | 0.211077 | 3.67E-09 | 0.027853 | 0.730406 |
|  | rs4446768 | 8 | 71777589 | 0.020679 | 0.012432 | 0.467261 | 1.12E-07 | 0.001008 | 0.986216 |
|  | rs4623462 | 8 | 71946384 | 0.016188 | 0.015403 | 0.180705 | 2.72E-09 | 0.037197 | 0.614002 |
|  | rs62508859 | 8 | 71740934 | 0.016284 | 0.015324 | 0.196029 | 7.36E-08 | 0.001512 | 0.961851 |
|  | rs6472548 | 8 | 71754299 | 0.020753 | 0.012393 | 0.479655 | 1.12E-07 | 0.001007 | 0.987223 |
|  | rs6983845 | 8 | 71746480 | 0.019531 | 0.013067 | 0.391262 | 6.54E-08 | 0.001696 | 0.958673 |
|  | rs6984056 | 8 | 71771121 | 0.019851 | 0.012883 | 0.404145 | 9.93E-08 | 0.001132 | 0.978752 |
|  | rs6991708 | 8 | 71668528 | 0.02167 | 0.011935 | 0.576846 | 1.04E-07 | 0.001085 | 0.980968 |
|  | rs7009464 | 8 | 71781242 | 0.026338 | 0.010077 | 0.899906 | 1.14E-07 | 0.000993 | 0.989213 |
|  | rs7012796 | 8 | 71676193 | 0.020781 | 0.012379 | 0.492033 | 9.86E-08 | 0.001139 | 0.977621 |
|  | rs7013495 | 8 | 71693517 | 0.022239 | 0.011669 | 0.671067 | 9.54E-08 | 0.001177 | 0.975335 |
|  | rs7016334 | 8 | 71654406 | 0.021747 | 0.011899 | 0.600675 | 9.14E-08 | 0.001226 | 0.969374 |
|  | rs7812909 | 8 | 71997501 | 0.033218 | 0.008254 | 0.974493 | 3.97E-09 | 0.025762 | 0.782497 |
|  | rs7837005 | 8 | 71999032 | 0.04518 | 0.006356 | 0.988557 | 6.81E-09 | 0.015241 | 0.83947 |
|  | rs7838421 | 8 | 71672323 | 0.021425 | 0.012054 | 0.540863 | 1.06E-07 | 0.001067 | 0.983108 |
|  | rs7844508 | 8 | 71602995 | 0.017779 | 0.014187 | 0.283711 | 3.41E-08 | 0.003188 | 0.956977 |
|  | rs7845516 | 8 | 71685822 | 0.02211 | 0.011728 | 0.647676 | 8.6E-08 | 0.0013 | 0.963151 |
| rs1004173 | rs1004173 | 6 | 47445017 | 5.64E-09 | 0.023509 | 0.075601 | 0.005916 | 0.014884 | 0.339171 |
|  | rs10456570 | 6 | 47559451 | 1.04E-08 | 0.012936 | 0.633771 | 0.007562 | 0.011936 | 0.805503 |
|  | rs10948363 | 6 | 47487762 | 8.9E-09 | 0.015075 | 0.261658 | 0.005747 | 0.015278 | 0.279392 |
|  | rs10948367 | 6 | 47585615 | 1.56E-08 | 0.008718 | 0.94771 | 0.00807 | 0.011261 | 0.934074 |
|  | rs12193051 | 6 | 47541415 | 1.07E-08 | 0.01264 | 0.710359 | 0.007592 | 0.011894 | 0.82933 |
|  | rs12195738 | 6 | 47583664 | 1.78E-08 | 0.007701 | 0.971487 | 0.006145 | 0.014383 | 0.484746 |
|  | rs13193054 | 6 | 47552817 | 9.37E-09 | 0.014347 | 0.378431 | 0.007437 | 0.012116 | 0.733435 |
|  | rs13201473 | 6 | 47489708 | 1.16E-08 | 0.011606 | 0.82023 | 0.005936 | 0.014838 | 0.354009 |
|  | rs13212790 | 6 | 47576367 | 9.84E-09 | 0.013673 | 0.447627 | 0.007115 | 0.012607 | 0.658597 |
|  | rs1485780 | 6 | 47556630 | 8.56E-09 | 0.015656 | 0.231136 | 0.007647 | 0.011817 | 0.841148 |
|  | rs1872505 | 6 | 47480975 | 1.01E-08 | 0.01333 | 0.542008 | 0.005483 | 0.01594 | 0.171497 |
|  | rs1931837 | 6 | 47442377 | 5.42E-09 | 0.024433 | 0.052092 | 0.007479 | 0.012055 | 0.74549 |
|  | rs1948047 | 6 | 47585912 | 1.28E-08 | 0.010614 | 0.863779 | 0.009238 | 0.009979 | 0.976622 |
|  | rs2151974 | 6 | 47515630 | 1.06E-08 | 0.012763 | 0.685076 | 0.006659 | 0.01338 | 0.595018 |
|  | rs2151975 | 6 | 47515663 | 9.18E-09 | 0.014629 | 0.320564 | 0.006576 | 0.013532 | 0.568141 |
|  | rs2171086 | 6 | 47590104 | 1.49E-08 | 0.009163 | 0.921124 | 0.008411 | 0.010851 | 0.956139 |
|  | rs2171089 | 6 | 47515811 | 8.26E-09 | 0.016208 | 0.183792 | 0.006413 | 0.013841 | 0.554609 |
|  | rs2396825 | 6 | 47568696 | 9.91E-09 | 0.013582 | 0.461209 | 0.007496 | 0.012031 | 0.769573 |
|  | rs34744382 | 6 | 47536444 | 1.03E-08 | 0.013029 | 0.620835 | 0.00801 | 0.011336 | 0.922813 |
|  | rs4711878 | 6 | 47456118 | 1.13E-08 | 0.011984 | 0.796821 | 0.005738 | 0.015299 | 0.248818 |
|  | rs4711880 | 6 | 47480676 | 8.11E-09 | 0.016513 | 0.167583 | 0.006033 | 0.014623 | 0.398077 |
|  | rs4715018 | 6 | 47441871 | 5.91E-09 | 0.022444 | 0.098045 | 0.007181 | 0.012503 | 0.708816 |
|  | rs4715019 | 6 | 47447041 | 9.19E-09 | 0.014608 | 0.335172 | 0.006595 | 0.013497 | 0.581638 |
|  | rs4715025 | 6 | 47483653 | 8.37E-09 | 0.016007 | 0.199799 | 0.005975 | 0.014751 | 0.36876 |
|  | rs6903331 | 6 | 47562915 | 9.31E-09 | 0.014431 | 0.364084 | 0.00751 | 0.01201 | 0.781583 |
|  | rs6904764 | 6 | 47503497 | 9.96E-09 | 0.013521 | 0.488309 | 0.006118 | 0.01444 | 0.455978 |
|  | rs6913202 | 6 | 47489104 | 9.06E-09 | 0.014823 | 0.27648 | 0.005911 | 0.014895 | 0.324287 |
|  | rs6931011 | 6 | 47525202 | 1.66E-08 | 0.008227 | 0.955938 | 0.005055 | 0.017154 | 0.072418 |
|  | rs6931478 | 6 | 47461913 | 1.02E-08 | 0.01316 | 0.581603 | 0.004669 | 0.018432 | 0.037429 |
|  | rs7738044 | 6 | 47469273 | 1.05E-08 | 0.012785 | 0.672312 | 0.005441 | 0.016051 | 0.155557 |
|  | rs7749167 | 6 | 47493940 | 1.4E-08 | 0.009723 | 0.883723 | 0.005739 | 0.015297 | 0.264114 |
|  | rs7749271 | 6 | 47485002 | 1.09E-08 | 0.012363 | 0.76041 | 0.005116 | 0.016969 | 0.106404 |
|  | rs7754282 | 6 | 47502024 | 9.42E-09 | 0.014262 | 0.392693 | 0.006089 | 0.014502 | 0.412579 |
|  | rs7754971 | 6 | 47590476 | 1.23E-08 | 0.011009 | 0.831239 | 0.008108 | 0.011214 | 0.945288 |
|  | rs7767350 | 6 | 47485126 | 7.78E-09 | 0.01718 | 0.151071 | 0.006173 | 0.014324 | 0.49907 |
|  | rs7774667 | 6 | 47514758 | 9.28E-09 | 0.014481 | 0.349653 | 0.00639 | 0.013886 | 0.526916 |
|  | rs901186 | 6 | 47514246 | 2.01E-08 | 0.006846 | 0.993397 | 0.006407 | 0.013852 | 0.540768 |
|  | rs9296558 | 6 | 47451883 | 9.7E-09 | 0.013864 | 0.406558 | 0.007139 | 0.012569 | 0.683747 |
|  | rs9296559 | 6 | 47452270 | 1.02E-08 | 0.01325 | 0.555258 | 0.007181 | 0.012503 | 0.721319 |
|  | rs9296561 | 6 | 47488938 | 1.08E-08 | 0.012537 | 0.73551 | 0.005264 | 0.016537 | 0.139507 |
|  | rs9296564 | 6 | 47494759 | 8.68E-09 | 0.015446 | 0.246583 | 0.006105 | 0.014468 | 0.441538 |
|  | rs9296567 | 6 | 47553402 | 1.51E-08 | 0.009007 | 0.930131 | 0.005737 | 0.015302 | 0.233518 |
|  | rs9349409 | 6 | 47463126 | 1.11E-08 | 0.012138 | 0.784837 | 0.0051 | 0.017017 | 0.089435 |
|  | rs9349413 | 6 | 47511491 | 1.74E-08 | 0.007848 | 0.963786 | 0.004516 | 0.018997 | 0.018997 |
|  | rs9349415 | 6 | 47551861 | 1.05E-08 | 0.012879 | 0.646651 | 0.007648 | 0.011816 | 0.876597 |
|  | rs9349416 | 6 | 47551938 | 1.05E-08 | 0.012877 | 0.659528 | 0.007648 | 0.011816 | 0.864781 |
|  | rs9349417 | 6 | 47580657 | 1.14E-08 | 0.011803 | 0.808624 | 0.007675 | 0.011779 | 0.888375 |
|  | rs9357546 | 6 | 47549495 | 1E-08 | 0.013428 | 0.528678 | 0.007141 | 0.012566 | 0.696313 |
|  | rs9367279 | 6 | 47448336 | 1.02E-08 | 0.013158 | 0.594761 | 0.006885 | 0.012985 | 0.608002 |
|  | rs9367284 | 6 | 47516369 | 9.12E-09 | 0.014728 | 0.291209 | 0.005888 | 0.014947 | 0.309393 |
|  | rs9369693 | 6 | 47433751 | 1.03E-08 | 0.013046 | 0.607807 | 0.01716 | 0.005768 | 0.98239 |
|  | rs9369695 | 6 | 47440565 | 4.77E-09 | 0.027659 | 0.027659 | 0.008723 | 0.010504 | 0.966643 |
|  | rs9369716 | 6 | 47552180 | 8.55E-09 | 0.015682 | 0.21548 | 0.007481 | 0.012052 | 0.757542 |
|  | rs9369717 | 6 | 47554468 | 1.79E-08 | 0.007642 | 0.97913 | 0.00551 | 0.015869 | 0.187366 |
|  | rs9381562 | 6 | 47429767 | 7.56E-09 | 0.017667 | 0.133891 | 0.02456 | 0.00422 | 0.991749 |
|  | rs9381564 | 6 | 47443806 | 7.34E-09 | 0.018178 | 0.116223 | 0.006352 | 0.01396 | 0.513031 |
|  | rs9381575 | 6 | 47528764 | 9.96E-09 | 0.013511 | 0.50182 | 0.006094 | 0.014491 | 0.42707 |
|  | rs9381578 | 6 | 47556634 | 9.82E-09 | 0.013709 | 0.420267 | 0.007647 | 0.011817 | 0.852965 |
|  | rs9381579 | 6 | 47556680 | 1.46E-08 | 0.009339 | 0.902631 | 0.007564 | 0.011934 | 0.817436 |
|  | rs9381581 | 6 | 47580695 | 9.91E-09 | 0.013579 | 0.474788 | 0.00777 | 0.01165 | 0.900025 |
|  | rs9395262 | 6 | 47465267 | 1.1E-08 | 0.012289 | 0.772699 | 0.005254 | 0.016566 | 0.122969 |
|  | rs9395279 | 6 | 47542864 | 1.08E-08 | 0.012537 | 0.748047 | 0.007131 | 0.012582 | 0.671178 |
|  | rs9395283 | 6 | 47551444 | 1.07E-08 | 0.012643 | 0.697719 | 0.007529 | 0.011983 | 0.793566 |
|  | rs9395285 | 6 | 47554177 | 1.85E-08 | 0.007422 | 0.986551 | 0.005679 | 0.015442 | 0.202809 |
|  | rs9395286 | 6 | 47575332 | 1.23E-08 | 0.010988 | 0.842227 | 0.007087 | 0.012652 | 0.633351 |
|  | rs9463335 | 6 | 47479136 | 1.46E-08 | 0.00933 | 0.911961 | 0.004842 | 0.017835 | 0.055264 |
|  | rs9463342 | 6 | 47585106 | 1.42E-08 | 0.009568 | 0.893291 | 0.00792 | 0.011452 | 0.911477 |
|  | rs9473117 | 6 | 47431284 | 1.24E-08 | 0.010938 | 0.853165 | 0.01958 | 0.005139 | 0.98753 |
|  | rs9473119 | 6 | 47450618 | 1.02E-08 | 0.013184 | 0.568442 | 0.007095 | 0.012639 | 0.64599 |
|  | rs9473122 | 6 | 47474962 | 1E-08 | 0.013429 | 0.515249 | 0.005693 | 0.015408 | 0.218217 |
|  | rs9473123 | 6 | 47475339 | 1.07E-08 | 0.012613 | 0.722972 | 0.005842 | 0.015053 | 0.294445 |
|  | rs9473126 | 6 | 47481833 | 9.12E-09 | 0.014727 | 0.305935 | 0.006001 | 0.014693 | 0.383454 |
|  | rs9473128 | 6 | 47505010 | 9.83E-09 | 0.013687 | 0.433954 | 0.006144 | 0.014385 | 0.470363 |
| rs11100203 | rs11100203 | 4 | 1.6E+08 | 4.13E-06 | 0.748692 | 0.748692 | 2.93E-05 | 0.477483 | 1 |
|  | rs2114489 | 4 | 1.6E+08 | 1.29E-05 | 0.251308 | 1 | 2.67E-05 | 0.522517 | 0.522517 |
| rs11642303 | rs1108431 | 16 | 31054607 | 6.27E-05 | 0.009635 | 0.962579 | 1.35E-09 | 0.043642 | 0.087411 |
|  | rs112906665 | 16 | 31040139 | 3.38E-05 | 0.01733 | 0.67285 | 2.48E-09 | 0.024157 | 0.702049 |
|  | rs11640767 | 16 | 31060647 | 3.99E-05 | 0.014804 | 0.782119 | 1.63E-09 | 0.036454 | 0.247268 |
|  | rs11640957 | 16 | 31060363 | 4.44E-05 | 0.01337 | 0.880145 | 1.83E-09 | 0.032486 | 0.313668 |
|  | rs11642003 | 16 | 31068391 | 7.27E-06 | 0.074967 | 0.074967 | 2.2E-09 | 0.027185 | 0.624242 |
|  | rs11642192 | 16 | 31082025 | 1.94E-05 | 0.029333 | 0.428405 | 6.15E-09 | 0.009981 | 0.916473 |
|  | rs11642303 | 16 | 31068392 | 7.71E-06 | 0.070854 | 0.145821 | 2.04E-09 | 0.029152 | 0.402291 |
|  | rs11647284 | 16 | 31077335 | 2.85E-05 | 0.020361 | 0.636098 | 7.25E-09 | 0.008501 | 0.979559 |
|  | rs11862744 | 16 | 31057800 | 3.7E-05 | 0.015899 | 0.721962 | 2.16E-09 | 0.027588 | 0.569581 |
|  | rs11864839 | 16 | 31095251 | 1.46E-05 | 0.038617 | 0.307068 | 7.01E-09 | 0.008784 | 0.953817 |
|  | rs11865038 | 16 | 31095171 | 1.31E-05 | 0.042642 | 0.188464 | 6.25E-09 | 0.009815 | 0.926287 |
|  | rs12445650 | 16 | 31044897 | 5.18E-05 | 0.011552 | 0.942762 | 2.16E-09 | 0.027601 | 0.514404 |
|  | rs12447930 | 16 | 31023273 | 4.2E-05 | 0.01408 | 0.825462 | 2.71E-09 | 0.022118 | 0.769458 |
|  | rs17839567 | 16 | 31057945 | 4.02E-05 | 0.014707 | 0.796826 | 2.16E-09 | 0.027588 | 0.541992 |
|  | rs17839568 | 16 | 31099783 | 1.94E-05 | 0.029298 | 0.457704 | 5.98E-09 | 0.010249 | 0.886482 |
|  | rs28814987 | 16 | 31081460 | 3E-05 | 0.019422 | 0.65552 | 3.79E-09 | 0.015978 | 0.827658 |
|  | rs35468353 | 16 | 31056433 | 4.21E-05 | 0.01407 | 0.839532 | 1.99E-09 | 0.029996 | 0.343664 |
|  | rs35961830 | 16 | 31032317 | 3.59E-05 | 0.016369 | 0.706064 | 2.25E-09 | 0.026549 | 0.677892 |
|  | rs3751855 | 16 | 31091209 | 1.35E-05 | 0.041351 | 0.229814 | 7.24E-09 | 0.008512 | 0.971058 |
|  | rs4468641 | 16 | 31096876 | 1.88E-05 | 0.030272 | 0.399072 | 5.92E-09 | 0.01036 | 0.876234 |
|  | rs4889526 | 16 | 31030344 | 2.23E-05 | 0.025676 | 0.567289 | 2.84E-09 | 0.021132 | 0.79059 |
|  | rs4889609 | 16 | 31026427 | 3.9E-05 | 0.015104 | 0.752285 | 2.85E-09 | 0.021089 | 0.811679 |
|  | rs55979739 | 16 | 31049155 | 6.55E-05 | 0.009248 | 0.971828 | 1.49E-09 | 0.039591 | 0.210814 |
|  | rs56284083 | 16 | 31052895 | 4.29E-05 | 0.013824 | 0.853356 | 2.14E-09 | 0.027839 | 0.486803 |
|  | rs57434408 | 16 | 31066380 | 4.06E-05 | 0.014556 | 0.811382 | 1.75E-09 | 0.033914 | 0.281182 |
|  | rs57576577 | 16 | 31036367 | 3.92E-05 | 0.01503 | 0.767315 | 2.12E-09 | 0.028172 | 0.458964 |
|  | rs58726213 | 16 | 31044683 | 4.42E-05 | 0.013419 | 0.866775 | 2.09E-09 | 0.0285 | 0.430791 |
|  | rs59061704 | 16 | 31062704 | 4.65E-05 | 0.012808 | 0.919362 | 1.46E-09 | 0.040545 | 0.171223 |
|  | rs6565217 | 16 | 31083324 | 2.19E-05 | 0.026141 | 0.541613 | 6.41E-09 | 0.009587 | 0.935874 |
|  | rs7184567 | 16 | 31021078 | 4.55E-05 | 0.013054 | 0.906555 | 2.61E-09 | 0.022941 | 0.72499 |
|  | rs7196726 | 16 | 31092075 | 1.84E-05 | 0.030811 | 0.3688 | 4.8E-09 | 0.012691 | 0.853343 |
|  | rs7197717 | 16 | 31083075 | 1.95E-05 | 0.029195 | 0.486899 | 6.14E-09 | 0.00999 | 0.906492 |
|  | rs7199949 | 16 | 31096164 | 1.45E-05 | 0.038637 | 0.268451 | 6.72E-09 | 0.009159 | 0.945033 |
|  | rs72800847 | 16 | 31022639 | 5.04E-05 | 0.011848 | 0.93121 | 4.87E-09 | 0.012531 | 0.865874 |
|  | rs7294 | 16 | 31102321 | 1.84E-05 | 0.030921 | 0.337989 | 6.12E-09 | 0.010019 | 0.896501 |
|  | rs729482 | 16 | 31016970 | 5.92E-05 | 0.010183 | 0.952945 | 7.98E-09 | 0.007746 | 0.987305 |
|  | rs732172 | 16 | 31050033 | 7.75E-05 | 0.007885 | 0.996475 | 1.35E-09 | 0.043768 | 0.043768 |
|  | rs732173 | 16 | 31050023 | 7.23E-05 | 0.008422 | 0.98025 | 1.36E-09 | 0.043268 | 0.130678 |
|  | rs73530203 | 16 | 31099859 | 2.46E-05 | 0.023394 | 0.615737 | 9.04E-09 | 0.006862 | 0.994167 |
|  | rs7500176 | 16 | 31041137 | 7.31E-05 | 0.008341 | 0.98859 | 2.69E-09 | 0.02235 | 0.74734 |
|  | rs750952 | 16 | 31093954 | 2E-05 | 0.028573 | 0.515472 | 4.69E-09 | 0.012994 | 0.840652 |
|  | rs8056842 | 16 | 31047330 | 4.45E-05 | 0.013355 | 0.8935 | 2.02E-09 | 0.029475 | 0.373139 |
|  | rs8061047 | 16 | 31068960 | 3.48E-05 | 0.016845 | 0.689694 | 2.17E-09 | 0.027477 | 0.597058 |
|  | rs9673641 | 16 | 31039376 | 3.87E-05 | 0.015219 | 0.737181 | 2.2E-09 | 0.0271 | 0.651343 |
| rs147188206 | rs147188206 | 19 | 45552587 | 2.62E-14 | 1 | 1 | 0.002893 | 1 | 1 |
| rs204911 | rs190651665 | 19 | 45456103 | 1.06E-09 | 0.195063 | 0.806654 | 0.007915 | 0.224911 | 1 |
|  | rs204906 | 19 | 45461980 | 1.07E-09 | 0.193346 | 1 | 0.007007 | 0.250883 | 0.775089 |
|  | rs204910 | 19 | 45463385 | 6.75E-10 | 0.302066 | 0.611591 | 0.006685 | 0.261715 | 0.524207 |
|  | rs204911 | 19 | 45463540 | 6.58E-10 | 0.309525 | 0.309525 | 0.006663 | 0.262492 | 0.262492 |
| rs28469095 | rs10401157 | 19 | 45642044 | 5.21E-38 | 0.077103 | 0.571857 | 0.007344 | 0.053649 | 0.620813 |
|  | rs10401823 | 19 | 45642545 | 8.22E-38 | 0.049053 | 0.868386 | 0.007997 | 0.049705 | 0.77333 |
|  | rs1048699 | 19 | 45650386 | 2.16E-37 | 0.018778 | 0.973967 | 0.008446 | 0.047334 | 0.869889 |
|  | rs1114831 | 19 | 45636319 | 4.7E-37 | 0.008667 | 0.99699 | 0.006779 | 0.057647 | 0.455622 |
|  | rs113321260 | 19 | 45651237 | 3.25E-38 | 0.123335 | 0.494755 | 0.008463 | 0.047249 | 0.917137 |
|  | rs12462040 | 19 | 45612226 | 1.64E-37 | 0.024689 | 0.893075 | 0.00788 | 0.050365 | 0.723625 |
|  | rs17643262 | 19 | 45631816 | 1.89E-37 | 0.02139 | 0.914465 | 0.007109 | 0.055237 | 0.567165 |
|  | rs2004357 | 19 | 45618959 | 7.1E-38 | 0.056737 | 0.76852 | 0.006321 | 0.06139 | 0.276982 |
|  | rs28469095 | 19 | 45655333 | 1.07E-38 | 0.37142 | 0.37142 | 0.003141 | 0.115686 | 0.115686 |
|  | rs28620490 | 19 | 45638245 | 7.93E-38 | 0.050813 | 0.819333 | 0.006455 | 0.060242 | 0.397975 |
|  | rs34545713 | 19 | 45614601 | 1.96E-37 | 0.020692 | 0.935158 | 0.006959 | 0.056305 | 0.511927 |
|  | rs7248421 | 19 | 45606125 | 2.83E-37 | 0.014356 | 0.988323 | 0.009508 | 0.042583 | 0.95972 |
|  | rs74846209 | 19 | 45650099 | 5.62E-38 | 0.071546 | 0.643404 | 0.01012 | 0.04028 | 1 |
|  | rs754366 | 19 | 45633686 | 2.02E-37 | 0.020031 | 0.955189 | 0.008084 | 0.049226 | 0.822555 |
|  | rs78273125 | 19 | 45644354 | 5.88E-38 | 0.068379 | 0.711783 | 0.007532 | 0.052446 | 0.673259 |
| rs3098882 | rs10090230 | 8 | 71797013 | 0.027109 | 0.010405 | 0.9563 | 1.18E-07 | 0.001197 | 0.987823 |
|  | rs10090471 | 8 | 71797269 | 0.025581 | 0.01094 | 0.881877 | 1.18E-07 | 0.001197 | 0.98902 |
|  | rs10429277 | 8 | 71701391 | 0.019522 | 0.013838 | 0.386105 | 9.1E-08 | 0.001543 | 0.960246 |
|  | rs10453108 | 8 | 71782940 | 0.025573 | 0.010943 | 0.870937 | 1.09E-07 | 0.001296 | 0.98161 |
|  | rs11779364 | 8 | 71954596 | 0.016654 | 0.015905 | 0.239342 | 3.88E-09 | 0.032972 | 0.746847 |
|  | rs11989644 | 8 | 71641196 | 0.023771 | 0.011658 | 0.792477 | 9.48E-08 | 0.001483 | 0.967772 |
|  | rs11994908 | 8 | 71721619 | 0.018612 | 0.014428 | 0.343999 | 8.93E-08 | 0.00157 | 0.95716 |
|  | rs12155783 | 8 | 71999126 | 0.035992 | 0.008159 | 0.992685 | 5.92E-09 | 0.021874 | 0.799107 |
|  | rs13252808 | 8 | 71616655 | 0.023065 | 0.011967 | 0.757127 | 1.06E-07 | 0.001334 | 0.980314 |
|  | rs13255849 | 8 | 71752753 | 0.019195 | 0.014044 | 0.372267 | 9.79E-08 | 0.001437 | 0.970683 |
|  | rs13262350 | 8 | 71981606 | 0.017751 | 0.015039 | 0.285307 | 1.33E-09 | 0.093696 | 0.093696 |
|  | rs13268251 | 8 | 71978814 | 0.031434 | 0.009161 | 0.975787 | 3.58E-09 | 0.035657 | 0.644096 |
|  | rs13269690 | 8 | 71644807 | 0.021497 | 0.012723 | 0.583755 | 1.13E-07 | 0.001248 | 0.985383 |
|  | rs13271606 | 8 | 71741723 | 0.020546 | 0.013234 | 0.467227 | 8.76E-08 | 0.0016 | 0.955589 |
|  | rs13272884 | 8 | 71641648 | 0.027349 | 0.010326 | 0.966627 | 9.94E-08 | 0.001416 | 0.974942 |
|  | rs13273979 | 8 | 71737322 | 0.017042 | 0.015586 | 0.254928 | 9.44E-08 | 0.001489 | 0.964804 |
|  | rs13280922 | 8 | 71625398 | 0.023822 | 0.011636 | 0.804113 | 1.05E-07 | 0.001343 | 0.977643 |
|  | rs13281864 | 8 | 71642573 | 0.022083 | 0.012428 | 0.658952 | 6.66E-08 | 0.002086 | 0.950467 |
|  | rs1481796 | 8 | 71994350 | 0.033212 | 0.008739 | 0.984526 | 4.22E-09 | 0.030386 | 0.777233 |
|  | rs1838392 | 8 | 71682583 | 0.022265 | 0.01234 | 0.708467 | 9.09E-08 | 0.001544 | 0.958703 |
|  | rs1973364 | 8 | 71867242 | 0.020804 | 0.013091 | 0.519701 | 1.32E-08 | 0.010046 | 0.844842 |
|  | rs2008517 | 8 | 71856520 | 0.024808 | 0.011234 | 0.826803 | 8.93E-09 | 0.01466 | 0.813767 |
|  | rs2380689 | 8 | 71681983 | 0.022125 | 0.012408 | 0.683775 | 9.15E-08 | 0.001534 | 0.963315 |
|  | rs2639917 | 8 | 71876891 | 0.020302 | 0.013373 | 0.453993 | 1.29E-08 | 0.010251 | 0.834795 |
|  | rs2639925 | 8 | 71904172 | 0.022299 | 0.012323 | 0.720791 | 1.79E-08 | 0.007483 | 0.942265 |
|  | rs2639929 | 8 | 71884648 | 0.021987 | 0.012476 | 0.634089 | 1.47E-08 | 0.009048 | 0.890761 |
|  | rs2639942 | 8 | 71913174 | 0.018127 | 0.014765 | 0.31509 | 1.42E-08 | 0.009364 | 0.854206 |
|  | rs2732098 | 8 | 71920997 | 0.017353 | 0.015341 | 0.270268 | 3.66E-09 | 0.034899 | 0.678994 |
|  | rs2732117 | 8 | 71909078 | 0.018534 | 0.014481 | 0.329571 | 1.55E-08 | 0.008566 | 0.934782 |
|  | rs2732119 | 8 | 71908377 | 0.021477 | 0.012733 | 0.571033 | 1.52E-08 | 0.008764 | 0.917519 |
|  | rs2732120 | 8 | 71904993 | 0.019959 | 0.013573 | 0.427148 | 1.53E-08 | 0.008697 | 0.926216 |
|  | rs2732124 | 8 | 71898682 | 0.024254 | 0.011456 | 0.815569 | 1.23E-08 | 0.010778 | 0.824545 |
|  | rs2732127 | 8 | 71896819 | 0.022069 | 0.012435 | 0.646524 | 1.44E-08 | 0.0092 | 0.863406 |
|  | rs2732130 | 8 | 71893473 | 0.023369 | 0.011832 | 0.780819 | 1.45E-08 | 0.009157 | 0.872562 |
|  | rs2732132 | 8 | 71891689 | 0.022538 | 0.01221 | 0.733001 | 1.47E-08 | 0.009042 | 0.899802 |
|  | rs2732133 | 8 | 71890976 | 0.021681 | 0.012629 | 0.609018 | 1.45E-08 | 0.009151 | 0.881713 |
|  | rs2732143 | 8 | 71875242 | 0.020874 | 0.013052 | 0.532754 | 1.48E-08 | 0.008953 | 0.908755 |
|  | rs28376252 | 8 | 71659887 | 0.02013 | 0.013472 | 0.440621 | 9.46E-08 | 0.001486 | 0.966289 |
|  | rs3098869 | 8 | 71929832 | 0.015 | 0.017437 | 0.106659 | 2.38E-09 | 0.05315 | 0.317859 |
|  | rs3098872 | 8 | 71932204 | 0.014953 | 0.017485 | 0.089222 | 2.14E-09 | 0.05882 | 0.152516 |
|  | rs3098875 | 8 | 71934688 | 0.015768 | 0.016687 | 0.174981 | 3.16E-09 | 0.040333 | 0.608439 |
|  | rs3098878 | 8 | 71937469 | 0.01515 | 0.017285 | 0.123944 | 2.23E-09 | 0.056509 | 0.209025 |
|  | rs3098882 | 8 | 71940094 | 0.007015 | 0.034292 | 0.034292 | 2.27E-09 | 0.055683 | 0.264709 |
|  | rs3110260 | 8 | 71940488 | 0.015231 | 0.017204 | 0.141147 | 2.47E-09 | 0.051221 | 0.420503 |
|  | rs3110261 | 8 | 71941451 | 0.015288 | 0.017147 | 0.158294 | 2.46E-09 | 0.051424 | 0.369282 |
|  | rs3110262 | 8 | 71941630 | 0.014632 | 0.017823 | 0.071737 | 2.48E-09 | 0.0511 | 0.471603 |
|  | rs3110264 | 8 | 71944280 | 0.013122 | 0.019622 | 0.053914 | 2.54E-09 | 0.049922 | 0.521526 |
|  | rs4074908 | 8 | 71955332 | 0.016625 | 0.015929 | 0.223437 | 3.67E-09 | 0.03488 | 0.713875 |
|  | rs4446768 | 8 | 71777589 | 0.020679 | 0.01316 | 0.480387 | 1.12E-07 | 0.001262 | 0.982873 |
|  | rs4623462 | 8 | 71946384 | 0.016188 | 0.016305 | 0.191287 | 2.72E-09 | 0.04658 | 0.568106 |
|  | rs62508859 | 8 | 71740934 | 0.016284 | 0.016222 | 0.207508 | 7.36E-08 | 0.001893 | 0.95236 |
|  | rs6472548 | 8 | 71754299 | 0.020753 | 0.013119 | 0.493507 | 1.12E-07 | 0.001261 | 0.984134 |
|  | rs6983845 | 8 | 71746480 | 0.019531 | 0.013832 | 0.399938 | 6.54E-08 | 0.002124 | 0.948381 |
|  | rs6984056 | 8 | 71771121 | 0.019851 | 0.013637 | 0.413575 | 9.93E-08 | 0.001417 | 0.973526 |
|  | rs6984663 | 8 | 71827665 | 0.026761 | 0.010522 | 0.935477 | 1.22E-07 | 0.001161 | 0.990182 |
|  | rs6991708 | 8 | 71668528 | 0.02167 | 0.012634 | 0.596389 | 1.04E-07 | 0.001359 | 0.976301 |
|  | rs7009464 | 8 | 71781242 | 0.026338 | 0.010667 | 0.914308 | 1.14E-07 | 0.001243 | 0.986626 |
|  | rs7012796 | 8 | 71676193 | 0.020781 | 0.013104 | 0.50661 | 9.86E-08 | 0.001426 | 0.972109 |
|  | rs7013495 | 8 | 71693517 | 0.022239 | 0.012352 | 0.696127 | 9.54E-08 | 0.001474 | 0.969246 |
|  | rs7016334 | 8 | 71654406 | 0.021747 | 0.012595 | 0.621614 | 9.14E-08 | 0.001536 | 0.961782 |
|  | rs7838421 | 8 | 71672323 | 0.021425 | 0.01276 | 0.5583 | 1.06E-07 | 0.001337 | 0.97898 |
|  | rs7844508 | 8 | 71602995 | 0.017779 | 0.015018 | 0.300325 | 3.41E-08 | 0.003992 | 0.946257 |
|  | rs7845516 | 8 | 71685822 | 0.02211 | 0.012415 | 0.671367 | 8.6E-08 | 0.001628 | 0.953989 |
| rs439401 | rs439401 | 19 | 45414451 | 7.7E-167 | 0.566159 | 0.566159 | 0.002209 | 0.404302 | 1 |
|  | rs584007 | 19 | 45416478 | 1E-166 | 0.433841 | 1 | 0.001449 | 0.595698 | 0.595698 |
| rs61597598 | rs113707721 | 2 | 1.57E+08 | 0.00624 | 0.103561 | 0.390544 | 5.32E-14 | 0.094526 | 0.581215 |
|  | rs140812695 | 2 | 1.57E+08 | 0.003396 | 0.179654 | 0.179654 | 5.76E-14 | 0.087515 | 0.758641 |
|  | rs61597598 | 2 | 1.57E+08 | 0.005997 | 0.107329 | 0.286983 | 4.52E-14 | 0.111043 | 0.38085 |
|  | rs72902175 | 2 | 1.57E+08 | 0.014011 | 0.050337 | 0.904303 | 5.6E-14 | 0.089911 | 0.671126 |
|  | rs72902177 | 2 | 1.57E+08 | 0.009935 | 0.068276 | 0.790087 | 4.74E-14 | 0.105839 | 0.486689 |
|  | rs72904207 | 2 | 1.57E+08 | 0.006877 | 0.094892 | 0.581759 | 7.38E-14 | 0.068544 | 0.990954 |
|  | rs72904209 | 2 | 1.57E+08 | 0.009527 | 0.070877 | 0.652636 | 5.98E-14 | 0.084235 | 0.842876 |
|  | rs72904292 | 2 | 1.57E+08 | 0.006763 | 0.096323 | 0.486867 | 6.34E-14 | 0.079534 | 0.92241 |
|  | rs72906130 | 2 | 1.57E+08 | 0.010707 | 0.063879 | 0.853966 | 3.5E-14 | 0.142733 | 0.142733 |
|  | rs72907878 | 2 | 1.57E+08 | 0.015156 | 0.046968 | 0.951271 | 3.94E-14 | 0.127074 | 0.269807 |
| rs62402786 | rs62402786 | 6 | 22307131 | 2.89E-07 | 0.329769 | 1 | 0.001672 | 0.533635 | 0.533635 |
|  | rs6910948 | 6 | 22306698 | 1.39E-07 | 0.670231 | 0.670231 | 0.001936 | 0.466365 | 1 |
| rs7514002 | rs2457816 | 1 | 62604065 | 0.001098 | 0.120419 | 0.691946 | 1.82E-07 | 0.224972 | 0.746356 |
|  | rs2457817 | 1 | 62604570 | 0.001189 | 0.111814 | 0.803761 | 1.71E-07 | 0.238164 | 0.521384 |
|  | rs2476198 | 1 | 62611071 | 0.000843 | 0.153718 | 0.571528 | 3.08E-06 | 0.014741 | 0.985713 |
|  | rs2476201 | 1 | 62619960 | 0.001839 | 0.074819 | 0.96457 | 2.65E-07 | 0.156286 | 0.902641 |
|  | rs2481665 | 1 | 62594677 | 0.001581 | 0.085991 | 0.889751 | 9.3E-07 | 0.046572 | 0.949214 |
|  | rs2481671 | 1 | 62611666 | 0.000486 | 0.25671 | 0.25671 | 2.05E-06 | 0.021758 | 0.970972 |
|  | rs2481677 | 1 | 62632571 | 0.00417 | 0.03543 | 1 | 3.18E-06 | 0.014287 | 1 |
|  | rs7514002 | 1 | 62616599 | 0.000802 | 0.161099 | 0.41781 | 1.43E-07 | 0.28322 | 0.28322 |

## Supplementary Table9 Co-localization analysis of both Alzheimer’s disease and sleep-related phenotypes are associated and share causal variants

| Model | snp | PP.H3 | PP.H4 |
| --- | --- | --- | --- |
| AD_Insomnia | rs11234556 | 0.064 | 0.011964591 |
|  | rs150567157 | 0.04 | 0.175016625 |
|  | rs186110295 | 0.043 | 0.174466354 |
|  | rs2249152 | 0.148 | 0.181376691 |
|  | rs606757 | 0.042 | 0.174607936 |
|  | rs6857 | 0.044 | 0.174276557 |
|  | rs9268428 | 0.124 | 0.032258355 |
| AD_Sleepdur | rs1081105 | 0.051 | 0.075185163 |
|  | rs11672748 | 0.064 | 0.074170058 |
|  | rs12292911 | 0.114 | 0.613597719 |
|  | rs12972970 | 0.052 | 0.075112345 |
|  | rs1633096 | 0.116 | 0.032740156 |
|  | rs1979377 | 0.057 | 0.074616496 |
|  | rs2310752 | 0.092 | 0.097211895 |
|  | rs3121427 | 0.03 | 0.417198652 |
|  | rs359539 | 0.361 | 0.152994987 |
|  | rs4727449 | 0.032 | 0.00615115 |
|  | rs56249331 | 0.034 | 0.095369616 |
|  | rs858502 | 0.033 | 0.006143721 |
| AD_Snoring | rs1004173 | 0.081 | 0.064629972 |
|  | rs11100203 | 0.097 | 0.203561172 |
|  | rs11642303 | 0.812 | 0.165757727 |
|  | rs147188206 | 0.073 | 0.016179154 |
|  | rs204911 | 0.074 | 0.016160763 |
|  | rs28469095 | 0.076 | 0.016123541 |
|  | rs3098882 | 0.021 | 0.002457858 |
|  | rs429358 | 0.073 | 0.016183175 |
|  | rs439401 | 0.073 | 0.016183803 |
|  | rs61597598 | 0.029 | 0.052576332 |
|  | rs62402786 | 0.09 | 0.176325713 |
|  | rs7514002 | 0.031 | 0.126975758 |

*Note*: PP.H3: Association with trait 1 and trait 2, two independent SNPs. PP.H4: Association with trait 1 and trait 2, one shared SNP.

## Supplementary Table10. Biological process of the shared gene set between AD and insomnia in KEGG pathways

| KEGG ID | KEGG Term | Term PValue | Term PValue Corrected with Bonferroni step down | % Associated Genes | Nr. Genes | Associated Genes Found |
| --- | --- | --- | --- | --- | --- | --- |
| KEGG:04672 | Intestinal immune network for IgA production | 7.36E-06 | 2.95E-05 | 6.12 | 3.00 | [HLA-DRA, HLA-DRB1, HLA-DRB5] |
| KEGG:04940 | Type I diabetes mellitus | 4.94E-06 | 2.47E-05 | 6.98 | 3.00 | [HLA-DRA, HLA-DRB1, HLA-DRB5] |
| KEGG:05310 | Asthma | 1.81E-06 | 1.45E-05 | 9.68 | 3.00 | [HLA-DRA, HLA-DRB1, HLA-DRB5] |
| KEGG:05320 | Autoimmune thyroid disease | 9.35E-06 | 2.80E-05 | 5.66 | 3.00 | [HLA-DRA, HLA-DRB1, HLA-DRB5] |
| KEGG:05321 | Inflammatory bowel disease (IBD) | 1.74E-05 | 1.74E-05 | 4.62 | 3.00 | [HLA-DRA, HLA-DRB1, HLA-DRB5] |
| KEGG:05330 | Allograft rejection | 3.39E-06 | 2.37E-05 | 7.89 | 3.00 | [HLA-DRA, HLA-DRB1, HLA-DRB5] |
| KEGG:05332 | Graft-versus-host disease | 4.27E-06 | 2.56E-05 | 7.32 | 3.00 | [HLA-DRA, HLA-DRB1, HLA-DRB5] |
| KEGG:05416 | Viral myocarditis | 1.36E-05 | 2.72E-05 | 5.00 | 3.00 | [HLA-DRA, HLA-DRB1, HLA-DRB5] |

## Supplementary Table11. Biological process of the shared gene set between AD and sleep duration in GO terms.

| GOID | GOTerm | Term PValue | Term PValue Corrected with Bonferroni step down | % Associated Genes | Nr. Genes | Associated Genes Found |
| --- | --- | --- | --- | --- | --- | --- |
| GO:0034447 | very-low-density lipoprotein particle clearance | 2.45E-11 | 4.66E-10 | 50.00 | 5.00 | [APOC1, APOC2, APOC4, APOC4-APOC2, APOE] |
| GO:0071830 | triglyceride-rich lipoprotein particle clearance | 8.52E-09 | 1.53E-07 | 40.00 | 4.00 | [APOC1, APOC2, APOC4-APOC2, APOE] |
| GO:0034382 | chylomicron remnant clearance | 8.52E-09 | 1.53E-07 | 40.00 | 4.00 | [APOC1, APOC2, APOC4-APOC2, APOE] |
| GO:0034377 | plasma lipoprotein particle assembly | 2.58E-08 | 4.39E-07 | 14.71 | 5.00 | [APOC1, APOC2, APOC4, APOC4-APOC2, APOE] |
| GO:0065005 | protein-lipid complex assembly | 4.62E-08 | 7.40E-07 | 13.16 | 5.00 | [APOC1, APOC2, APOC4, APOC4-APOC2, APOE] |
| GO:0071827 | plasma lipoprotein particle organization | 2.82E-07 | 4.24E-06 | 9.26 | 5.00 | [APOC1, APOC2, APOC4, APOC4-APOC2, APOE] |
| GO:0071825 | protein-lipid complex subunit organization | 4.06E-07 | 5.68E-06 | 8.62 | 5.00 | [APOC1, APOC2, APOC4, APOC4-APOC2, APOE] |
| GO:0034381 | plasma lipoprotein particle clearance | 4.43E-07 | 5.75E-06 | 8.47 | 5.00 | [APOC1, APOC2, APOC4, APOC4-APOC2, APOE] |
| GO:0001916 | positive regulation of T cell mediated cytotoxicity | 1.59E-06 | 1.91E-05 | 12.12 | 4.00 | [HLA-A, HLA-G, HLA-H, NECTIN2] |
| GO:0001914 | regulation of T cell mediated cytotoxicity | 3.50E-06 | 3.85E-05 | 10.00 | 4.00 | [HLA-A, HLA-G, HLA-H, NECTIN2] |
| GO:0055090 | acylglycerol homeostasis | 4.27E-06 | 4.27E-05 | 9.52 | 4.00 | [APOC2, APOC4, APOC4-APOC2, APOE] |
| GO:0070328 | triglyceride homeostasis | 4.27E-06 | 4.27E-05 | 9.52 | 4.00 | [APOC2, APOC4, APOC4-APOC2, APOE] |
| GO:0097006 | regulation of plasma lipoprotein particle levels | 5.29E-06 | 4.76E-05 | 5.15 | 5.00 | [APOC1, APOC2, APOC4, APOC4-APOC2, APOE] |
| GO:0001913 | T cell mediated cytotoxicity | 7.98E-06 | 6.38E-05 | 8.16 | 4.00 | [HLA-A, HLA-G, HLA-H, NECTIN2] |
| GO:0001912 | positive regulation of leukocyte mediated cytotoxicity | 1.68E-05 | 1.01E-04 | 6.78 | 4.00 | [HLA-A, HLA-G, HLA-H, NECTIN2] |
| GO:0002711 | positive regulation of T cell mediated immunity | 1.46E-05 | 1.03E-04 | 7.02 | 4.00 | [HLA-A, HLA-G, HLA-H, NECTIN2] |
| GO:0031341 | regulation of cell killing | 1.34E-04 | 1.34E-04 | 4.00 | 4.00 | [HLA-A, HLA-G, HLA-H, NECTIN2] |
| GO:0042590 | antigen processing and presentation of exogenous peptide antigen via MHC class I | 7.12E-05 | 1.42E-04 | 4.71 | 4.00 | [HLA-A, HLA-G, HLA-H, PSMC3] |
| GO:0031343 | positive regulation of cell killing | 3.32E-05 | 1.66E-04 | 5.71 | 4.00 | [HLA-A, HLA-G, HLA-H, NECTIN2] |
| GO:0001910 | regulation of leukocyte mediated cytotoxicity | 5.89E-05 | 1.77E-04 | 4.94 | 4.00 | [HLA-A, HLA-G, HLA-H, NECTIN2] |
| GO:0002709 | regulation of T cell mediated immunity | 5.89E-05 | 1.77E-04 | 4.94 | 4.00 | [HLA-A, HLA-G, HLA-H, NECTIN2] |
| GO:0002479 | antigen processing and presentation of exogenous peptide antigen via MHC class I, TAP-dependent | 5.61E-05 | 2.25E-04 | 5.00 | 4.00 | [HLA-A, HLA-G, HLA-H, PSMC3] |

## Supplementary Table12. FDA approved Alzheimer’s Disease drugs and insomnia drugs and their target genes obtained from Therapeutic Target Database and DrugBank

| Trait\Disease | drug | fda approved | DrugBank ID | Target gene |
| --- | --- | --- | --- | --- |
| Alzheimer's disease | Memantine | Y | DB01043 | GRIN1,DRD2,HTR3A,CHRNA7 |
|  | Ergoloid mesylate | Y | DB01049 | Not Available (pathways: Dopamine receptor; Alpha adrenergic receptor; Beta adrenergic receptor; Serotonin receptors) |
|  | Huperzine A | Y | DB04864 | ACHE |
|  | Tacrine | Y | DB00382 | ACHE, BCHE,CES1 |
|  | Donepezil | Y | DB00843 | ACHE, HTR2A,BCHE,NOS1,TNFAIP6,IL1B |
|  | Dihydroergotoxine | Y | DB16602 | Not Available |
|  | Galantamine | Y | DB00674 | ACHE, CHRNA7, BCHE |
|  | Rivastigmine | Y | DB00989 | ACHE, BCHE |
| Insomnia | Gaboxadol | Y | DB06554 | Not Available |
|  | Suvorexant | Y | DB09034 | HCRTR1, HCRTR2 |
|  | Secobarbital | Y | DB00418 | GABRA1,GABRA2,GABRA3,GABRA4,GABRA5,GABRA6,CHRNA4,CHRNA7,GRIA2,GRIK2 |
|  | Quazepam | Y | DB01589 | Not Available |
|  | Ethchlorvynol | Y | DB00189 | Not Available |
|  | Temazepam | Y | DB00231 | Not Available |
|  | Methyprylon | Y | DB01107 | GABRA1 |
|  | Aprobarbital | Y | DB01352 | GABRA1,GABRA2,GABRA3,GABRA4,GABRA5,GABRA6,CHRNA4,CHRNA7,GRIA2,GRIK2 |
|  | Eszopiclone | Y | DB00402 | GABRA1,GABRA2,GABRA3,GABRA5 |
|  | Flurazepam | Y | DB00690 | Not Available |
|  | Zaleplon | Y | DB00962 | GABRA1 |
|  | Butabarbital | Y | DB00237 | GRIA1,GRIA2,GRIA3,GRIA4,GRIK1,GRIK2,GRIK3,GRIK4,GRIK5, |
|  | Melatonin | Y | DB01065 | MTNR1A,MTNR1B,ESR1,RORB,CALM1,MPO,EPX,CALR,ASMT,NQO2, |
|  | Ethinamate | Y | DB01031 | CA1,CA2 |
|  | Barbital | Y | DB01483 | GRIA1,GRIA2,GRIA3,GRIA4,GRIK1,GRIK2,GRIK3,GRIK4,GRIK5, |
|  | Pentobarbital | Y | DB00312 | GABRA1,GABRA2,GABRA3,GABRA4,GABRA5,GABRA6,CHRNA4,CHRNA7,GRIA2,GRIK2,NR1I2 |
|  | Indiplon | Y | DB12590 | Not Available |
|  | Butobarbital | Y | DB01353 | GABRA1,GABRA2,GABRA3,GABRA4,GABRA5,GABRA6,CHRNA4,CHRNA7,GRIA2,GRIK2 |
|  | Estazolam | Y | DB01215 | Not Available |
|  | Tasimelteon | Y | DB09071 | MTNR1A,MTNR1B |
|  | Amobarbital | Y | DB01351 | GABRA1,GABRA2,GABRA3,GABRA4,GABRA5,GABRA6,CHRNA4,CHRNA7,GRIA2,GRIK2 |
|  | Zolpidem | Y | DB00425 | GABRA1,GABRA2,GABRA3,GABRG2 |
|  | Nitrazepam | Y | DB01595 | SCN1A |
|  | Ramelteon | Y | DB00980 | MTNR1A,MTNR1B |
|  | Triazolam | Y | DB00897 | Not Available |
|  | Flunitrazepam | Y | DB01544 | Not Available |
|  | Glutethimide | Y | DB01437 | GABRA1 |
|  | Zopiclone | Y | DB01198 | GABRA1,GABRA2,GABRA3,GABRA5,TSPO |

## Supplementary Table13. Horizontal pleiotropy test, steiger directionality test and heterogeneity test in the bidirectional MR analysis between AD and sleep-related phenotypes.

|  |  | horizontal pleiotropy test | | | steiger directionality test | | | | heterogeneity test | | |
| --- | --- | --- | --- | --- | --- | --- | --- | --- | --- | --- | --- |
| exposure | **outcome** | **egger_intercept** | **se** | **pleiotropy_pval** | **snp_r2.exposure** | **snp_r2.outcome** | **correct_causal_direction** | **steiger_pval** | **method** | **Q_df** | **heterogeneity_pval** |
| AlzheimersDementia | Insomnia | 0.000664 | 0.001912 | 7.31E-01 | 0.008376 | 0.000107 | TRUE | 5.3E-292 | MR Egger | 28 | 6.34E-02 |
| Insomnia | AlzheimersDementia | 0.000561 | 0.002572 | 8.31E-01 | 0.001399 | 3.69E-05 | TRUE | 4.57E-45 | MR Egger | 11 | 2.01E-01 |
| AlzheimersDementia | Sleepdur | 0.00131 | 0.000766 | 9.82E-02 | 0.008376 | 7.38E-05 | TRUE | 1.1E-303 | MR Egger | 28 | 9.83E-01 |
| Sleepdur | AlzheimersDementia | 0.002039 | 0.001683 | 2.32E-01 | 0.004916 | 0.000154 | TRUE | 1.6E-147 | MR Egger | 45 | 4.31E-02 |
| AlzheimersDementia | Snoring | 0.002771 | 0.002153 | 2.09E-01 | 0.008376 | 0.000171 | TRUE | 7.8E-263 | MR Egger | 28 | 2.82E-01 |
| Snoring | AlzheimersDementia | 0.001382 | 0.002941 | 6.42E-01 | 0.003563 | 0.000119 | TRUE | 1.1E-102 | MR Egger | 31 | 3.29E-02 |

## Supplementary Table 14 Shared TWAS significant genes between AD and Insomnia across 48 GTEx tissues.

| Gene.ID | CHR | Tissue_AD | TWAS.Z_AD | BH_AD | Tissue_Insomnia | TWAS.Z_Insomnia | BH_Insomnia |
| --- | --- | --- | --- | --- | --- | --- | --- |
| CNIH2 | 11 | Heart_Atrial_Appendage | -4.568065 | 0.01479641 | Artery_Aorta | 5.429 | 0.00029752 |
|  | 11 | Lung | -4.52029 | 0.0221862 | Skin_Not_Sun_Exposed_Suprapubic | 5.355 | 0.00148396 |
|  | 11 |  |  |  | Spleen | 5.14 | 0.00211008 |
| MUS81 | 11 | Esophagus_Muscularis | 4.728 | 0.00942504 | Colon_Sigmoid | -5.60152 | 0.00032224 |
| NR1H3 | 11 | Artery_Tibial | -4.33 | 0.04130528 | Whole_Blood | -4.366216 | 0.03504816 |
| PACSIN3 | 11 | Brain_Spinal_cord_cervical_c-1 | 4.289 | 0.0466116 | Esophagus_Gastroesophageal_Junction | 4.58982 | 0.0221926 |
|  | 11 | Heart_Atrial_Appendage | 4.387 | 0.02161569 |  |  |  |
| PTPMT1 | 11 | Cells_Transformed_fibroblasts | 4.446 | 0.027531 | Artery_Tibial | 6.15949 | 1.21E-05 |
|  | 11 | Liver | 4.83036 | 0.00861084 |  |  |  |
| RP11-390K5.6 | 11 | Heart_Atrial_Appendage | 4.34 | 0.02372504 | Brain_Caudate_basal_ganglia | 5.163 | 0.00117839 |
| RP11-867G23.3 | 11 | Adipose_Visceral_Omentum | 4.563721 | 0.02734811 | Thyroid | 5.71954 | 0.00020227 |
|  | 11 | Heart_Atrial_Appendage | 4.160115 | 0.04781766 |  |  |  |
| SLC29A2 | 11 | Brain_Anterior_cingulate_cortex_BA24 | -4.41712 | 0.02262833 | Brain_Caudate_basal_ganglia | 5.04826 | 0.0016221 |
| SLC39A13 | 11 | Thyroid | -4.359647 | 0.0491504 | Adrenal_Gland | -5.55919 | 0.00039935 |
|  | 11 |  |  |  | Artery_Coronary | -4.503 | 0.01867022 |
|  | 11 |  |  |  | Muscle_Skeletal | -4.56575 | 0.03765876 |
|  | 11 |  |  |  | Whole_Blood | -4.438121 | 0.04204852 |

Abbreviations: CHR: chromosome; AD: Alzheimer’s disease; Genes in blue are statistically independent genetic effects from all of the shared TWAS genes between AD and Insomnia

## Supplementary Table 15 Shared TWAS significant genes between AD and Sleepdur across 48 GTEx tissues.

| Gene.ID | CHR | Tissue_AD | TWAS.Z_AD | BH_AD | Tissue_Sleepdur | TWAS.Z_Sleepdur | BH_Sleepdur |
| --- | --- | --- | --- | --- | --- | --- | --- |
| ENO3 | 17 | Vagina | 5.287 | 0.00082485 | Brain_Frontal_Cortex_BA9 | 4.46969 | 0.006838036875 |
| GNGT2 | 17 | Brain_Spinal_cord_cervical_c-1 | 4.278 | 0.0492156 | Brain_Amygdala | 4.336 | 0.0138642307692308 |
| MAFK | 7 | Heart_Atrial_Appendage | 4.418 | 0.02496142 | Prostate | -4.6917 | 0.01938734 |
| NR1H3 | 11 | Artery_Tibial | -4.33 | 0.04130528 | Ovary | 4.46904 | 0.027197565 |
| NUP160 | 11 | Artery_Tibial | -4.817 | 0.02428418 | Artery_Aorta | 4.47227 | 0.03046077 |
|  | 11 | Skin_Not_Sun_Exposed_Suprapubic | -4.817 | 0.00632764 | Vagina | 4.561506 | 0.0225281066666667 |
| PTPMT1 | 11 | Cells_Transformed_fibroblasts | 4.446 | 0.027531 | Vagina | 4.652926 | 0.02175204 |
|  | 11 | Liver | 4.83036 | 0.00861084 | Whole_Blood | -4.48216 | 0.02569503 |
| TMEM184A | 7 | Breast_Mammary_Tissue | -4.202 | 0.03235446 | Brain_Cortex | 4.46815 | 0.0292719 |
|  | 7 | Lung | 4.418 | 0.02554029 |  |  |  |
|  | 7 | Muscle_Skeletal | 4.418 | 0.01882938 |  |  |  |
| ZNF232 | 17 | Adrenal_Gland | -4.3798 | 0.03507168 | Brain_Putamen_basal_ganglia | -4.21354 | 0.0165231463414634 |
|  | 17 | Brain_Caudate_basal_ganglia | -4.75725 | 0.01425704 |  |  |  |
|  | 17 | Brain_Cerebellum | -4.63288 | 0.01972504 |  |  |  |
|  | 17 | Brain_Cortex | -4.49096 | 0.03507187 |  |  |  |
|  | 17 | Brain_Frontal_Cortex_BA9 | -5.005 | 0.00260364 |  |  |  |
|  | 17 | Brain_Putamen_basal_ganglia | -4.41656 | 0.04498333 |  |  |  |
|  | 17 | Brain_Spinal_cord_cervical_c-1 | -5.15175 | 0.00335916 |  |  |  |
|  | 17 | Colon_Transverse | -4.60089 | 0.01333896 |  |  |  |
|  | 17 | Liver | -4.87024 | 0.00702797 |  |  |  |
|  | 17 | Pancreas | -4.36813 | 0.04547813 |  |  |  |
|  | 17 | Thyroid | -4.36802 | 0.03938333 |  |  |  |
|  | 17 | Vagina | -4.38801 | 0.0379164 |  |  |  |

Abbreviations: CHR: chromosome; AD: Alzheimer’s disease; Sleepdur: sleep duration; Genes in blue are statistically independent genetic effects from all of the shared TWAS genes between AD and Sleepdur

## Supplementary Table 16 Shared TWAS significant genes between AD and Snoring across 48 GTEx tissues.

| Gene.ID | CHR | Tissue_AD | TWAS.Z_AD | BH_AD | Tissue_Snoring | TWAS.Z_Snoring | BH_Snoring |
| --- | --- | --- | --- | --- | --- | --- | --- |
| AC002310.12 | 16 | Artery_Aorta | 4.49463 | 0.0156745342857143 | Brain_Cerebellum | 4.22342 | 0.0127434580645161 |
|  | 16 |  |  |  | Brain_Hippocampus | -4.76782 | 0.004867992 |
|  | 16 |  |  |  | Cells_Transformed_fibroblasts | -5.747135 | 2.8569312e-05 |
| AC002310.17 | 16 | Small_Intestine_Terminal_Ileum | -4.525 | 0.0102900282352941 | Brain_Cerebellar_Hemisphere | -4.91667 | 0.00122776 |
|  | 16 |  |  |  | Small_Intestine_Terminal_Ileum | 4.762 | 0.0022242816 |
| AHSP | 16 | Brain_Caudate_basal_ganglia | -4.62 | 0.00798061714285714 | Brain_Caudate_basal_ganglia | 4.798 | 0.00258631111111111 |
|  | 16 | Whole_Blood | -4.653 | 0.00505324 | Whole_Blood | 4.772 | 0.00140625333333333 |
| ALDOA | 16 | Spleen | 4.28033 | 0.035871275 | Muscle_Skeletal | 4.21076 | 0.0175300909090909 |
|  | 16 |  |  |  | Pancreas | 4.271 | 0.014189175 |
| ARMC5 | 16 | Brain_Caudate_basal_ganglia | -4.2315 | 0.0225009066666667 | Adipose_Subcutaneous | 4.26306 | 0.0134205692307692 |
|  | 16 | Thyroid | -5.014517 | 0.002514232 | Artery_Tibial | 4.27156 | 0.0137310723404255 |
|  | 16 |  |  |  | Brain_Caudate_basal_ganglia | 4.70416 | 0.00337249090909091 |
|  | 16 |  |  |  | Cells_Transformed_fibroblasts | 3.883049 | 0.0395218536585366 |
|  | 16 |  |  |  | Nerve_Tibial | 5.81551 | 2.8298875e-05 |
|  | 16 |  |  |  | Pancreas | 5.769 | 3.866247e-05 |
|  | 16 |  |  |  | Skin_Sun_Exposed_Lower_leg | 5.28115 | 0.000256782222222222 |
|  | 16 |  |  |  | Thyroid | 5.39093 | 0.000189310057142857 |
| BCKDK | 16 | Adipose_Subcutaneous | -4.20893 | 0.04439418 | Adipose_Subcutaneous | 3.90563 | 0.0477575294117647 |
|  | 16 | Adipose_Visceral_Omentum | 4.201 | 0.04338726 | Esophagus_Gastroesophageal_Junction | 6.062 | 2.00933e-05 |
|  | 16 | Esophagus_Mucosa | 4.8237 | 0.00792702 | Esophagus_Mucosa | -5.16591 | 0.000366452181818182 |
|  | 16 | Esophagus_Muscularis | -4.447 | 0.02410928 | Esophagus_Muscularis | 4.927 | 0.00198109714285714 |
|  | 16 | Heart_Left_Ventricle | -4.635 | 0.00712572 | Heart_Left_Ventricle | 4.684 | 0.00436236888888889 |
|  | 16 | Lung | 4.75416 | 0.01786025 | Lung | -5.105707 | 0.001480875 |
|  | 16 | Ovary | 4.489364 | 0.0123530925 | Ovary | -4.90119 | 0.0013190473 |
|  | 16 | Pancreas | 4.22867 | 0.028499625 | Pancreas | -3.89691 | 0.0488780068965517 |
|  | 16 | Whole_Blood | 5.017171 | 0.001214632 | Stomach | 5.32078 | 0.000216903285714286 |
|  | 16 |  |  |  | Whole_Blood | -4.80427 | 0.0013473375 |
| BCL7C | 16 | Cells_EBV-transformed_lymphocytes | -4.55 | 0.0115704533333333 | Artery_Tibial | -6.47968 | 1.5285727e-06 |
|  | 16 | Uterus | -5.4911 | 0.00052312 | Cells_EBV-transformed_lymphocytes | 4.629 | 0.00316892266666667 |
|  | 16 |  |  |  | Esophagus_Gastroesophageal_Junction | -5.12916 | 0.000545443125 |
|  | 16 |  |  |  | Pituitary | 4.51 | 0.00613617882352941 |
|  | 16 |  |  |  | Uterus | 5.498747 | 0.000166526533333333 |
| C16orf58 | 16 | Lung | 4.413 | 0.024412 | Lung | -4.043 | 0.0357645283018868 |
|  | 16 | Ovary | 4.22754 | 0.0251267384615385 | Ovary | -4.99993 | 0.00099309175 |
|  | 16 | Skin_Not_Sun_Exposed_Suprapubic | 4.413872 | 0.0442068 | Skin_Not_Sun_Exposed_Suprapubic | -5.42 | 0.000112491377777778 |
| C16orf93 | 16 | Adrenal_Gland | 5.44239 | 0.0007751136 | Adrenal_Gland | -4.97713 | 0.000864065454545454 |
|  | 16 | Artery_Aorta | 5.98328 | 3.447498e-05 | Artery_Aorta | -5.53367 | 8.23831333333333e-05 |
|  | 16 | Brain_Anterior_cingulate_cortex_BA24 | 4.49428 | 0.0105297177777778 | Brain_Caudate_basal_ganglia | -4.54135 | 0.00542155466666667 |
|  | 16 | Brain_Caudate_basal_ganglia | 5.2419 | 0.000771044 | Brain_Cerebellar_Hemisphere | -5.40231 | 0.00025245815 |
|  | 16 | Brain_Cerebellar_Hemisphere | 4.88374 | 0.003192176 | Brain_Spinal_cord_cervical_c-1 | -5.51459 | 0.000113925 |
|  | 16 | Brain_Cortex | 4.844 | 0.0047117 | Cells_EBV-transformed_lymphocytes | -4.802 | 0.00254183 |
|  | 16 | Brain_Frontal_Cortex_BA9 | 5.570946 | 0.0003535169 | Minor_Salivary_Gland | -4.64744 | 0.00339825230769231 |
|  | 16 | Brain_Nucleus_accumbens_basal_ganglia | 4.328 | 0.0254858823529412 | Pancreas | -4.72202 | 0.00243243 |
|  | 16 | Breast_Mammary_Tissue | 4.37327 | 0.0322730666666667 | Uterus | -4.848938 | 0.00147424727272727 |
|  | 16 | Cells_EBV-transformed_lymphocytes | 4.561 | 0.0146501511111111 | |  |  |
|  | 16 | Esophagus_Mucosa | 4.249 | 0.0278937692307692 | |  |  |
|  | 16 | Minor_Salivary_Gland | 4.56431 | 0.01646787 |  |  |  |
|  | 16 | Pancreas | 5.31638 | 0.000514206 | |  |  |
|  | 16 | Thyroid | 4.287259 | 0.0380180444444444 | |  |  |
| CASS4 | 20 | Cells_Transformed_fibroblasts | -5.841 | 8.164908e-05 | Vagina | -4.819 | 0.01915776 |
|  | 20 | Whole_Blood | 5.841 | 7.218252e-05 | |  |  |
| CD2BP2 | 16 | Brain_Substantia_nigra | -4.38801 | 0.03477855 | Adipose_Visceral_Omentum | 4.73372 | 0.00358842 |
|  | 16 |  |  |  | Brain_Amygdala | 5.0657 | 0.00063237625 |
|  | 16 |  |  |  | Brain_Caudate_basal_ganglia | 3.95272 | 0.0488939304347826 |
|  | 16 |  |  |  | Brain_Cortex | 4.54067 | 0.00416262 |
|  | 16 |  |  |  | Brain_Substantia_nigra | 4.66055 | 0.00256263 |
|  | 16 |  |  |  | Cells_Transformed_fibroblasts | 4.47812 | 0.0047384784 |
|  | 16 |  |  |  | Heart_Atrial_Appendage | 3.9609 | 0.044930556 |
|  | 16 |  |  |  | Nerve_Tibial | 5.06402 | 0.000697372727272727 |
|  | 16 |  |  |  | Pituitary | 4.45833 | 0.00699839368421053 |
| CORO1A | 16 | Cells_Transformed_fibroblasts | 4.20371 | 0.0295536857142857 | Adipose_Subcutaneous | -4.271 | 0.01347372 |
|  | 16 |  |  |  | Adipose_Visceral_Omentum | -4.271 | 0.01987903125 |
|  | 16 |  |  |  | Colon_Sigmoid | -4.27 | 0.018525 |
|  | 16 |  |  |  | Lung | -4.326746 | 0.0123202272727273 |
|  | 16 |  |  |  | Skin_Not_Sun_Exposed_Suprapubic | -4.27 | 0.0126912603773585 |
|  | 16 |  |  |  | Skin_Sun_Exposed_Lower_leg | -4.272 | 0.0129728518518519 |
|  | 16 |  |  |  | Thyroid | -4.272 | 0.0141052923076923 |
| COX6A2 | 16 | Brain_Nucleus_accumbens_basal_ganglia | 4.541 | 0.0115536 | Brain_Nucleus_accumbens_basal_ganglia | -4.873 | 0.00158862 |
|  | 16 |  |  |  | Prostate | -5.304 | 0.000230972 |
| CTD-2574D22.2 | 16 | Adrenal_Gland | -4.057 | 0.04882528 | Brain_Cerebellum | 3.95607 | 0.0356877257142857 |
|  | 16 | Brain_Caudate_basal_ganglia | 4.573 | 0.00777509777777778 | Vagina | 4.271 | 0.0152604705882353 |
|  | 16 | Cells_EBV-transformed_lymphocytes | -4.21004 | 0.0366973333333333 | |  |  |
|  | 16 | Colon_Transverse | -4.204 | 0.04150604 |  |  |  |
| CTF1 | 16 | Brain_Cerebellar_Hemisphere | 4.55 | 0.00967763764705882 | Brain_Cerebellar_Hemisphere | -4.629 | 0.00321848514285714 |
|  | 16 | Brain_Frontal_Cortex_BA9 | 4.58492 | 0.00906248857142857 | Brain_Frontal_Cortex_BA9 | -6.17147 | 4.7298605e-06 |
|  | 16 | Brain_Hypothalamus | 4.2641 | 0.022456725 | Brain_Hippocampus | -4.27121 | 0.015866775 |
|  | 16 | Brain_Spinal_cord_cervical_c-1 | 4.447 | 0.0378014 | Brain_Spinal_cord_cervical_c-1 | -5.66901 | 6.2496e-05 |
|  | 16 | Cells_EBV-transformed_lymphocytes | -4.13422 | 0.04610912 | Cells_EBV-transformed_lymphocytes | 4.206 | 0.0153069090909091 |
|  | 16 | Lung | 4.18424 | 0.04667 | Minor_Salivary_Gland | 4.629 | 0.00344665428571429 |
|  | 16 | Minor_Salivary_Gland | -4.55 | 0.014094656 | Pancreas | -4.51 | 0.00554726117647059 |
|  | 16 | Pancreas | 4.804 | 0.00567567 | Uterus | 5.060139 | 0.000913280333333333 |
|  | 16 | Uterus | -4.7578 | 0.00732368 |  |  |  |
| DCTPP1 | 16 | Adipose_Visceral_Omentum | 5.09024 | 0.0014598345 | Adipose_Visceral_Omentum | -4.25114 | 0.0204367235294118 |
|  | 16 | Artery_Coronary | -4.3022 | 0.02354677 | Adrenal_Gland | 4.56244 | 0.00392442947368421 |
| FBRS | 16 | Brain_Hypothalamus | 4.413 | 0.0143948842105263 | Nerve_Tibial | -4.18695 | 0.0196108518518519 |
|  | 16 |  |  |  | Stomach | -4.20534 | 0.02137445 |
|  | 16 |  |  |  | Uterus | -4.943844 | 0.00111308311111111 |
| FBXL19 | 16 | Artery_Aorta | 4.55 | 0.0140628533333333 | Artery_Aorta | -4.629 | 0.00320961888888889 |
|  | 16 | Breast_Mammary_Tissue | 4.62 | 0.012189696 | Breast_Mammary_Tissue | -4.798 | 0.00181394285714286 |
|  | 16 |  |  |  | Cells_EBV-transformed_lymphocytes | 4.518 | 0.00505128 |
|  | 16 |  |  |  | Heart_Atrial_Appendage | 5.333 | 0.0002420957 |
|  | 16 |  |  |  | Heart_Left_Ventricle | 5.954946 | 1.81636e-05 |
| FBXL19-AS1 | 16 | Cells_EBV-transformed_lymphocytes | -5.58085 | 0.0001547764 | Adrenal_Gland | -3.89999 | 0.045729135483871 |
|  | 16 | Ovary | 5.228851 | 0.000788937 | Artery_Coronary | -4.07528 | 0.0291326363636364 |
|  | 16 |  |  |  | Cells_EBV-transformed_lymphocytes | 5.0223 | 0.0013236944 |
|  | 16 |  |  |  | Prostate | 5.769 | 5.701738e-05 |
| FUS | 16 | Adipose_Subcutaneous | -4.80247 | 0.005424036 | Adipose_Subcutaneous | 5.64609 | 0.0001416468 |
|  | 16 | Brain_Cortex | -5.1663 | 0.00118225333333333 | Brain_Amygdala | 5.2307 | 0.000300095714285714 |
|  | 16 | Brain_Spinal_cord_cervical_c-1 | -4.3973 | 0.035805 | Brain_Cortex | 5.94733 | 1.35044e-05 |
|  | 16 | Brain_Substantia_nigra | -4.265 | 0.0406766666666667 | Brain_Spinal_cord_cervical_c-1 | 5.84212 | 6.7053e-05 |
|  | 16 | Muscle_Skeletal | -4.38649 | 0.02174075 | Brain_Substantia_nigra | 5.858 | 1.903668e-05 |
|  | 16 | Spleen | -4.50289 | 0.0171363666666667 | Colon_Transverse | 4.32625 | 0.0100332666666667 |
|  | 16 |  |  |  | Muscle_Skeletal | 5.98864 | 1.603144e-05 |
|  | 16 |  |  |  | Nerve_Tibial | 5.63948 | 6.39882e-05 |
|  | 16 |  |  |  | Spleen | 5.30244 | 0.000249920571428571 |
| HIRIP3 | 16 | Adipose_Subcutaneous | 4.92372 | 0.0036664065 | Colon_Sigmoid | 4.67 | 0.004944 |
| HSD3B7 | 16 | Brain_Amygdala | -4.265 | 0.0331466666666667 | Adipose_Visceral_Omentum | 5.286 | 0.000407775 |
|  | 16 | Brain_Caudate_basal_ganglia | -4.3392 | 0.0160028 | Adrenal_Gland | 5.24751 | 0.0004538688 |
|  | 16 | Brain_Cerebellar_Hemisphere | -5.41313 | 0.00047498965 | Artery_Aorta | 5.214 | 0.000264751818181818 |
|  | 16 | Brain_Cerebellum | -4.631 | 0.01491672 | Brain_Amygdala | 5.858 | 3.87816e-05 |
|  | 16 | Brain_Frontal_Cortex_BA9 | -4.413 | 0.01425246 | Brain_Caudate_basal_ganglia | 5.968 | 1.74576e-05 |
|  | 16 | Esophagus_Mucosa | -4.1571 | 0.0387918 | Brain_Cerebellar_Hemisphere | 4.32722 | 0.00799102413793103 |
|  | 16 | Esophagus_Muscularis | -4.80358 | 0.00863616 | Brain_Cerebellum | 4.701 | 0.002653455 |
|  | 16 | Whole_Blood | 4.561 | 0.00643561090909091 | Brain_Cortex | 4.59724 | 0.00352862222222222 |
|  | 16 |  |  |  | Brain_Frontal_Cortex_BA9 | 4.043 | 0.032077147826087 |
|  | 16 |  |  |  | Breast_Mammary_Tissue | 5.206 | 0.000510549333333333 |
|  | 16 |  |  |  | Cells_EBV-transformed_lymphocytes | 4.6783 | 0.00311927333333333 |
|  | 16 |  |  |  | Cells_Transformed_fibroblasts | 4.314399 | 0.00898971428571428 |
|  | 16 |  |  |  | Colon_Transverse | 5.59325 | 7.065532e-05 |
|  | 16 |  |  |  | Esophagus_Mucosa | 5.47067 | 0.0001079424 |
|  | 16 |  |  |  | Esophagus_Muscularis | 4.33974 | 0.01583296 |
|  | 16 |  |  |  | Heart_Atrial_Appendage | 4.2847 | 0.0144830052631579 |
|  | 16 |  |  |  | Heart_Left_Ventricle | 5.333 | 0.0002249492 |
|  | 16 |  |  |  | Liver | 4.82914 | 0.00216853875 |
|  | 16 |  |  |  | Minor_Salivary_Gland | 5.03269 | 0.000909090285714286 |
|  | 16 |  |  |  | Muscle_Skeletal | 5.69 | 4.80187e-05 |
|  | 16 |  |  |  | Nerve_Tibial | 4.47267 | 0.00722206 |
|  | 16 |  |  |  | Pancreas | 5.333 | 0.00028116396 |
|  | 16 |  |  |  | Pituitary | 5.32922 | 0.0007936314 |
|  | 16 |  |  |  | Prostate | 5.69 | 4.54279e-05 |
|  | 16 |  |  |  | Skin_Not_Sun_Exposed_Suprapubic | 5.21 | 0.000304168 |
|  | 16 |  |  |  | Skin_Sun_Exposed_Lower_leg | 5.54415 | 6.65778125e-05 |
|  | 16 |  |  |  | Small_Intestine_Terminal_Ileum | 5.189 | 0.0006110982 |
|  | 16 |  |  |  | Stomach | 5.69 | 4.6802675e-05 |
|  | 16 |  |  |  | Vagina | 5.69 | 0.0001689608 |
|  | 16 |  |  |  | Whole_Blood | -4.718 | 0.001655052 |
| INO80E | 16 | Adipose_Subcutaneous | 4.23727 | 0.04879905 | Brain_Putamen_basal_ganglia | -3.973008 | 0.0456259523809524 |
|  | 16 | Adipose_Visceral_Omentum | 4.12496 | 0.0465490846153846 | |  |  |
|  | 16 | Artery_Aorta | 4.36939 | 0.024596875 | |  |  |
|  | 16 | Artery_Coronary | 4.96951 | 0.00155817383333333 | |  |  |
|  | 16 | Artery_Tibial | 4.26854 | 0.0409587625 | |  |  |
|  | 16 | Brain_Anterior_cingulate_cortex_BA24 | 4.087 | 0.0359584787878788 | |  |  |
|  | 16 | Brain_Caudate_basal_ganglia | 4.132 | 0.029096 |  |  |  |
|  | 16 | Brain_Cortex | 4.7535 | 0.005936 |  |  |  |
|  | 16 | Cells_Transformed_fibroblasts | 4.93144 | 0.004279104 | |  |  |
|  | 16 | Esophagus_Gastroesophageal_Junction | 4.79822 | 0.00799733333333333 | |  |  |
|  | 16 | Esophagus_Mucosa | 4.4709 | 0.013138614 | |  |  |
|  | 16 | Esophagus_Muscularis | 4.54901 | 0.017903424 | |  |  |
|  | 16 | Lung | 4.43135 | 0.028002 |  |  |  |
|  | 16 | Ovary | 4.448568 | 0.01328736 |  |  |  |
|  | 16 | Pancreas | 4.17586 | 0.03087315 |  |  |  |
| ITGAL | 16 | Brain_Anterior_cingulate_cortex_BA24 | -4.38657 | 0.0130112916666667 | Adrenal_Gland | -5.52725 | 0.00015964 |
|  | 16 | Esophagus_Mucosa | 4.804 | 0.00657774 | Artery_Coronary | -4.665 | 0.00391390636363636 |
|  | 16 | Heart_Left_Ventricle | -4.06426 | 0.0481036 | Brain_Cerebellum | -4.48352 | 0.00523118608695652 |
|  | 16 |  |  |  | Colon_Sigmoid | -4.67 | 0.004944 |
|  | 16 |  |  |  | Esophagus_Mucosa | -4.51 | 0.00520436571428571 |
|  | 16 |  |  |  | Uterus | 4.952714 | 0.001196637 |
| ITGAM | 16 | Brain_Cerebellum | -4.385353 | 0.0211274666666667 | Brain_Cerebellar_Hemisphere | 4.95171 | 0.0011295392 |
|  | 16 | Brain_Hypothalamus | -4.283 | 0.0224262545454545 | Brain_Cerebellum | 5.10487 | 0.000678219 |
|  | 16 | Esophagus_Mucosa | -4.413 | 0.0143361 | Brain_Hypothalamus | 4.72615 | 0.005117005 |
|  | 16 | Muscle_Skeletal | 4.1991 | 0.0337769333333333 | Breast_Mammary_Tissue | -4.01162 | 0.03190272 |
|  | 16 |  |  |  | Colon_Transverse | -5.233 | 0.000440935666666667 |
|  | 16 |  |  |  | Esophagus_Mucosa | 4.043 | 0.02968416 |
|  | 16 |  |  |  | Muscle_Skeletal | -5.35084 | 0.000147038888888889 |
| ITGAX | 16 | Brain_Caudate_basal_ganglia | 4.4967 | 0.00912556363636364 | Adipose_Subcutaneous | -5.05341 | 0.000832990666666667 |
|  | 16 | Colon_Transverse | 4.15356 | 0.0450463826086957 | Brain_Caudate_basal_ganglia | -4.77165 | 0.002662284 |
|  | 16 | Esophagus_Gastroesophageal_Junction | 4.57892 | 0.0175066625 | Brain_Cerebellar_Hemisphere | 4.27398 | 0.00982208 |
|  | 16 | Skin_Sun_Exposed_Lower_leg | 4.84019 | 0.00782383333333333 | Colon_Transverse | -4.88429 | 0.00126736 |
|  | 16 |  |  |  | Esophagus_Gastroesophageal_Junction | -5.44844 | 0.0001523492 |
|  | 16 |  |  |  | Skin_Sun_Exposed_Lower_leg | -4.98561 | 0.0009298325 |
| KAT8 | 16 | Adipose_Subcutaneous | -6.01095 | 3.178416e-05 | Adipose_Subcutaneous | 5.46742 | 0.00015788436 |
|  | 16 | Adipose_Visceral_Omentum | -5.73137 | 0.00016245756 | Adipose_Visceral_Omentum | 5.57645 | 0.0001332065 |
|  | 16 | Adrenal_Gland | -4.62 | 0.01414656 | Adrenal_Gland | 4.798 | 0.00168411428571429 |
|  | 16 | Artery_Aorta | -5.70919 | 8.97294e-05 | Artery_Aorta | 5.35563 | 0.000149199177777778 |
|  | 16 | Artery_Coronary | -5.92254 | 4.416761e-05 | Artery_Coronary | 5.1122 | 0.000738449 |
|  | 16 | Artery_Tibial | -5.90755 | 5.771651e-05 | Artery_Tibial | 5.44127 | 0.000146647616666667 |
|  | 16 | Brain_Amygdala | -4.55 | 0.0166562 | Brain_Amygdala | 4.754 | 0.00190274615384615 |
|  | 16 | Brain_Anterior_cingulate_cortex_BA24 | -5.77984 | 0.00010155596 | Brain_Anterior_cingulate_cortex_BA24 | 5.40171 | 0.00011201025 |
|  | 16 | Brain_Caudate_basal_ganglia | -5.4981 | 0.0002793216 | Brain_Caudate_basal_ganglia | 5.68245 | 4.83721e-05 |
|  | 16 | Brain_Cerebellar_Hemisphere | -4.55 | 0.00967763764705882 | Brain_Cerebellar_Hemisphere | 4.629 | 0.00321848514285714 |
|  | 16 | Brain_Cerebellum | -5.804766 | 0.0001057284 | Brain_Cerebellum | 5.40904 | 0.0002598132 |
|  | 16 | Brain_Cortex | -6.1445 | 1.190168e-05 | Brain_Cortex | 5.28327 | 0.000209408888888889 |
|  | 16 | Brain_Frontal_Cortex_BA9 | -5.102908 | 0.0011667455 | Brain_Frontal_Cortex_BA9 | 4.25236 | 0.0155909263157895 |
|  | 16 | Brain_Hippocampus | -5.491 | 0.00034896 | Brain_Hippocampus | 4.605 | 0.00634286117647059 |
|  | 16 | Brain_Hypothalamus | -5.8647 | 6.03315e-05 | Brain_Hypothalamus | 5.46929 | 0.0003029982 |
|  | 16 | Brain_Nucleus_accumbens_basal_ganglia | -5.6709 | 0.0002050764 | Brain_Nucleus_accumbens_basal_ganglia | 5.33951 | 0.0004486648 |
|  | 16 | Brain_Putamen_basal_ganglia | -5.5378 | 0.0002064735 | Brain_Putamen_basal_ganglia | 5.374385 | 0.000345472 |
|  | 16 | Brain_Substantia_nigra | -5.64487 | 0.0002013495 | Brain_Spinal_cord_cervical_c-1 | 5.769 | 5.18847e-05 |
|  | 16 | Breast_Mammary_Tissue | -6.00351 | 3.063296e-05 | Brain_Substantia_nigra | 5.44817 | 0.000103522116666667 |
|  | 16 | Cells_EBV-transformed_lymphocytes | -6.01591 | 2.318408e-05 | Breast_Mammary_Tissue | 5.59781 | 6.888448e-05 |
|  | 16 | Cells_Transformed_fibroblasts | -5.95326 | 4.137516e-05 | Cells_EBV-transformed_lymphocytes | 5.4226 | 0.0002538592 |
|  | 16 | Colon_Sigmoid | -6.01481 | 2.736e-05 | Cells_Transformed_fibroblasts | 5.266036 | 0.000364458 |
|  | 16 | Colon_Transverse | -5.491 | 0.00063368 | Colon_Sigmoid | 5.57 | 0.00018924 |
|  | 16 | Esophagus_Gastroesophageal_Junction | -5.92303 | 4.73842e-05 | Colon_Transverse | 4.605 | 0.003263452 |
|  | 16 | Esophagus_Mucosa | -5.8721 | 7.25238e-05 | Esophagus_Gastroesophageal_Junction | 5.72017 | 3.973675e-05 |
|  | 16 | Esophagus_Muscularis | -5.90569 | 5.829408e-05 | Esophagus_Mucosa | 5.40787 | 0.00013450635 |
|  | 16 | Heart_Atrial_Appendage | -5.89476 | 5.638875e-05 | Esophagus_Muscularis | 5.47141 | 0.0001851792 |
|  | 16 | Heart_Left_Ventricle | -5.59382 | 0.0003101784 | Heart_Atrial_Appendage | 5.5626 | 0.000133328066666667 |
|  | 16 | Liver | -6.1741 | 8.420895e-06 | Heart_Left_Ventricle | 5.450184 | 0.0001756979 |
|  | 16 | Lung | -5.99791 | 3.59e-05 | Liver | 5.4527 | 0.0002093616 |
|  | 16 | Minor_Salivary_Gland | -5.77051 | 0.0001038692 | Lung | 4.858772 | 0.002647625 |
|  | 16 | Muscle_Skeletal | -5.77669 | 0.00011524488 | Minor_Salivary_Gland | 5.66325 | 9.79526e-05 |
|  | 16 | Nerve_Tibial | -5.86 | 8.88725e-05 | Muscle_Skeletal | 5.50307 | 9.40208666666667e-05 |
|  | 16 | Ovary | -5.684068 | 0.0001827012 | Nerve_Tibial | 5.41306 | 0.000193336666666667 |
|  | 16 | Pancreas | -5.88625 | 5.748435e-05 | Ovary | 5.31623 | 0.0002934292 |
|  | 16 | Pituitary | -5.499 | 0.0006149436 | Pancreas | 5.45569 | 0.0001775466 |
|  | 16 | Prostate | -5.3904 | 0.0010058524 | Pituitary | 4.64 | 0.00509282181818182 |
|  | 16 | Skin_Not_Sun_Exposed_Suprapubic | -5.826506 | 9.812176e-05 | Prostate | 4.985708 | 0.000802548727272727 |
|  | 16 | Skin_Sun_Exposed_Lower_leg | -5.66386 | 0.000267214 | Skin_Not_Sun_Exposed_Suprapubic | 5.5 | 9.65862857142857e-05 |
|  | 16 | Small_Intestine_Terminal_Ileum | -6.105 | 1.491543e-05 | Skin_Sun_Exposed_Lower_leg | 5.5856 | 7.01135833333333e-05 |
|  | 16 | Spleen | -5.66603 | 0.0002240516 | Small_Intestine_Terminal_Ileum | 5.6354 | 0.00012670875 |
|  | 16 | Stomach | -4.3031 | 0.0353784 | Spleen | 5.2179 | 0.000308625111111111 |
|  | 16 | Testis | -5.439 | 0.0012423944 | Stomach | 4.90038 | 0.001282467 |
|  | 16 | Thyroid | -5.759207 | 0.0001597388 | Testis | 4.842 | 0.001868806875 |
|  | 16 | Uterus | -4.541 | 0.0122061333333333 | Thyroid | 5.28927 | 0.000258354666666667 |
|  | 16 | Vagina | -5.0337 | 0.00213307466666667 | Uterus | 4.873 | 0.00143858 |
|  | 16 | Whole_Blood | -5.232563 | 0.001161318 | Vagina | 5.35733 | 0.000374729333333333 |
|  | 16 |  |  |  | Whole_Blood | 5.43429 | 0.000109277142857143 |
| MAPK3 | 16 | Brain_Hypothalamus | 4.573 | 0.00921252428571429 | Brain_Cerebellar_Hemisphere | -4.14693 | 0.0166836741935484 |
|  | 16 |  |  |  | Brain_Cerebellum | -4.28855 | 0.0101743448275862 |
|  | 16 |  |  |  | Heart_Left_Ventricle | -4.248858 | 0.0176704705882353 |
|  | 16 |  |  |  | Pancreas | -4.37756 | 0.00919136842105263 |
|  | 16 |  |  |  | Testis | -4.46498 | 0.0074265516 |
| MIR4519 | 16 | Brain_Hypothalamus | -4.201 | 0.0274327846153846 | Artery_Aorta | -6.1253 | 1.424651e-05 |
|  | 16 | Cells_EBV-transformed_lymphocytes | 4.113 | 0.0460384727272727 | Brain_Cerebellum | -4.3042 | 0.0098352 |
|  | 16 |  |  |  | Cells_EBV-transformed_lymphocytes | -5.94 | 1.84566e-05 |
| MYLPF | 16 | Cells_Transformed_fibroblasts | 4.58446 | 0.01431612 | Thyroid | 4.44414 | 0.00694722 |
| PHKG2 | 16 | Adipose_Visceral_Omentum | 4.10333 | 0.0474184071428571 | Brain_Cortex | -5.286 | 0.000231875 |
|  | 16 | Adrenal_Gland | 4.372 | 0.01812528 | Brain_Frontal_Cortex_BA9 | -5.63146 | 5.002334e-05 |
|  | 16 | Brain_Hypothalamus | 4.8522 | 0.00545218 | Brain_Hypothalamus | -4.54898 | 0.00656943 |
|  | 16 | Esophagus_Muscularis | 4.201 | 0.0401611636363636 | Brain_Nucleus_accumbens_basal_ganglia | -5.93863 | 4.144854e-05 |
|  | 16 | Whole_Blood | -4.328 | 0.0160476923076923 | Cells_EBV-transformed_lymphocytes | -4.2276 | 0.014555580952381 |
| PRR14 | 16 | Artery_Aorta | 4.19467 | 0.0477507333333333 | Brain_Nucleus_accumbens_basal_ganglia | 4.696 | 0.003189275 |
|  | 16 | Brain_Anterior_cingulate_cortex_BA24 | 4.372 | 0.0128459307692308 | Brain_Putamen_basal_ganglia | -6.327735 | 3.360255e-06 |
|  | 16 | Brain_Nucleus_accumbens_basal_ganglia | -4.714 | 0.007018812 | Brain_Substantia_nigra | -5.858 | 1.903668e-05 |
|  | 16 | Brain_Putamen_basal_ganglia | 4.6562 | 0.0144846333333333 | Breast_Mammary_Tissue | -5.69 | 5.03936e-05 |
|  | 16 | Brain_Substantia_nigra | 4.265 | 0.0406766666666667 | Esophagus_Gastroesophageal_Junction | -4.73201 | 0.00195817058823529 |
|  | 16 | Cells_Transformed_fibroblasts | 4.086 | 0.0406255764705882 | Minor_Salivary_Gland | -4.84978 | 0.00203794 |
|  | 16 | Colon_Transverse | 4.249 | 0.042575375 | Skin_Not_Sun_Exposed_Suprapubic | -6.29 | 5.512848e-06 |
|  | 16 | Esophagus_Mucosa | 4.7736 | 0.006105492 | |  |  |
|  | 16 | Heart_Left_Ventricle | 5.2273 | 0.001201592 | |  |  |
|  | 16 | Minor_Salivary_Gland | 5.65396 | 0.0001032118 | |  |  |
|  | 16 | Skin_Not_Sun_Exposed_Suprapubic | 4.451811 | 0.0491764533333333 | |  |  |
| PRRT2 | 16 | Esophagus_Gastroesophageal_Junction | 4.49654 | 0.0172692416666667 | Esophagus_Mucosa | -4.665 | 0.00274294421052632 |
| PRSS36 | 16 | Adipose_Subcutaneous | 4.18636 | 0.0444412909090909 | Adipose_Subcutaneous | -4.25161 | 0.0130788857142857 |
|  | 16 | Brain_Amygdala | -4.265 | 0.0331466666666667 | Artery_Coronary | -4.54834 | 0.00538410928571429 |
|  | 16 | Brain_Anterior_cingulate_cortex_BA24 | -5.499 | 0.0002593207 | Brain_Amygdala | 5.858 | 3.87816e-05 |
|  | 16 | Brain_Caudate_basal_ganglia | -5.499 | 0.0005557336 | Brain_Anterior_cingulate_cortex_BA24 | 4.64 | 0.00277929176470588 |
|  | 16 | Brain_Cerebellar_Hemisphere | -5.499 | 0.0005862554 | Brain_Caudate_basal_ganglia | 4.64 | 0.00361621714285714 |
|  | 16 | Brain_Cerebellum | -5.488412 | 0.0003327576 | Brain_Cerebellar_Hemisphere | 4.64 | 0.0033379725 |
|  | 16 | Brain_Cortex | -5.51 | 0.000266378 | Brain_Cerebellum | 4.8113 | 0.00175628571428571 |
|  | 16 | Brain_Frontal_Cortex_BA9 | -5.439 | 0.000249650933333333 | Brain_Cortex | 4.74306 | 0.0022366 |
|  | 16 | Brain_Hippocampus | -5.439 | 0.0002338032 | Brain_Frontal_Cortex_BA9 | 4.842 | 0.00163865181818182 |
|  | 16 | Brain_Hypothalamus | -5.5616 | 0.00017898345 | Brain_Hippocampus | 4.842 | 0.004220235 |
|  | 16 | Brain_Nucleus_accumbens_basal_ganglia | -5.499 | 0.0001838948 | Brain_Hypothalamus | 5.19485 | 0.00068710875 |
|  | 16 | Brain_Putamen_basal_ganglia | -5.5483 | 0.0003900055 | Brain_Nucleus_accumbens_basal_ganglia | 4.64 | 0.00386601230769231 |
|  | 16 | Cells_EBV-transformed_lymphocytes | -4.561 | 0.0146501511111111 | Brain_Putamen_basal_ganglia | 4.933794 | 0.00120855222222222 |
|  | 16 | Cells_Transformed_fibroblasts | -5.491 | 0.00031464 | Cells_EBV-transformed_lymphocytes | 4.718 | 0.003082576 |
|  | 16 | Liver | -5.5443 | 0.00018677925 | Cells_Transformed_fibroblasts | 4.605 | 0.00341136 |
|  | 16 | Muscle_Skeletal | -5.491 | 0.00030248 | Liver | 4.58765 | 0.00472752 |
|  | 16 | Ovary | -4.244 | 0.0253751666666667 | Muscle_Skeletal | 4.605 | 0.003115544 |
|  | 16 | Pancreas | -5.491 | 0.00029106 | Ovary | 5.806 | 2.95274666666667e-05 |
|  | 16 |  |  |  | Pancreas | 4.605 | 0.0037473975 |
| PRSS53 | 16 | Adipose_Subcutaneous | 4.413 | 0.0293658 | Adipose_Subcutaneous | -4.043 | 0.0294215225806452 |
|  | 16 | Adrenal_Gland | 4.413 | 0.0176832 | Adrenal_Gland | -4.043 | 0.0273003789473684 |
|  | 16 | Artery_Coronary | 4.413 | 0.017764575 | Artery_Aorta | -4.09548 | 0.026509528 |
|  | 16 | Artery_Tibial | 5.25671 | 0.0012225255 | Artery_Coronary | -4.043 | 0.0306526 |
|  | 16 | Brain_Amygdala | 4.561 | 0.0210895666666667 | Artery_Tibial | -5.64365 | 6.902695e-05 |
|  | 16 | Brain_Cerebellum | -4.413 | 0.0278664 | Brain_Amygdala | -4.718 | 0.0018489625 |
|  | 16 | Breast_Mammary_Tissue | 4.206 | 0.0458524444444444 | Brain_Cerebellum | 4.043 | 0.0262272 |
|  | 16 | Cells_Transformed_fibroblasts | 4.52792 | 0.0133946742857143 | Cells_Transformed_fibroblasts | -4.155652 | 0.0145633371428571 |
|  | 16 | Colon_Transverse | 4.7122 | 0.009703225 | Colon_Transverse | -3.9386 | 0.0382071764705882 |
|  | 16 | Esophagus_Mucosa | 4.4219 | 0.0149954072727273 | Esophagus_Gastroesophageal_Junction | -5.10043 | 0.000564811666666667 |
|  | 16 | Esophagus_Muscularis | 4.413 | 0.0242002285714286 | Esophagus_Mucosa | -5.78741 | 3.0147975e-05 |
|  | 16 | Heart_Atrial_Appendage | 4.29771 | 0.0325175125 | Esophagus_Muscularis | -4.043 | 0.0398592 |
|  | 16 | Heart_Left_Ventricle | 4.413 | 0.0158349333333333 | Heart_Atrial_Appendage | -4.5329 | 0.005834356 |
|  | 16 | Liver | -4.5601 | 0.00808849125 | Heart_Left_Ventricle | -4.043 | 0.0409845333333333 |
|  | 16 | Skin_Sun_Exposed_Lower_leg | -4.265 | 0.0481466666666667 | Liver | 4.60887 | 0.00466228636363636 |
|  | 16 | Small_Intestine_Terminal_Ileum | -4.525 | 0.0102900282352941 | Lung | -4.942416 | 0.00197962857142857 |
|  | 16 | Testis | 4.492987 | 0.032543316 | Minor_Salivary_Gland | -4.20469 | 0.0163410857142857 |
|  | 16 | Uterus | 4.7571 | 0.00732368 | Skin_Not_Sun_Exposed_Suprapubic | 5.65 | 4.73850666666667e-05 |
|  | 16 | Vagina | 4.98537 | 0.002055468 | Skin_Sun_Exposed_Lower_leg | 5.858 | 3.379896e-05 |
|  | 16 | Whole_Blood | 4.619067 | 0.00535458 | Small_Intestine_Terminal_Ileum | 4.762 | 0.0022242816 |
|  | 16 |  |  |  | Testis | -4.10462 | 0.03129165 |
|  | 16 |  |  |  | Uterus | -5.50693 | 0.0002386735 |
|  | 16 |  |  |  | Vagina | -4.10443 | 0.0269406 |
|  | 16 |  |  |  | Whole_Blood | -5.76759 | 3.727344e-05 |
| PRSS8 | 16 | Adrenal_Gland | -4.62 | 0.01414656 | Adrenal_Gland | 4.798 | 0.00168411428571429 |
|  | 16 | Brain_Anterior_cingulate_cortex_BA24 | -4.58141 | 0.00896082 | Brain_Anterior_cingulate_cortex_BA24 | 4.20419 | 0.0169389238095238 |
|  | 16 | Brain_Caudate_basal_ganglia | -4.016 | 0.0410115047619048 | Brain_Caudate_basal_ganglia | 5.769 | 3.86491866666667e-05 |
|  | 16 | Brain_Cortex | -4.249 | 0.04558 | Cells_Transformed_fibroblasts | -5.243357 | 0.000355093714285714 |
|  | 16 | Brain_Nucleus_accumbens_basal_ganglia | -4.27368 | 0.02772864 | Esophagus_Mucosa | -5.02394 | 0.000609585428571429 |
|  | 16 | Cells_Transformed_fibroblasts | 4.67971 | 0.01128771 |  |  |  |
|  | 16 | Esophagus_Mucosa | 4.4857 | 0.0153269775 | |  |  |
| PYCARD | 16 | Brain_Anterior_cingulate_cortex_BA24 | -4.561 | 0.00863836625 | Adipose_Subcutaneous | 5.18 | 0.000547832571428572 |
|  | 16 | Cells_EBV-transformed_lymphocytes | -4.413 | 0.0165138 | Brain_Anterior_cingulate_cortex_BA24 | 4.802 | 0.00163968384615385 |
|  | 16 | Stomach | -4.73919 | 0.0079232875 | Cells_EBV-transformed_lymphocytes | 4.043 | 0.027354624 |
|  | 16 | Whole_Blood | 4.898941 | 0.00191533028571429 | Stomach | 5.67351 | 4.12748e-05 |
|  | 16 |  |  |  | Whole_Blood | -4.20864 | 0.014297424 |
| RNF40 | 16 | Brain_Amygdala | -4.17136 | 0.0418476666666667 | Esophagus_Mucosa | -4.397 | 0.00757248979591837 |
|  | 16 | Brain_Caudate_basal_ganglia | -4.249 | 0.0223415714285714 | Heart_Atrial_Appendage | 5.3878 | 0.00021442762 |
|  | 16 | Brain_Cerebellar_Hemisphere | -4.328 | 0.01918375 | Ovary | 4.3068 | 0.0127644777777778 |
|  | 16 | Brain_Cerebellum | -4.328 | 0.0223527272727273 | Vagina | -5.10662 | 0.0008727424 |
|  | 16 | Brain_Frontal_Cortex_BA9 | -4.154 | 0.0397319217391304 | |  |  |
|  | 16 | Brain_Hypothalamus | -4.095 | 0.03771836 |  |  |  |
|  | 16 | Brain_Nucleus_accumbens_basal_ganglia | -4.328 | 0.0254858823529412 | |  |  |
|  | 16 | Brain_Substantia_nigra | -4.108 | 0.0442636090909091 | |  |  |
|  | 16 | Cells_Transformed_fibroblasts | -4.12589 | 0.03870072 |  |  |  |
|  | 16 | Esophagus_Gastroesophageal_Junction | -4.154 | 0.040861375 | |  |  |
|  | 16 | Heart_Atrial_Appendage | -5.4377 | 0.000405999 | |  |  |
|  | 16 | Ovary | -4.733763 | 0.00761255 |  |  |  |
| RP11-120K18.2 | 16 | Testis | -4.662145 | 0.0241834233333333 | Testis | 5.27987 | 0.000373761375 |
| RP11-146F11.1 | 16 | Brain_Cerebellar_Hemisphere | -5.009 | 0.00209870225 | Artery_Coronary | 4.63209 | 0.00420312166666667 |
|  | 16 | Brain_Hippocampus | 5.491 | 0.00034896 | Brain_Cerebellar_Hemisphere | 4.573 | 0.0036909535 |
|  | 16 | Skin_Not_Sun_Exposed_Suprapubic | 4.609426 | 0.03501872 | Brain_Frontal_Cortex_BA9 | 6.36941 | 2.65487e-06 |
|  | 16 | Vagina | -4.249 | 0.0357545 | Brain_Hippocampus | -4.605 | 0.00634286117647059 |
|  | 16 |  |  |  | Skin_Not_Sun_Exposed_Suprapubic | -5.77 | 2.7876288e-05 |
| RP11-146F11.5 | 16 | Spleen | -4.525 | 0.018537968 | Spleen | 4.762 | 0.001964288 |
| RP11-196G11.2 | 16 | Adipose_Visceral_Omentum | -4.284 | 0.0333469333333333 | Adipose_Subcutaneous | 5.42568 | 0.0001661183 |
|  | 16 | Brain_Amygdala | -4.647 | 0.02094455 | Adipose_Visceral_Omentum | 5.813 | 5.007477e-05 |
|  | 16 | Brain_Anterior_cingulate_cortex_BA24 | -4.40513 | 0.0130832909090909 | Adrenal_Gland | 5.69 | 9.35736e-05 |
|  | 16 | Brain_Cerebellar_Hemisphere | -4.35056 | 0.0189744727272727 | Artery_Aorta | 6.062 | 1.054714e-05 |
|  | 16 | Brain_Cortex | -4.265 | 0.0494666666666667 | Artery_Coronary | 4.85252 | 0.0021247825 |
|  | 16 | Brain_Frontal_Cortex_BA9 | -5.565328 | 0.0001830463 | Artery_Tibial | 5.88905 | 2.15120133333333e-05 |
|  | 16 | Brain_Hippocampus | -4.27 | 0.031897125 | Brain_Amygdala | 5.5643 | 8.172725e-05 |
|  | 16 | Brain_Hypothalamus | -4.739 | 0.0072062625 | Brain_Anterior_cingulate_cortex_BA24 | 5.75756 | 3.86039366666667e-05 |
|  | 16 | Brain_Nucleus_accumbens_basal_ganglia | -5.63933 | 0.0001234791 | Brain_Caudate_basal_ganglia | 4.52989 | 0.005364575 |
|  | 16 | Brain_Spinal_cord_cervical_c-1 | -4.7605 | 0.0251286 | Brain_Cerebellar_Hemisphere | 5.99998 | 1.5116795e-05 |
|  | 16 | Brain_Substantia_nigra | -4.265 | 0.0406766666666667 | Brain_Cerebellum | 5.88597 | 3.245616e-05 |
|  | 16 | Cells_Transformed_fibroblasts | -4.244 | 0.02768832 | Brain_Cortex | 5.858 | 1.73628e-05 |
|  | 16 | Colon_Transverse | -4.79661 | 0.01275281 | Brain_Frontal_Cortex_BA9 | 5.86203 | 2.12855366666667e-05 |
|  | 16 | Heart_Left_Ventricle | -4.284 | 0.0233713454545455 | Brain_Hippocampus | 5.813 | 4.017402e-05 |
|  | 16 | Liver | -4.244 | 0.030954 | Brain_Hypothalamus | 5.52925 | 0.0004317054 |
|  | 16 | Lung | -4.113 | 0.0484031034482759 | Brain_Nucleus_accumbens_basal_ganglia | 5.03852 | 0.001128883 |
|  | 16 | Minor_Salivary_Gland | -4.244 | 0.036157 | Brain_Putamen_basal_ganglia | 5.069471 | 0.0008974175 |
|  | 16 | Muscle_Skeletal | -4.24899 | 0.0325166 | Brain_Spinal_cord_cervical_c-1 | 5.01629 | 0.001372308 |
|  | 16 | Ovary | -4.096 | 0.0387548 | Brain_Substantia_nigra | 5.858 | 1.903668e-05 |
|  | 16 | Prostate | -4.435 | 0.03294417 | Breast_Mammary_Tissue | 6.02226 | 2.729984e-05 |
|  | 16 | Uterus | -5.249 | 0.001000467 | Cells_Transformed_fibroblasts | 5.806 | 2.87670857142857e-05 |
|  | 16 | Whole_Blood | -4.113 | 0.03625352 | Colon_Sigmoid | 5.86 | 6.8552e-05 |
|  | 16 |  |  |  | Colon_Transverse | 6.01006 | 2.93077e-05 |
|  | 16 |  |  |  | Esophagus_Gastroesophageal_Junction | 5.72552 | 5.14828333333333e-05 |
|  | 16 |  |  |  | Esophagus_Mucosa | 6.062 | 2.260044e-05 |
|  | 16 |  |  |  | Esophagus_Muscularis | 5.89548 | 6.211392e-05 |
|  | 16 |  |  |  | Heart_Atrial_Appendage | 5.1825 | 0.000470443285714286 |
|  | 16 |  |  |  | Heart_Left_Ventricle | 5.813 | 2.85960266666667e-05 |
|  | 16 |  |  |  | Liver | 5.806 | 8.10432e-05 |
|  | 16 |  |  |  | Lung | 5.94 | 3.4105e-05 |
|  | 16 |  |  |  | Minor_Salivary_Gland | 5.806 | 8.41472e-05 |
|  | 16 |  |  |  | Muscle_Skeletal | 5.21587 | 0.0002767692 |
|  | 16 |  |  |  | Nerve_Tibial | 5.83148 | 3.42393e-05 |
|  | 16 |  |  |  | Ovary | 5.999 | 2.754359e-05 |
|  | 16 |  |  |  | Pancreas | 6.062 | 1.950102e-05 |
|  | 16 |  |  |  | Pituitary | 4.57007 | 0.00604294153846154 |
|  | 16 |  |  |  | Prostate | 5.375411 | 0.000182188533333333 |
|  | 16 |  |  |  | Skin_Not_Sun_Exposed_Suprapubic | 5.79 | 4.021952e-05 |
|  | 16 |  |  |  | Skin_Sun_Exposed_Lower_leg | 6.11202 | 1.776612e-05 |
|  | 16 |  |  |  | Small_Intestine_Terminal_Ileum | 6.062 | 1.940454e-05 |
|  | 16 |  |  |  | Spleen | 5.45355 | 0.00015161848 |
|  | 16 |  |  |  | Stomach | 4.85578 | 0.0014741 |
|  | 16 |  |  |  | Testis | 5.769 | 4.61841575e-05 |
|  | 16 |  |  |  | Thyroid | 5.77197 | 2.9603664e-05 |
|  | 16 |  |  |  | Uterus | 5.876771 | 5.466604e-05 |
|  | 16 |  |  |  | Vagina | 4.67202 | 0.00304968615384615 |
|  | 16 |  |  |  | Whole_Blood | 5.94 | 1.98189e-05 |
| RP11-196G11.4 | 16 | Brain_Cerebellum | -4.62 | 0.012589056 | Brain_Cerebellum | 4.798 | 0.00174848 |
|  | 16 | Pancreas | 4.29315 | 0.0232848 | Brain_Substantia_nigra | -5.146 | 0.00040574975 |
|  | 16 | Thyroid | 5.087898 | 0.00228108266666667 | Pancreas | -4.94599 | 0.001575882 |
|  | 16 |  |  |  | Skin_Not_Sun_Exposed_Suprapubic | 5.33 | 0.00017301328 |
|  | 16 |  |  |  | Thyroid | -4.97036 | 0.000901990857142857 |
| RP11-2C24.5 | 16 | Artery_Aorta | -5.43367 | 0.0002896528 | Artery_Aorta | 5.77982 | 2.943754e-05 |
|  | 16 | Artery_Tibial | -5.19785 | 0.00111995533333333 | Artery_Coronary | -6.90966 | 6.771438e-08 |
|  | 16 | Brain_Cerebellum | -4.394228 | 0.0227439 | Brain_Cerebellum | 6.46528 | 1.655592e-06 |
|  | 16 | Heart_Left_Ventricle | -4.095 | 0.0453552615384615 | Brain_Putamen_basal_ganglia | 5.18 | 0.0007489725 |
| RP11-347C12.10 | 16 | Colon_Transverse | 4.206 | 0.0457657777777778 | Brain_Amygdala | 4.71 | 0.00181331764705882 |
|  | 16 |  |  |  | Brain_Anterior_cingulate_cortex_BA24 | 4.99454 | 0.000728220909090909 |
|  | 16 |  |  |  | Cells_EBV-transformed_lymphocytes | -4.665 | 0.00307859076923077 |
|  | 16 |  |  |  | Heart_Left_Ventricle | -4.397 | 0.010978 |
|  | 16 |  |  |  | Whole_Blood | -4.0414 | 0.0273524 |
| RP11-347C12.3 | 16 | Cells_Transformed_fibroblasts | -4.362 | 0.0184493454545455 | Thyroid | 4.61151 | 0.00420088888888889 |
|  | 16 |  |  |  | Uterus | 4.746565 | 0.00208242 |
| RP11-388M20.1 | 16 | Breast_Mammary_Tissue | -5.009 | 0.002170496 | Adrenal_Gland | 5.146 | 0.000559968 |
|  | 16 | Heart_Atrial_Appendage | 4.413 | 0.0340838666666667 | Breast_Mammary_Tissue | 4.573 | 0.003817216 |
|  | 16 | Pancreas | -4.33117 | 0.02153844 | Cells_Transformed_fibroblasts | 4.518 | 0.00426816 |
|  | 16 |  |  |  | Heart_Atrial_Appendage | -4.043 | 0.0352868266666667 |
|  | 16 |  |  |  | Pancreas | 4.78517 | 0.00226233 |
| RP11-388M20.6 | 16 | Adrenal_Gland | 4.30327 | 0.0206304 | Adipose_Subcutaneous | -4.01187 | 0.0324967125 |
|  | 16 | Brain_Amygdala | 4.35755 | 0.027346 | Adrenal_Gland | -4.91973 | 0.001064676 |
|  | 16 | Brain_Substantia_nigra | 4.624 | 0.02294164 | Brain_Amygdala | -4.9328 | 0.001008073 |
|  | 16 | Breast_Mammary_Tissue | 4.31641 | 0.0360521142857143 | Brain_Frontal_Cortex_BA9 | -4.22517 | 0.016697735 |
|  | 16 | Cells_EBV-transformed_lymphocytes | 5.0809 | 0.00162331733333333 | Brain_Substantia_nigra | -4.67 | 0.002623645 |
|  | 16 | Esophagus_Gastroesophageal_Junction | 4.3618 | 0.0241794375 | Breast_Mammary_Tissue | -3.99626 | 0.0329728 |
|  | 16 |  |  |  | Cells_EBV-transformed_lymphocytes | -5.2168 | 0.000589316 |
|  | 16 |  |  |  | Esophagus_Gastroesophageal_Junction | -4.58872 | 0.00371542777777778 |
|  | 16 |  |  |  | Pancreas | -4.72221 | 0.00260834538461538 |
| RP11-388M20.9 | 16 | Brain_Amygdala | 4.39867 | 0.0270974 | Brain_Amygdala | -5.0495 | 0.000611832222222222 |
|  | 16 | Brain_Hypothalamus | 4.5994 | 0.00947428 | Brain_Hypothalamus | -4.55779 | 0.006931419 |
|  | 16 | Small_Intestine_Terminal_Ileum | -5.491 | 0.000231696 | Brain_Putamen_basal_ganglia | -4.267286 | 0.0148445 |
|  | 16 | Stomach | -4.81109 | 0.01105575 | Small_Intestine_Terminal_Ileum | 4.605 | 0.00384914322580645 |
|  | 16 | Whole_Blood | 5.123135 | 0.0010431 | Stomach | 5.75413 | 4.27980366666667e-05 |
|  | 16 |  |  |  | Whole_Blood | -5.52981 | 8.928936e-05 |
| RP11-452L6.5 | 16 | Brain_Anterior_cingulate_cortex_BA24 | -4.064 | 0.0363561888888889 | Brain_Anterior_cingulate_cortex_BA24 | 5.822 | 3.9441185e-05 |
|  | 16 | Lung | -4.624 | 0.0224973333333333 | Colon_Transverse | 5.194 | 0.0004079315 |
|  | 16 | Pituitary | -4.31309 | 0.0370254 | Lung | 4.67 | 0.00415611538461538 |
|  | 16 | Prostate | -4.6572 | 0.01530956 | Pituitary | 3.98309 | 0.0405431111111111 |
|  | 16 |  |  |  | Prostate | 5.413239 | 0.00017713304 |
| RP11-452L6.7 | 16 | Adipose_Subcutaneous | 5.39612 | 0.0005881797 | Adipose_Subcutaneous | -4.4006 | 0.00847996363636364 |
|  | 16 | Brain_Cerebellar_Hemisphere | -4.62544 | 0.00819968285714286 | Artery_Coronary | 4.09968 | 0.0274679142857143 |
|  | 16 | Brain_Frontal_Cortex_BA9 | -4.587284 | 0.0104564616666667 | Brain_Cerebellar_Hemisphere | 5.05258 | 0.0008364115 |
|  | 16 | Brain_Nucleus_accumbens_basal_ganglia | 4.25381 | 0.0275710909090909 | Brain_Frontal_Cortex_BA9 | 4.7337 | 0.00236466153846154 |
|  | 16 |  |  |  | Brain_Nucleus_accumbens_basal_ganglia | -5.03623 | 0.000979992857142857 |
|  | 16 |  |  |  | Brain_Spinal_cord_cervical_c-1 | -4.01108 | 0.0462592941176471 |
| RP11-867G23.3 | 11 | Adipose_Visceral_Omentum | 4.563721 | 0.02734811 | Esophagus_Muscularis | 5.14641 | 0.002208864 |
|  | 11 | Heart_Atrial_Appendage | 4.160115 | 0.04781766 |  |  |  |
| SEPHS2 | 16 | Artery_Coronary | -4.13919 | 0.0405218083333333 | Adipose_Visceral_Omentum | -5.04139 | 0.001076526 |
|  | 16 | Brain_Anterior_cingulate_cortex_BA24 | -4.14654 | 0.0305935066666667 | Brain_Putamen_basal_ganglia | -5.578229 | 0.00016396425 |
|  | 16 | Brain_Hippocampus | 4.362 | 0.0241156285714286 | Lung | -4.397 | 0.00940238095238095 |
|  | 16 | Breast_Mammary_Tissue | 5.53881 | 0.000242048 | Spleen | 5.21363 | 0.000283901 |
|  | 16 | Stomach | -5.009 | 0.008063327 | Stomach | 4.573 | 0.00472694733333333 |
|  | 16 | Vagina | -5.64016 | 0.000226168 | Testis | 4.99671 | 0.001039489 |
|  | 16 |  |  |  | Vagina | 4.14422 | 0.0238771789473684 |
| SETD1A | 16 | Adipose_Visceral_Omentum | -5.15205 | 0.002104119 | Adipose_Visceral_Omentum | 4.85421 | 0.00246703875 |
|  | 16 | Artery_Aorta | -4.5994 | 0.013349216 | Artery_Aorta | 5.67618 | 4.344792e-05 |
|  | 16 | Artery_Coronary | -5.07219 | 0.00136891725 | Artery_Coronary | 5.8266 | 3.943039e-05 |
|  | 16 | Artery_Tibial | -5.01014 | 0.002262088 | Artery_Tibial | 5.53392 | 0.00010412258 |
|  | 16 | Brain_Cerebellar_Hemisphere | -4.867 | 0.00289035166666667 | Brain_Cerebellar_Hemisphere | 5.81714 | 3.06428433333333e-05 |
|  | 16 | Brain_Cerebellum | -5.169271 | 0.00128404 | Brain_Cerebellum | 5.08742 | 0.000661144 |
|  | 16 | Cells_Transformed_fibroblasts | -4.46878 | 0.015476355 | Brain_Cortex | 4.512 | 0.00433058181818182 |
|  | 16 | Esophagus_Gastroesophageal_Junction | -4.525 | 0.01811396 | Breast_Mammary_Tissue | 4.11838 | 0.0209072551724138 |
|  | 16 | Esophagus_Mucosa | -5.2502 | 0.001281816 | Cells_Transformed_fibroblasts | 5.949633 | 2.115954e-05 |
|  | 16 | Heart_Left_Ventricle | -4.89585 | 0.003419647 | Esophagus_Gastroesophageal_Junction | 4.762 | 0.00191936 |
|  | 16 | Liver | -4.2009 | 0.03368358 | Esophagus_Mucosa | 5.61702 | 6.544008e-05 |
|  | 16 | Lung | -4.15432 | 0.0487641666666667 | Heart_Left_Ventricle | 6.337597 | 3.255476e-06 |
|  | 16 | Pituitary | -5.38758 | 0.0005746986 | Liver | 4.05148 | 0.0339235105263158 |
|  | 16 | Testis | 5.421896 | 0.0006837805 | Lung | 5.322583 | 0.0006103 |
|  | 16 | Thyroid | -5.17848 | 0.002117248 | Pituitary | 5.1865 | 0.000861243 |
|  | 16 |  |  |  | Spleen | 5.999 | 3.053854e-05 |
|  | 16 |  |  |  | Testis | -6.16404 | 8.2169555e-06 |
|  | 16 |  |  |  | Thyroid | 5.92581 | 1.95971466666667e-05 |
| SLC5A2 | 16 | Esophagus_Gastroesophageal_Junction | -4.3999 | 0.0231351428571429 | Esophagus_Gastroesophageal_Junction | 5.01162 | 0.00080973 |
|  | 16 | Thyroid | -4.328454 | 0.035445 | Thyroid | 4.20007 | 0.0162818322580645 |
|  | 16 |  |  |  | Vagina | 4.09115 | 0.0271781714285714 |
| SRCAP | 16 | Brain_Caudate_basal_ganglia | -4.5359 | 0.008336004 | Brain_Anterior_cingulate_cortex_BA24 | 5.92868 | 4.140985e-05 |
|  | 16 | Esophagus_Gastroesophageal_Junction | 4.33529 | 0.0243252222222222 | Brain_Caudate_basal_ganglia | 5.14734 | 0.000640112 |
|  | 16 | Esophagus_Mucosa | -4.6262 | 0.01045692 | Esophagus_Gastroesophageal_Junction | -4.00829 | 0.0352959230769231 |
|  | 16 | Skin_Sun_Exposed_Lower_leg | -5.37425 | 0.00069421475 | Ovary | -5.95094 | 1.8477735e-05 |
| STX1B | 16 | Adipose_Visceral_Omentum | -4.69193 | 0.008840562 | Adipose_Visceral_Omentum | 5.82814 | 9.13416e-05 |
|  | 16 | Adrenal_Gland | -4.10906 | 0.0417870857142857 | Adrenal_Gland | 5.16742 | 0.000582072 |
|  | 16 | Artery_Coronary | -5.009 | 0.0015242702 | Artery_Aorta | 5.40577 | 0.000126919875 |
|  | 16 | Esophagus_Gastroesophageal_Junction | -4.27 | 0.0265820454545455 | Artery_Coronary | 4.573 | 0.00515521 |
|  | 16 | Esophagus_Mucosa | -4.474 | 0.01439232 | Artery_Tibial | 4.56252 | 0.00466648055555556 |
|  | 16 | Heart_Left_Ventricle | -4.71373 | 0.00565866 | Brain_Cerebellar_Hemisphere | 4.82802 | 0.001764905 |
|  | 16 | Liver | -4.9217 | 0.0021729708 | Breast_Mammary_Tissue | 5.94 | 2.26176e-05 |
|  | 16 | Muscle_Skeletal | -5.39967 | 0.000336761066666667 | Cells_Transformed_fibroblasts | 4.471883 | 0.00468934615384615 |
|  | 16 | Ovary | -4.513549 | 0.0126150828571429 | Colon_Sigmoid | 4.23 | 0.0211011764705882 |
|  | 16 | Pancreas | -4.096 | 0.0407484 | Colon_Transverse | 4.90955 | 0.001314886 |
|  | 16 | Prostate | -5.0104 | 0.003884622 | Esophagus_Gastroesophageal_Junction | 5.813 | 4.603465e-05 |
|  | 16 | Small_Intestine_Terminal_Ileum | -5.491 | 0.000231696 | Esophagus_Mucosa | 5.52106 | 9.47307e-05 |
|  | 16 | Spleen | -4.8572 | 0.00608724666666667 | Esophagus_Muscularis | 5.40031 | 0.0002208864 |
|  | 16 | Stomach | -4.47319 | 0.018942185 | Heart_Left_Ventricle | 5.39512 | 0.0001914164 |
|  | 16 | Thyroid | -4.424564 | 0.036522528 | Liver | 5.76713 | 5.103189e-05 |
|  | 16 |  |  |  | Minor_Salivary_Gland | 5.62701 | 8.02028e-05 |
|  | 16 |  |  |  | Muscle_Skeletal | 5.45244 | 0.0001073804 |
|  | 16 |  |  |  | Nerve_Tibial | 5.92485 | 2.928115e-05 |
|  | 16 |  |  |  | Ovary | 5.09157 | 0.000701936428571429 |
|  | 16 |  |  |  | Pancreas | 5.999 | 1.4480235e-05 |
|  | 16 |  |  |  | Prostate | 6.175672 | 9.428972e-06 |
|  | 16 |  |  |  | Skin_Not_Sun_Exposed_Suprapubic | 5.94 | 2.47038e-05 |
|  | 16 |  |  |  | Skin_Sun_Exposed_Lower_leg | 5.69 | 5.09552222222222e-05 |
|  | 16 |  |  |  | Small_Intestine_Terminal_Ileum | 4.605 | 0.00384914322580645 |
|  | 16 |  |  |  | Spleen | 5.77869 | 5.777769e-05 |
|  | 16 |  |  |  | Stomach | 5.91838 | 2.3954125e-05 |
|  | 16 |  |  |  | Testis | 5.94 | 2.202005e-05 |
|  | 16 |  |  |  | Thyroid | 6.31655 | 2.523684e-06 |
|  | 16 |  |  |  | Vagina | 5.05263 | 0.000966757333333333 |
|  | 16 |  |  |  | Whole_Blood | -3.99899 | 0.0305016827586207 |
| STX4 | 16 | Artery_Aorta | -5.35068 | 0.0003447498 | Adipose_Subcutaneous | 4.26 | 0.0130514666666667 |
|  | 16 | Brain_Anterior_cingulate_cortex_BA24 | 5.00589 | 0.0015097624 | Adrenal_Gland | 5.10623 | 0.000606018 |
|  | 16 | Brain_Caudate_basal_ganglia | 4.372 | 0.0149117 | Artery_Aorta | 5.44917 | 0.000113792171428571 |
|  | 16 | Colon_Sigmoid | -5.009 | 0.00277146666666667 | Brain_Amygdala | -5.69 | 5.26203333333333e-05 |
|  | 16 | Colon_Transverse | -4.096 | 0.047526 | Brain_Anterior_cingulate_cortex_BA24 | -5.68075 | 4.548295e-05 |
|  | 16 | Esophagus_Muscularis | -4.244 | 0.0405973333333333 | Brain_Cerebellum | 5.28892 | 0.000336036 |
|  | 16 | Liver | -5.1091 | 0.001025703 | Brain_Cortex | -6.062 | 1.98856e-05 |
|  | 16 | Lung | -4.113 | 0.0484031034482759 | Brain_Frontal_Cortex_BA9 | -5.69 | 4.4364275e-05 |
|  | 16 | Muscle_Skeletal | -4.35266 | 0.0225179555555556 | Brain_Nucleus_accumbens_basal_ganglia | -4.35197 | 0.0108315 |
|  | 16 | Pancreas | -4.5078 | 0.01059135 | Breast_Mammary_Tissue | 5.0491 | 0.000783018666666667 |
|  | 16 | Testis | -4.536067 | 0.0332039175 | Cells_EBV-transformed_lymphocytes | 4.6853 | 0.00329687272727273 |
|  | 16 |  |  |  | Colon_Sigmoid | 4.57 | 0.00522228571428571 |
|  | 16 |  |  |  | Colon_Transverse | 5.999 | 1.576279e-05 |
|  | 16 |  |  |  | Esophagus_Gastroesophageal_Junction | 5.18 | 0.000554815 |
|  | 16 |  |  |  | Esophagus_Mucosa | 5.93313 | 2.504601e-05 |
|  | 16 |  |  |  | Esophagus_Muscularis | 5.806 | 5.31456e-05 |
|  | 16 |  |  |  | Liver | 4.71281 | 0.003089772 |
|  | 16 |  |  |  | Lung | 5.94 | 3.4105e-05 |
|  | 16 |  |  |  | Muscle_Skeletal | 5.59339 | 6.745304e-05 |
|  | 16 |  |  |  | Nerve_Tibial | 5.14065 | 0.000569615555555556 |
|  | 16 |  |  |  | Ovary | 4.01468 | 0.0336138571428571 |
|  | 16 |  |  |  | Pancreas | 5.31401 | 0.0002595285 |
|  | 16 |  |  |  | Prostate | 5.027093 | 0.000791709333333333 |
|  | 16 |  |  |  | Skin_Not_Sun_Exposed_Suprapubic | 5.77 | 3.454198e-05 |
|  | 16 |  |  |  | Skin_Sun_Exposed_Lower_leg | 5.69 | 5.09552222222222e-05 |
|  | 16 |  |  |  | Small_Intestine_Terminal_Ileum | 5.48628 | 0.000197907 |
|  | 16 |  |  |  | Spleen | 5.4052 | 0.000165481033333333 |
|  | 16 |  |  |  | Testis | 6.20317 | 1.2817987e-05 |
|  | 16 |  |  |  | Thyroid | 5.822 | 2.745806e-05 |
| SULT1A3 | 16 | Artery_Coronary | 4.1645 | 0.0395190545454545 | Thyroid | 4.271 | 0.0136528888888889 |
| TAOK2 | 16 | Artery_Tibial | 4.27026 | 0.0463347857142857 | Lung | 4.665 | 0.00382520689655172 |
|  | 16 | Ovary | -4.31588 | 0.0200065363636364 | |  |  |
|  | 16 | Pancreas | 4.20873 | 0.0287701615384615 | |  |  |
| TBC1D10B | 16 | Brain_Anterior_cingulate_cortex_BA24 | 4.95221 | 0.00166091966666667 | Esophagus_Mucosa | -4.71 | 0.00246045176470588 |
|  | 16 | Brain_Hippocampus | -4.36677 | 0.0274806 | Whole_Blood | -4.226 | 0.0137921 |
|  | 16 | Esophagus_Gastroesophageal_Junction | -4.90727 | 0.00692769 |  |  |  |
|  | 16 | Vagina | 4.201 | 0.0393207111111111 | |  |  |
| TGFB1I1 | 16 | Colon_Transverse | -4.74233 | 0.0111422066666667 | Colon_Transverse | 4.08292 | 0.022740935483871 |
| TMEM219 | 16 | Adipose_Subcutaneous | 4.37806 | 0.0296125714285714 | Adipose_Visceral_Omentum | -4.11922 | 0.0295151428571429 |
|  | 16 | Colon_Transverse | -4.37843 | 0.031684 |  |  |  |
| TRIM72 | 16 | Artery_Tibial | 4.413 | 0.0282761 | Artery_Tibial | -4.043 | 0.0325267555555556 |
|  | 16 | Heart_Atrial_Appendage | -4.31198 | 0.0405999 | Heart_Atrial_Appendage | 5.4223 | 0.0002210439 |
|  | 16 |  |  |  | Skin_Not_Sun_Exposed_Suprapubic | -4.93 | 0.000899305 |
|  | 16 |  |  |  | Vagina | 5.146 | 0.000884716 |
| VKORC1 | 16 | Adrenal_Gland | 4.413 | 0.0176832 | Adrenal_Gland | -4.043 | 0.0273003789473684 |
|  | 16 | Artery_Coronary | -5.439 | 0.0003734044 | Artery_Coronary | 4.842 | 0.00199706333333333 |
|  | 16 | Brain_Amygdala | 5.62271 | 0.000233684 | Brain_Amygdala | -5.4818 | 0.0001046606 |
|  | 16 | Brain_Anterior_cingulate_cortex_BA24 | 4.413 | 0.01384854 | Brain_Anterior_cingulate_cortex_BA24 | -4.043 | 0.0325848 |
|  | 16 | Brain_Frontal_Cortex_BA9 | 4.503083 | 0.0104021222222222 | Brain_Cerebellum | -5.3376 | 0.00030882528 |
|  | 16 | Brain_Hypothalamus | 4.413 | 0.0143948842105263 | Brain_Cortex | -5.39741 | 0.000167197333333333 |
|  | 16 | Brain_Nucleus_accumbens_basal_ganglia | 4.561 | 0.01225163 | Brain_Frontal_Cortex_BA9 | -5.19271 | 0.0004820685 |
|  | 16 | Brain_Substantia_nigra | 4.17134 | 0.0410834333333333 | Brain_Nucleus_accumbens_basal_ganglia | -4.718 | 0.00312472363636364 |
|  | 16 | Cells_Transformed_fibroblasts | 4.413 | 0.01604664 | Brain_Substantia_nigra | -5.90488 | 4.307659e-05 |
|  | 16 | Heart_Atrial_Appendage | 4.413 | 0.0340838666666667 | Cells_EBV-transformed_lymphocytes | -4.4997 | 0.00518841882352941 |
|  | 16 | Heart_Left_Ventricle | 5.04116 | 0.00215634533333333 | Cells_Transformed_fibroblasts | -4.043 | 0.0218592 |
|  | 16 | Liver | -5.3203 | 0.000438984 | Heart_Atrial_Appendage | -4.043 | 0.0352868266666667 |
|  | 16 | Lung | 4.413 | 0.024412 | Heart_Left_Ventricle | -4.95086 | 0.001475044 |
|  | 16 | Muscle_Skeletal | 5.14607 | 0.001005746 | Liver | 5.05837 | 0.0008927415 |
|  | 16 | Pituitary | 4.59363 | 0.02339576 | Lung | -4.043 | 0.0357645283018868 |
|  | 16 | Skin_Sun_Exposed_Lower_leg | -4.31407 | 0.0481466666666667 | Muscle_Skeletal | -5.77649 | 3.84653733333333e-05 |
|  | 16 | Small_Intestine_Terminal_Ileum | -4.776 | 0.0064802475 | Skin_Not_Sun_Exposed_Suprapubic | 5.15 | 0.000384281333333333 |
|  | 16 | Spleen | 4.67487 | 0.01127931 | Skin_Sun_Exposed_Lower_leg | 5.54992 | 7.37675714285714e-05 |
|  | 16 | Testis | 4.413 | 0.0337751142857143 | Small_Intestine_Terminal_Ileum | 4.96986 | 0.00121278375 |
|  | 16 | Thyroid | 4.413 | 0.0275458285714286 | Spleen | -4.96079 | 0.000769492285714286 |
|  | 16 | Uterus | 4.413 | 0.0190565142857143 | Testis | -4.043 | 0.03824535 |
|  | 16 | Whole_Blood | 5.157167 | 0.001163636 | Thyroid | -4.043 | 0.0285180342857143 |
|  | 16 |  |  |  | Uterus | -4.043 | 0.0300225391304348 |
|  | 16 |  |  |  | Whole_Blood | -5.3937 | 0.0001199565 |
| ZNF48 | 16 | Brain_Cortex | -4.2234 | 0.0447055 | Cells_Transformed_fibroblasts | 4.23626 | 0.0123143586206897 |
|  | 16 |  |  |  | Lung | -3.980515 | 0.0441057142857143 |
|  | 16 |  |  |  | Pancreas | 4.00137 | 0.0352630384615385 |
| ZNF629 | 16 | Adipose_Subcutaneous | 5.33677 | 0.0005447068 | Adipose_Subcutaneous | -4.31206 | 0.0121669043478261 |
|  | 16 | Artery_Coronary | 5.4077 | 0.000296308466666667 | Artery_Coronary | -5.23347 | 0.0005782195 |
|  | 16 | Brain_Caudate_basal_ganglia | 5.2062 | 0.000701941 | Brain_Caudate_basal_ganglia | -5.66135 | 4.3644e-05 |
|  | 16 | Colon_Transverse | 4.10467 | 0.0493539230769231 | Brain_Hippocampus | 4.42338 | 0.01059966 |
|  | 16 | Lung | 4.19429 | 0.049183 | Colon_Transverse | -5.73384 | 5.18561466666667e-05 |
|  | 16 | Ovary | 5.330857 | 0.0006768249 | Lung | -4.82582 | 0.00226822727272727 |
|  | 16 | Small_Intestine_Terminal_Ileum | 4.55 | 0.01293636 | Ovary | -5.15213 | 0.000595163 |
|  | 16 |  |  |  | Small_Intestine_Terminal_Ileum | -4.629 | 0.00379609071428571 |
|  | 16 |  |  |  | Testis | -5.15 | 0.000602654 |
|  | 16 |  |  |  | Whole_Blood | -4.18613 | 0.0151918153846154 |
| ZNF646 | 16 | Adipose_Visceral_Omentum | -5.14674 | 0.001440805 | Adipose_Subcutaneous | 5.769 | 0.00013767378 |
|  | 16 | Adrenal_Gland | -5.13309 | 0.00209988 | Adipose_Visceral_Omentum | 5.40736 | 0.000260976 |
|  | 16 | Brain_Anterior_cingulate_cortex_BA24 | -5.15437 | 0.0008621395 | Adrenal_Gland | 5.94989 | 3.949248e-05 |
|  | 16 | Brain_Caudate_basal_ganglia | -5.0013 | 0.001658472 | Brain_Anterior_cingulate_cortex_BA24 | 5.54343 | 8.064738e-05 |
|  | 16 | Brain_Frontal_Cortex_BA9 | -4.631 | 0.010172344 | Brain_Caudate_basal_ganglia | 6.51422 | 1.0634588e-06 |
|  | 16 | Brain_Nucleus_accumbens_basal_ganglia | -5.35209 | 0.00031375245 | Brain_Frontal_Cortex_BA9 | 4.701 | 0.00241267133333333 |
|  | 16 | Breast_Mammary_Tissue | -4.16764 | 0.04888576 | Brain_Hippocampus | 5.94 | 3.72951e-05 |
|  | 16 | Cells_Transformed_fibroblasts | -4.244 | 0.02768832 | Brain_Nucleus_accumbens_basal_ganglia | 5.40446 | 0.000469365 |
|  | 16 | Esophagus_Muscularis | -4.85741 | 0.00988176 | Brain_Putamen_basal_ganglia | 4.565448 | 0.005600425 |
|  | 16 | Minor_Salivary_Gland | -4.22754 | 0.0344769777777778 | Breast_Mammary_Tissue | 4.98076 | 0.000913361454545455 |
|  | 16 | Muscle_Skeletal | -4.63837 | 0.00758360571428571 | Cells_Transformed_fibroblasts | 5.806 | 2.87670857142857e-05 |
|  | 16 | Pancreas | -4.77121 | 0.005326398 | Colon_Transverse | 5.20225 | 0.000445839142857143 |
|  | 16 | Skin_Sun_Exposed_Lower_leg | -4.265 | 0.0481466666666667 | Esophagus_Mucosa | 4.29286 | 0.0114169846153846 |
|  | 16 | Small_Intestine_Terminal_Ileum | -4.5392 | 0.0116882357142857 | Esophagus_Muscularis | 5.58322 | 0.0001306496 |
|  | 16 | Whole_Blood | 5.114015 | 0.000876204 | Heart_Left_Ventricle | 4.501835 | 0.00784760666666667 |
|  | 16 |  |  |  | Liver | 5.333 | 0.00030581145 |
|  | 16 |  |  |  | Minor_Salivary_Gland | 4.83547 | 0.00194298222222222 |
|  | 16 |  |  |  | Muscle_Skeletal | 6.17102 | 1.0269196e-05 |
|  | 16 |  |  |  | Nerve_Tibial | 6.272 | 6.66076e-06 |
|  | 16 |  |  |  | Pancreas | 4.26665 | 0.0137214 |
|  | 16 |  |  |  | Skin_Not_Sun_Exposed_Suprapubic | 5.43 | 0.0001196184 |
|  | 16 |  |  |  | Skin_Sun_Exposed_Lower_leg | 5.858 | 3.379896e-05 |
|  | 16 |  |  |  | Small_Intestine_Terminal_Ileum | 5.06825 | 0.0009678135 |
|  | 16 |  |  |  | Spleen | 5.769 | 4.07692066666667e-05 |
|  | 16 |  |  |  | Testis | -5.69 | 5.887466e-05 |
|  | 16 |  |  |  | Whole_Blood | -4.63496 | 0.00236436 |
| ZNF668 | 16 | Artery_Tibial | 4.525 | 0.020092664 | Artery_Aorta | -5.22511 | 0.0002739108 |
|  | 16 | Brain_Caudate_basal_ganglia | 4.624 | 0.00911674666666667 | Artery_Tibial | -4.762 | 0.00245656615384615 |
|  | 16 | Cells_Transformed_fibroblasts | 4.113 | 0.038445075 | Brain_Caudate_basal_ganglia | -4.67 | 0.00336842153846154 |
|  | 16 | Colon_Sigmoid | 5.39288 | 0.00052668 | Brain_Substantia_nigra | -4.88106 | 0.000995013846153846 |
|  | 16 | Colon_Transverse | 4.62 | 0.012166656 | Cells_Transformed_fibroblasts | -4.228 | 0.01237584 |
|  | 16 | Esophagus_Mucosa | 4.541 | 0.0134928 | Colon_Sigmoid | -4.84 | 0.00336933333333333 |
|  | 16 | Heart_Left_Ventricle | 4.525 | 0.01054886 | Colon_Transverse | -4.798 | 0.00168981333333333 |
|  | 16 | Liver | 4.62 | 0.00694656 | Esophagus_Gastroesophageal_Junction | -4.76937 | 0.00198148214285714 |
|  | 16 | Lung | 4.525 | 0.0216836 | Esophagus_Mucosa | -4.873 | 0.0011595375 |
|  | 16 | Minor_Salivary_Gland | 4.303 | 0.0317430285714286 | Heart_Left_Ventricle | -4.762 | 0.00335328 |
|  | 16 | Muscle_Skeletal | 4.99278 | 0.001799756 | Liver | -4.798 | 0.0022512 |
|  | 16 | Spleen | 4.45267 | 0.0185905828571429 | Lung | -4.762 | 0.002872 |
|  | 16 | Thyroid | 4.420626 | 0.03100256 | Minor_Salivary_Gland | -4.506 | 0.0054317675 |
|  | 16 | Whole_Blood | 5.559418 | 0.0003769068 | Muscle_Skeletal | -5.41897 | 0.00011324095 |
|  | 16 |  |  |  | Nerve_Tibial | -5.155 | 0.0005940425 |
|  | 16 |  |  |  | Pancreas | -4.6733 | 0.002871792 |
|  | 16 |  |  |  | Skin_Sun_Exposed_Lower_leg | -4.32916 | 0.0104163461538462 |
|  | 16 |  |  |  | Spleen | -5.00895 | 0.000763114727272727 |
|  | 16 |  |  |  | Stomach | -4.09188 | 0.03154574 |
|  | 16 |  |  |  | Testis | -5.58789 | 8.88528333333333e-05 |
|  | 16 |  |  |  | Thyroid | -5.31602 | 0.000250478 |
|  | 16 |  |  |  | Whole_Blood | -5.5111 | 8.27526e-05 |
| ZNF688 | 16 | Artery_Coronary | -4.69672 | 0.00525473142857143 | Brain_Substantia_nigra | -5.0907 | 0.000484052333333333 |
|  | 16 | Brain_Substantia_nigra | 4.45048 | 0.0348599033333333 | Breast_Mammary_Tissue | -5.03682 | 0.0007507456 |
|  | 16 | Vagina | -4.76772 | 0.00412424 | Esophagus_Mucosa | -4.397 | 0.00757248979591837 |
|  | 16 |  |  |  | Thyroid | -6.59231 | 8.185432e-07 |
|  | 16 |  |  |  | Vagina | 4.8191 | 0.00212864 |
| ZNF689 | 16 | Muscle_Skeletal | 4.75213 | 0.00506654 | Artery_Aorta | 5.77993 | 3.919758e-05 |
|  | 16 |  |  |  | Muscle_Skeletal | -4.93442 | 0.000935361230769231 |
|  | 16 |  |  |  | Whole_Blood | 6.39515 | 2.22528e-06 |
| ZNF747 | 16 | Brain_Cerebellar_Hemisphere | -5.24109 | 0.000818506666666667 | Adipose_Subcutaneous | -5.06616 | 0.0008766555 |
|  | 16 | Lung | 4.27526 | 0.0380938888888889 | Brain_Cerebellar_Hemisphere | 5.00597 | 0.000948103555555556 |
|  | 16 | Stomach | 4.78442 | 0.00840237 | Breast_Mammary_Tissue | -5.85673 | 2.49719466666667e-05 |
|  | 16 |  |  |  | Lung | -4.200223 | 0.0208376086956522 |
|  | 16 |  |  |  | Minor_Salivary_Gland | 5.333 | 0.0002116828 |
|  | 16 |  |  |  | Muscle_Skeletal | -4.1106 | 0.0248915833333333 |
|  | 16 |  |  |  | Stomach | -6.0797 | 1.76892e-05 |
|  | 16 |  |  |  | Whole_Blood | -5.16013 | 0.0003435276 |
| ZNF764 | 16 | Brain_Anterior_cingulate_cortex_BA24 | -5.20903 | 0.000859876666666667 | Brain_Anterior_cingulate_cortex_BA24 | 5.51002 | 8.12357166666667e-05 |
|  | 16 | Nerve_Tibial | 4.56 | 0.04761695 | Cells_Transformed_fibroblasts | 4.189694 | 0.0133006909090909 |
|  | 16 |  |  |  | Lung | 4.665 | 0.00382520689655172 |
|  | 16 |  |  |  | Nerve_Tibial | -4.718 | 0.0026194 |
| ZNF768 | 16 | Heart_Atrial_Appendage | -5.08824 | 0.00180945233333333 | Brain_Cerebellum | 4.38119 | 0.007737024 |
|  | 16 | Heart_Left_Ventricle | -4.133 | 0.0416831333333333 | Cells_Transformed_fibroblasts | 6.221008 | 7.771608e-06 |
|  | 16 | Muscle_Skeletal | -4.206 | 0.0357476363636364 | Heart_Atrial_Appendage | 6.1938 | 8.826719e-06 |
|  | 16 | Vagina | 4.362 | 0.0245173714285714 | Whole_Blood | -4.271 | 0.0123275454545455 |
| ZNF771 | 16 | Brain_Hypothalamus | -4.6195 | 0.01032339 | Artery_Aorta | -4.665 | 0.00304017375 |
|  | 16 | Vagina | -5.19261 | 0.001376964 | Lung | -4.623159 | 0.00399123529411765 |
|  | 16 |  |  |  | Thyroid | -4.21048 | 0.0160684 |
|  | 16 |  |  |  | Vagina | 4.25654 | 0.0153735111111111 |
| ZNF785 | 16 | Adipose_Visceral_Omentum | 4.154 | 0.044447475 | Artery_Tibial | 6.18451 | 5.1811795e-06 |
|  | 16 |  |  |  | Cells_EBV-transformed_lymphocytes | 6.4553 | 1.398816e-06 |
|  | 16 |  |  |  | Heart_Atrial_Appendage | 5.94 | 2.1427725e-05 |
| ZNF843 | 16 | Adrenal_Gland | -4.62 | 0.01414656 | Adrenal_Gland | 4.798 | 0.00168411428571429 |
|  | 16 |  |  |  | Cells_EBV-transformed_lymphocytes | 4.8541 | 0.00223884571428571 |
|  | 16 |  |  |  | Esophagus_Mucosa | 5.03464 | 0.0006732345 |
|  | 16 |  |  |  | Nerve_Tibial | 4.15054 | 0.0221847142857143 |
|  | 16 |  |  |  | Pituitary | 4.32388 | 0.0117285428571429 |

Abbreviations: CHR: chromosome; AD: Alzheimer’s disease; Genes in blue are statistically independent genetic effects from all of the shared TWAS genes between AD and Snoring

# Supplementary Figure

## Supplementary Figure1. Quantile-quantile (QQ) plot for Alzheimer's disease (AD), Insomnia, and cross-trait meta-analysis between AD and Insomnia.


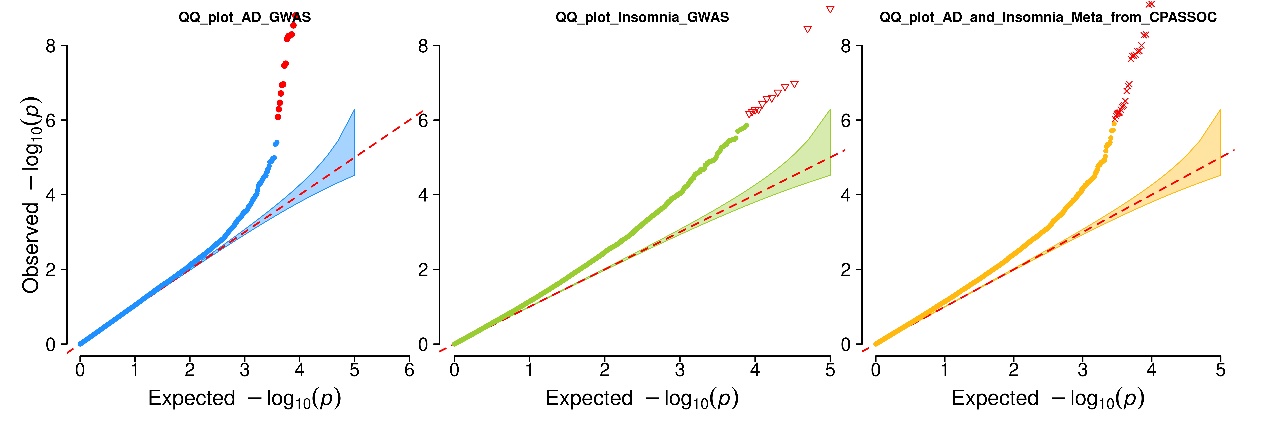


## Supplementary Figure2. Quantile-quantile (QQ) plot for Alzheimer's disease (AD), Sleep duration (Sleepdur), and cross-trait meta-analysis between AD and Sleepdur.


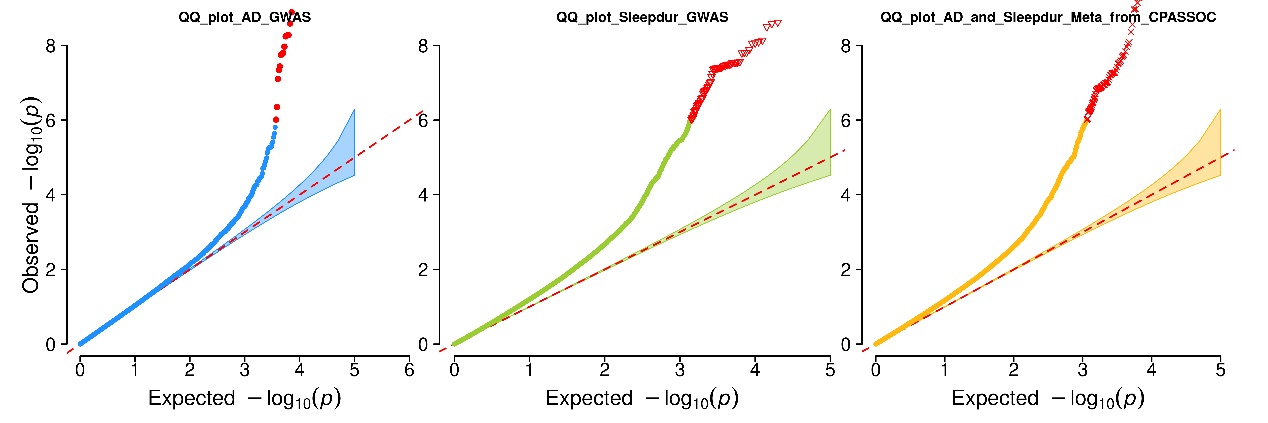


## Supplementary Figure3. Quantile-quantile (QQ) plot for Alzheimer's disease (AD), Snoring, and cross-trait meta-analysis between AD and Snoring.


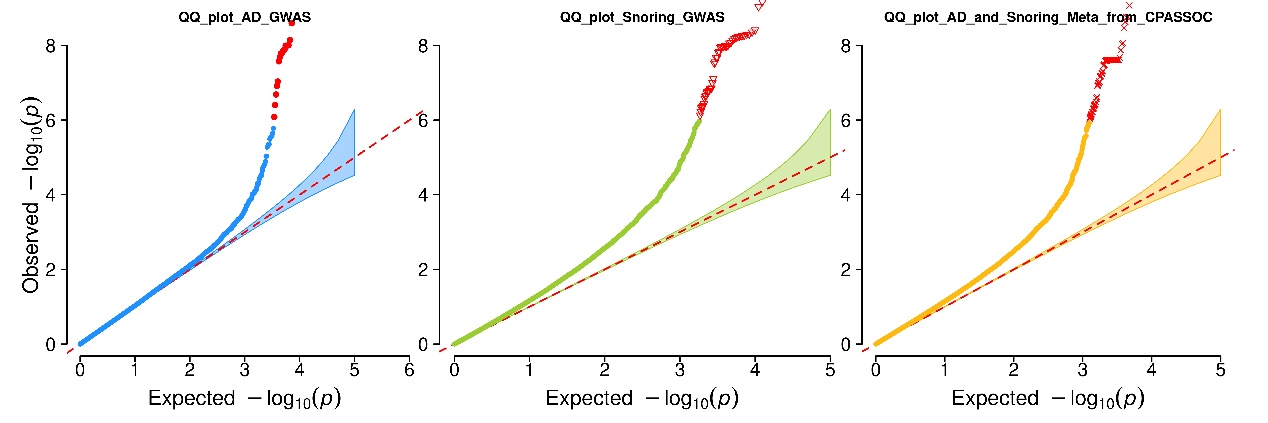


## Supplementary Figure4. Number of significant expression-trait associations from transcriptome-wide association study (TWAS) for Alzheimer’s disease, insomnia, sleep duration, and snoring

The total number is the significant expression-trait associations after Bonferroni correction (false discovery rate < 0.05); GTEx: genotype-tissue expression project; AD: Alzheimer’s disease; Sleepdur: sleep duration


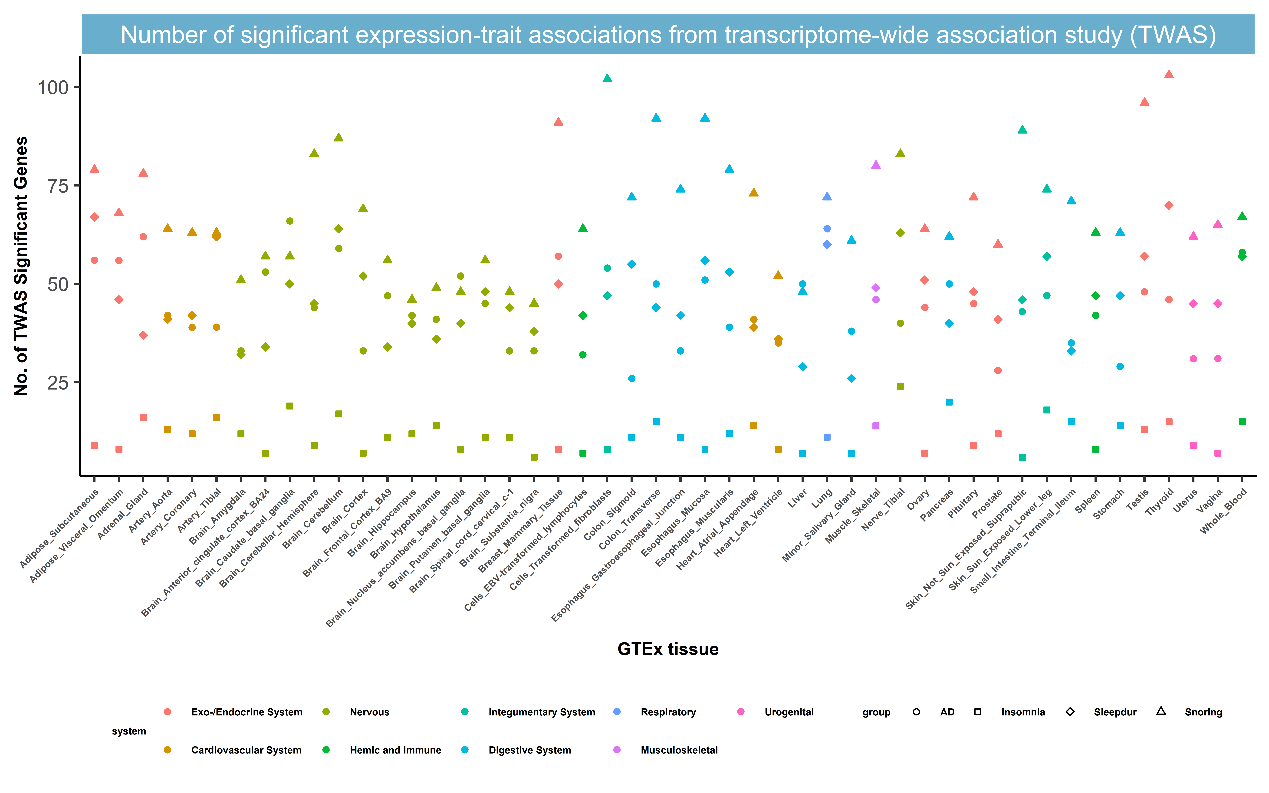

Supplement: Supplementary file 1 [file DataSheet1.docx]
